# The relationship between fear of missing out, digital technology use, and psychological well-being: A scoping review of conceptual and empirical issues

**DOI:** 10.1371/journal.pone.0308643

**Published:** 2024-10-04

**Authors:** Ellen Groenestein, Lotte Willemsen, Guido M. van Koningsbruggen, Hans Ket, Peter Kerkhof

**Affiliations:** 1 Department of Communication Science, Vrije Universiteit Amsterdam, Amsterdam, Netherlands; 2 Research Group Communication in the Networked Society, Creating010/Rotterdam University of Applied Sciences, Rotterdam, Netherlands; 3 Medical Library, Vrije Universiteit Amsterdam, Amsterdam, Netherlands; Aalborg University, DENMARK

## Abstract

Given the rise of digital technology and its assumed impact on psychological well-being, this scoping review systematically examines the literature on Fear of Missing Out (FoMO), which is assumed to play a pivotal role in this dynamic. Although adverse effects of FoMO are commonly assumed, there is still no consensus on the nature of the phenomenon or its relations with psychological well-being and digital technology use, making a scoping review essential. To address this need, we comprehensively assess the conceptualizations of the construct of FoMO and its roles in relation to well-being and digital technology use. We conducted a literature search in PubMed, Ebsco/APA PsycINFO, and Web of Science (period 2013 to July 7, 2023), screening 4121 articles at the title and abstract level and assessing 342 full-text articles for eligibility, ultimately including 106 articles. The review revealed a fragmented FoMO literature, emphasizing the need for conceptual clarity to address critical gaps and inconsistencies in existing research. Consensus exists on FoMO’s essence—an unpleasant feeling arising from missed social experiences driven by activity comparison. However, debates include FoMO’s associated affective states and conceptual boundaries, as well as the need to disentangle FoMO as a trait or state. The review also underscored FoMO’s multifaceted roles in well-being and digital technology use, highlighting the need for causal research, theoretical guidance, and unified terminology to advance understanding in the FoMO literature.

## Introduction

In the present day and age, individuals live increasingly mediated lives. The constant availability of digital technologies such as smartphones, the Internet and social media allows us to be connected to people and information wherever and whenever ‘needed’ [1]. By 2022, there were more than 4.59 billion social media users worldwide, a number that is expected to grow to 5.42 billion in 2025. In 2022, 16–64-year-olds worldwide spent on average almost 2,5 hours per day only on social media and messaging [2]. The potential for 24/7 connectivity is considered as an important force for why people feel they must always be ‘on’ and engage in technology in order to not miss out, stay current, and connect [3, 4]; a psychological phenomenon commonly known as Fear of Missing Out (FoMO).

In their seminal 2013 article, Przybylski and colleagues made a first attempt to conceptualize and operationalize the concept and provide a theoretical account for how the concept is related to psychological well-being and digital technology use [5]. By conducting a survey study, they provided initial evidence that FoMO is associated with lower general mood and general life satisfaction, and higher social media engagement. The latter was explained by the notion that social media use may provide an attractive behavioral strategy for coping with feelings of anxiety stemming from the idea of missing out on activities [5].

Since Przybylski et al. (2013), there has been a proliferation of studies on FoMO. An article search on Google Scholar shows that from 2013 to June 2023, nearly 15,200 papers and articles mention “fear of missing out”, and more than 30,600 its commonly used abbreviation “FoMO”. Despite the large number of studies that have investigated FoMO, there remains a gap in our understanding of what FoMO entails and how it theoretically and empirically relates to psychological well-being and digital technology use. As stated by Tandon, Dhir, Almugren, et al. (2021), the extant literature on FoMO offers “a fragmented view of this phenomenon, its antecedents and its consequences” [6].

Although numerous studies acknowledge the fragmented nature of the literature, there has not yet been an effort to systematically document the extent to which FoMO’s conceptualization, causes and effects vary across different research studies. The aim of this review is to address these criticisms by systematically assessing the literature that has appeared since Przybylski et al. In doing so, we will answer two research questions (RQs):

RQ1: How has FoMO been conceptualized across the extensive body of literature published?
RQ2: What roles are attributed to FoMO in relation to psychological well-being and digital technology use in the research conducted, and to what extent do findings support these roles across studies?

By focusing on the first research question, the current review addresses prior calls in the literature to explore unanswered questions regarding the FoMO construct (e.g., [7]). In the 10 years of research on FoMO, different ways of conceptualizing and measuring FoMO have occurred. The lack of uniformity in definitions and measurement tools may lead to variability in research outcomes and interpretations [8]. The current study will provide a comprehensive examination of the fragmented landscape of FoMO research, offering insights into diverging views regarding FoMO’s conceptualizations, as well as implications for future research.

By focusing on the second research question, the current review aims to gain more insight into the multifaceted roles that FoMO may play in the context of individuals’ psychological well-being and their use of digital technology. Although adverse effects of FoMO on psychological well-being and digital technology use are taken as a given in popular media, there is still no consensus, about what these effects entail and to what extent they are supported by empirical research and theory. Some studies hypothesize that FoMO is an antecedent of digital technology use (e.g., [9–12], while other studies suggested vice versa (e.g., [13, 14]). Similar reciprocal effects have been hypothesized for the relationship between FoMO and psychological well-being (resp. e.g., [15, 16]). This review aims to evaluate the extent to which the various roles of FoMO in the relationship between psychological well-being and digital technology use are supported by empirical research findings, and to elucidate the implications of these findings for future research, providing a foundation for further exploration of these complex relationships in the digital age.

## Methods

This scoping review was conducted in accordance with the JBI methodology for scoping reviews [17], and in line with the Preferred Reporting Items for Systematic Reviews and Meta-Analyses extension for Scoping reviews (PRISMA-ScR; see also S1 Checklist. PRISMA-ScR Checklist) [18]. For transparency, this review was conducted in accordance with an a priori registering with the International Prospective Register of Systematic Reviews (Prospero; CRD42020184146).

### Search strategy

A comprehensive search was performed in the databases PubMed, Ebsco/APA PsycINFO and Clarivate Analytics/Web of Science Core Collection, from inception through July 7, 2023, in collaboration with a medical information specialist (JCFK). The search included controlled terms and free text terms or synonyms of ‘fear of missing out’, indicators of digital technology use such as ‘smartphone use’, or ’internet’ and positive and negative psychological well-being indicators such as ‘life satisfaction’ or ’anxiety’. The full search strategies can be found in the S1 Text. Full Search Strategies. Duplicate articles were excluded by a medical information specialist (JCFK) using Endnote X20.0.1 (Clarivate^tm^), following the Amsterdam Efficient Deduplication (AED)-method [19] and the Bramer-method [20].

#### Inclusion and exclusion criteria

**Fig 1:**
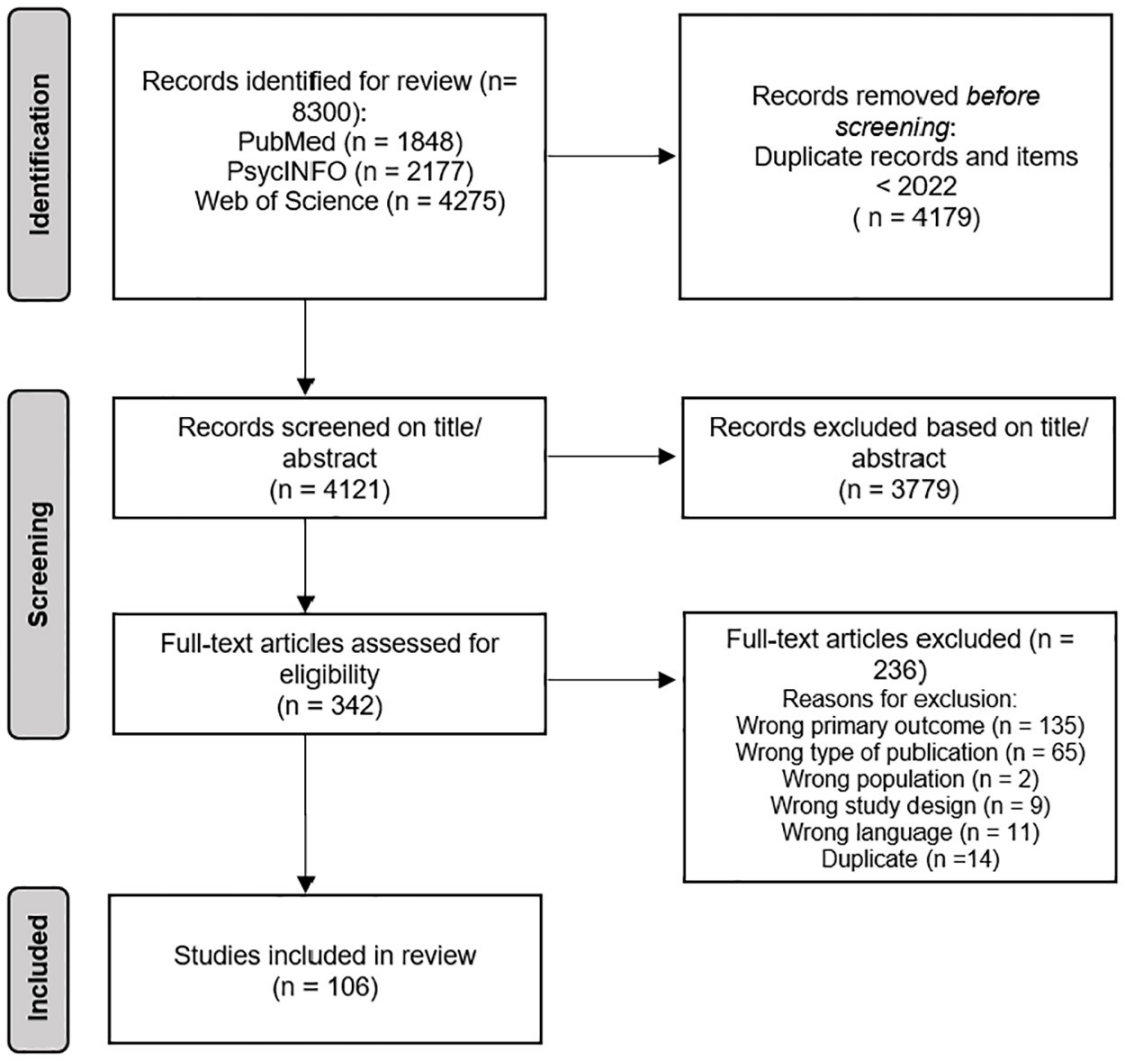
Flow diagram for the review. (MJ, McKenzie JE, Bossuyt PM, Boutron I, Hoffmann TC, Mulrow CD, et al. The PRISMA 2020 statement: an updated guideline for reporting systematic reviews. BMJ 2021;372:n71. doi: 10.1136/bmj.n71).

Fig 1 outlines the process of article selection utilizing the PRISMA guidelines [21]. Studies were included in this review if they examined FoMO in relation to psychological well-being and/or digital technology use. Psychological well-being refers to feeling good and judging life positively [22], and can be seen as a multidimensional construct that encompasses both positive and negative psychological adjustment [23]. This conceptualization includes both positive indicators of psychological well-being, such as self-esteem, social well-being, and life satisfaction, as well as negative indicators, such as depression, loneliness, and anxiety [24]. Digital technology use is an umbrella term that encompasses various devices (e.g., smartphone, laptop), services (e.g., social networking sites such as Instagram and instant messengers such as WhatsApp), and types of use (e.g., active-, passive use) [25]. In our article we included studies that measure a range of concepts related to digital technology use, including Internet use, smartphone use, social media use–time and frequency but also problematic/ excessive use–among other concepts.

We excluded studies that did not focus on the general population (e.g., clinical samples), studies that did not generate any quantitative results (outcomes given in numbers), and studies that were not published in the English language. Also excluded were systematic reviews, literature reviews, and meta-analysis.

#### Search results, study selection and data extraction

Our search resulted in 8300 citations (PubMed N = 1848, PsycINFO N = 2177, Web of Science N = 4275). We included studies up to the year 2022, which yielded 4303 articles, to ensure a thorough exploration of the existing scholarly landscape within a manageable scope. Study selection was accomplished and organized using the Rayyan OCRI software (1). Two reviewers (EG, AvH) independently screened titles and abstracts of the remaining 4303 studies for eligibility (see Fig 1 for flowchart of the search process). This procedure resulted in the identification of 342 potentially relevant articles. These articles either clearly met the inclusion criteria after inspecting the abstract, or required an additional scanning of the full text to determine whether the articles met the inclusion criteria. The full text was then obtained for all included abstracts and subsequently independently evaluated by two reviewers (EG, AvH). Discrepancies were resolved by consensus or adjudication by a third party (LW). This procedure resulted in a selection of 106 relevant articles that met the inclusion criteria.

For each included study, two researchers (EG, TP) independently extracted study characteristics into Microsoft Excel. Extracted data included measures used, hypothesized model, independent variable (IV), dependent variable (DV), mediating variable (M), effects, study and population characteristics and theory used (see S1 Table. Characteristics of Included Articles) [26–63]).

## Results

We conducted a scoping review of the literature to explore the variability in the conceptualization of FoMO since Przybylski et al.’s 2013 study. Additionally, we aimed to uncover the multifaceted roles ascribed to FoMO in relation to psychological well-being and digital technology use. In the subsequent sections, we present the sample characteristics and our findings on the two research questions.

### Sample characteristics

The sample sizes of the studies included in this scoping review varied considerably, ranging from 40 to 5,280 participants. The average sample size across all studies was approximately 659 participants, with a median sample size of 343 participants. The participants’ ages also varied widely, from as young as 9 years old to as old as 80 years. However, the majority of the studies predominantly featured undergraduate students, reflecting a focus on younger demographics, with a mean age of 22 years. Further details on the study characteristics are provided in the S1 Table. Characteristics of Included Articles.

### Conceptual scope and structure of FoMO (RQ1)

In response to RQ1, our scoping review addresses a recognized gap in the literature, echoing prior calls for more research on the nature of the FoMO construct (e.g., [7]). Among the included studies, only a handful of studies have examined the conceptual underpinnings of FoMO [64–68], originally defined by Przybylski et al. (2013) as: “a pervasive apprehension that others might be having rewarding experiences from which one is absent, […] characterized by the desire to stay continually connected with what others are doing” [5]. Most studies only loosely allude to difficulties that may impede our understanding of FoMO. Reviewing these studies, we posit that clarity regarding the conceptualization of FoMO is impeded by (1) a lack of studies that have tested the conceptual structure of the construct, (2) blurring boundaries regarding the range of affective states that can be experienced in relation to FoMO, (3) ambiguity regarding the conceptual boundaries of FoMO, and (4) diverging perspectives as to whether FoMO is best viewed as a trait or a state.

#### Lack of studies that have tested the conceptual structure of the construct

Przybylski et al. ’s definition of FoMO suggests the presence of two underlying dimensions: a “pervasive apprehension for missed social opportunities”, and a “desire to stay continuously connected with what others are doing”. The current literature reveals an ongoing debate about the extent to which both dimensions are defining components of FoMO. While some studies acknowledge both dimensions as defining components of FoMO (e.g., [69]), other scholars focus only on one dimension when describing FoMO. For example, Alt (2018) emphasizes the “pervasive apprehension” component by defining FoMO as: “an anxiety whereby one is compulsively concerned that one might miss an opportunity for social interaction, a rewarding experience, a profitable investment, or other satisfying events” ([70] cf. [71]). Brailovskaia et al. (2021), on the other hand, define FoMO as the desire to be constantly in touch with what others are doing ([72] cf. [1, 73]). Of the 106 included articles in our review, 88 scholars used Przybylski et al.’s original ten-item FoMO-scale (FoMOs). Of them, 12 scholars shortened Przybylski et al.’s original scale: FoMOs, by using only some items (e.g., [74–76]). In doing so, they emphasize the anxiety component of FoMO over the desire to stay continuously connected component, or vice versa. For example, Alt (2018) removed items reflecting the desire to stay continually connected, thereby focusing on the anxiety aspect of FoMO [70]. In contrast, Franchina et al. (2018) used only four out of the ten items of the FoMO-scale, three of which reflected the desire to stay continually connected [77].

In eight articles, including the article in which the scale is developed [68], the Trait-State Fear of Missing Out Scale (T-SFoMOSC) by Wegmann et al. was used to assess FoMO (e.g., [78, 79]). This scale builds on the original FoMO construct by distinguishing between trait FoMO and state FoMO. Trait FoMO refers to a stable predisposition to experience FoMO, whereas state FoMO is a more situational and transient experience related to online activities. In the remaining 10 articles, various (self-designed) scales are used. In some cases, a self-designed questionnaire aim to apply FoMO to domains other than social experiences, extending the concept to include the fear of missing out on news information, referred to as News FoMO (e.g., [80, 81]). In other cases, participants are directly asked if they experience FoMO (e.g., [82]) using single item scales.

Within the FoMO literature only a few studies have conducted a dimensionality assessment to explore the factor structure of FoMOs, which yielded equivocal results. Elhai et al. (2018) conducted a confirmatory factor analysis (CFA) and found a good fit for a one-factor model [83]. The Turkish [84] and Spanish [85] translations of FoMOs also supported the original one-factor structure [5]. However, Al-Menayes (2016) conducted a factor analysis and found a two-factor solution for the Arabic translation of the FoMOs, with one factor reflecting feelings of anxiety and a second factor reflecting a desire to stay continually connected [86]. Similarly, Casale and Fioravanti (2020) found a similar two-factor structure for the Italian translation [87], and Li et al. (2021) for the Chinese translation of the original FoMO-scale [88]. None of these studies compared a one-factor model with a two-factor model, which is necessary to adequately assess the psychometric structure of a construct [89].

#### Blurring boundaries regarding the range of affective states that can be experienced

Within the body of literature on FoMO, we can identify diverging perspectives on what constitutes feelings of “pervasive apprehension”. All studies seem to agree that FoMO refers to an unpleasant state that people experience when they are not engaged in activities or experiences that they consider rewarding (e.g., [66, 90, 91]. This unpleasant feeling is often conceptualized as anxiety (e.g., [92, 93]). In their definition of the concept, Przybylski et al. (2013) limit this feeling to feelings of apprehension, although the authors do not specify what can be understood as an apprehensive experience [5]. In the psychological literature, apprehension is known as one of the two dimensions of anxiety [94]. According to Elhai, Gallinari, Rozgonjuk et al. (2020) apprehension taps into cognitive aspects of anxiety such as worry or concern that something unpleasant might happen ([95] see also [96]). In this sense, apprehension can be distinguished from the second dimension of anxiety, often referred to as fear, i.e., the predominant type of anxiety present in panic, which is characterized by “a set of somatic symptoms distinct from those associated with anxious apprehension e.g., muscle tensions” [97]. Following the argument that FoMO is conceptually distinct from fear, Hayran et al. (2016) suggests renaming the term ‘fear of missing out’ to ‘feeling of missing out’. In line with this perspective, he broadly defines FoMO as “the negative affective state that individuals encounter as a result of becoming aware of […] experiences that are taking place in the environment, from which one is absent” [98]. Schmuck (2021) also argues that FoMO is related to negative affect, as it revolves around a lack of need satisfaction [78].

Indeed, the literature indicates that FoMO is linked to negative emotional states, rather than a mere absence of enjoyment from missing out on experiences perceived as rewarding. This was demonstrated in Milyavskaya et al.’s (2018) mixed-method study, which involved a longitudinal experience sampling method with nightly diaries and end-of-semester measures among 151 college freshmen (M = 18.0, SD = 1.04). The study showed that FoMO was significantly associated with negative affect, but not with positive affect [66]. There is some debate about the range of negative states that could be experienced in relation to FoMO: some scholars mention negative affective states other than anxiety that may be experienced when FoMO occurs, such as feelings of social exclusion [77, 91, 99], distress (1) envy [75, 91, 100–102] and (anticipated/ post) regret [66, 103]. Hayran (2020) made an effort to clarify how, if at all, FoMO relates to these concepts, by examining FoMO in a nomological net [104]. Hayran’s experiments indicate that while other negative emotional states can coincide with FoMO, they don’t always manifest when FoMO is experienced. The author thereby concludes that FoMO is conceptually distinct from social exclusion, envy and (anticipated/post) regret [104]. However, Hayran measured FoMO with a single item (cf. [66]), i.e., "In this moment, to what extent do you feel like you are missing out on alternative activities and experiences taking place in your environment?". This measure does not mention any negative affective state that other scholars consider to be as defining components of FoMO, such as anxiety and apprehension (e.g., [93, 105], which can also be found in Przybylski et al.’s FoMO-scale (e.g., ’I fear […]’, ’I get anxious […]’ ’It bothers me […]’, ’ I get worried […]’ [5]. As such, Hayran measures FoMO as a cognitive rather than a negative affective state, which may explain why FoMO was not related to other affective states such as fear. It is therefore premature to claim, based on Hayran’s study alone, that FoMO is characterized by other affective states than fear or anxiety.

#### Ambiguity regarding the conceptual boundaries of FoMO

In Przybylski et al.’s original conceptualization, FoMO is felt in relation to missed experiences. However, there are differing views on the nature of the missed experiences that may trigger the experience of FoMO. Some scholars (e.g., [67, 95, 106, 107]) argue that people are particularly afraid of missing out on social experiences, which are generally perceived as rewarding. This is also reflected in Przybylski et al.’s emphasis on the need to be continuously connected to what others are doing, which the authors argue is part of the FoMO experience [5]. Indeed, all ten items of Przybylski et al’s FoMO-scale refer to missed social experiences (e.g., “I get worried when I find out my friends are having fun without me”, “I fear my friends have more rewarding experiences than me”, “I get anxious when I don’t know what my friends are up to”) [5]. Some scholars add a comparative element (e.g., [64, 108]) by arguing that in order to experience FoMO, a missed social activity should be perceived as superior to the current activity the person is engaged in. These two perspectives are synthesized by Neumann (2020), who argues that FoMO involves two types of comparisons: (1) one’s own experience with those of others (cf. [1]), and (2) one’s current experience with an alternative that is perceived as more enjoyable, pleasurable, or rewarding (cf. [92, 109]).

Against this background, it is not surprising that some scholars limit FoMO to the context of social media. Social updates from others remind people of unattended alternative experiences in real time and are therefore likely to evoke FoMO. Similarly, Dossey (2014) argues that FoMO is: “a compulsive concern that one might miss an opportunity for social interaction, a novel experience, or some other satisfying event, often aroused by posts seen on social media sites” ([110] cf. [111]). This perspective stems in part from the fact that the most commonly used FoMO-scale (FoMOs; [5]) includes one item that refers to the online context, e.g., “When I have a good time it is important to me to share the details online (e.g., updating status)”, whereas Przybylski et al.’s original definition of FoMO does not specifically refer to social media use [87].

Milyavskaya et al. (2018) explored the conceptual boundaries of FoMO by examining whether one would experience FoMO in the case of (1) learning about missed non-social versus social activities, (2) while engaged in an obligatory versus a more rewarding alternative activity (e.g., studying versus meeting a friend), and (3) whether awareness of unattended alternative experiences needs to be established through social media exposure or whether FoMO occurs even if awareness is established otherwise [66]. Based on a vignette study, the authors report that FoMO is more prevalent when one misses out on a social (vs. non-social) activity, a finding that supports a second study by the authors in which FoMO is found to increase during social peak moments, i.e., moments later in the day and week (Thursdays, Fridays, and Saturdays). In addition, the results showed that people experienced FoMO regardless of whether the missed opportunity was communicated via social media or through direct contact with a friend, people equally experienced FoMO [66]. This suggests that FoMO can occur outside the context of social media, although social media can still increase people’s awareness of missed opportunities. This is supported by studies that show that people experience FoMO when they fear that they are missing out on physical gatherings and conversations with peers (e.g., [112, 113]). In the study by Milyavskaya et al. (2018), people also experienced FoMO regardless of whether they were engaged in an obligatory activity (e.g., studying) or an activity that was classified by the authors as self-chosen and more rewarding (e.g., watching a TV show, going to a party) [66]. However, the study design does not provide insight into whether people actually perceive each of these activities as rewarding, either in itself in comparison to alternative activities. Hayran et al. (2016), however, investigated self-relevance when considering the alternative activities of others, that is, the degree to which people perceive an activity as relevant to one’s self and life experiences. Based on the results of five experiments, the authors argue that FoMO is driven by the perception of favorable and *self-relevant experiences* taking place in one’s environment [98].

Zhang and colleagues (2020) propose two different conceptualizations of FoMO. Drawing on self-concept theory, which argues that a person’s self-concept consists of a public and a private self, the authors propose that people fear not only missing out on experiences that other people enjoy (social FoMO), but also experiences that they had wanted for themselves (personal FoMO) [114]. In line with this perspective, the authors developed a new scale consisting of two dimensions, i.e., a social and a personal FoMO, which showed good psychometric properties in four studies.

Other alternative scales have also been proposed, reflecting the perspective that FoMO may be experienced in relation to both social and non-social, but personally relevant, experiences. For example, Alt (2015) suggests that FoMO can also be experienced in relation to missing an opportunity for a profitable investment, a promotional offer, or news update [80]. Therefore, they extended the original FoMO-scale to include three dimensions: social FoMO (the original FoMO-scale), news information FoMO, and commercial information FoMO. In several studies (e.g., [70, 80, 81]), positive association results were found among the factors studied. Stretching the conceptualization of FoMO to a fear of missing out on news information and commercial information creates conceptual overlap with constructs such as news dependence (i.e., strong interest in and high consumption of news [115]), and market mavenism (i.e., a social-oriented tendency to acquire extensive knowledge of the market, including information about products, services, stores, best deals and buying in general [116, 117], each of which comes with its own causes and consequences.

In summary, the conceptual boundaries of FoMO continue to be a topic of ongoing scholarly debate. While initially linked to missed rewarding experiences, researchers have introduced diverse perspectives on what constitutes these rewarding experiences. While most emphasize that these missed experiences are primarily social in nature, there remains disagreement regarding the definition of what is truly rewarding and the role of comparison in making this assessment. Specifically, the debate centers on whether social experiences are inherently rewarding, or whether they acquire this quality only when they are superior to one’s current activities.

#### Diverging perspectives as to whether FoMO is best viewed as a trait or a state

The above discussion alludes to the idea that FoMO is a state that is triggered by specific circumstances in which someone finds themselves at a particular moment in time (e.g., seeing social updates from others). This introduces a perspective on the nature of FoMO that challenges Przybylski et al.’s original conceptualization of FoMO as a trait: i.e., a factor that varies across individuals and remains relatively stable over time. Przybylski et al. (2013) initially described FoMO as an individual disposition, arising from the feeling that others are having rewarding experiences one is absent from [5]. Their FoMO-scale was therefore intended to measure individual differences in FoMO proneness.

While some researchers have embraced this perspective (e.g., [12, 77, 79]), no study to date has empirically tested whether FoMO should be viewed as a trait or as a state. Initial conclusions about the nature of FoMO can only be drawn from longitudinal studies. As part of the longitudinal design, these studies have examined whether FoMO is stable or more volatile over time, thereby providing preliminary insight into whether FoMO can be considered as a trait. In a study among 506 Facebook users aged 13–77 years (M = 20.7, SD = 9.10, Buglass et al. (2017) examined FoMO in two waves, with 6 months in between. They found that the relationship between FoMO measured at wave 1 and FoMO wave 2 was only moderate in magnitude (*r* = .55, *p* < .001) [64]. Lo Coco et al. (2020) also used a two-wave longitudinal survey (1 year apart), using two subscales of FoMO and showed positive but weak relationships between time 1 and time 2 measures (β = .19, *p* < .05; β = .35, *p* < .001) among university students (N = 506, M = 20.7, SD = 9.10) [67]. These results suggest that FoMO may not be as stable as one would expect from a trait.

Other, more indirect evidence comes from scholars who have examined FoMO in relation to other personality traits such as the Big-5. More specifically, these scholars have tested the extent of the correlation, if any, between FoMO the Big-5 personality traits–i.e., extraversion, neuroticism, conscientiousness, agreeableness, and, openness–and personality traits such as narcissism (e.g., [10, 66, 91, 108, 118–120]). The results show mixed results between personality traits and FoMO, ranging from small to moderate effect sizes, or no effect at all (see S1 Table. Characteristics of Included Articles), which again does not provide strong evidence for FoMO as a trait.

Wegmann et al. (2017) reconcile both perspectives by arguing that FoMO is best be understood as a complex multidimensional construct that consisting of (1) dispositional trait-FoMO, which entails a general tendency to worry and experience anxiety about missing out on others people’s experiences, and (2) state-FoMO, which refers to a state that can be triggered by circumstances, specifically by online content and interactions with others [68]. In line with this perspective, Wegmann et al. (2017) conducted a study with 270 individuals aged 17–39 years (M = 23.43, SD = 4.02) and created a twelve-item FoMO scale intended to measure trait-FoMO and state-FoMO, which showed good psychometric properties [68]. Using this scale, Balta et al. (2018) showed that trait-FoMO does not fully predict state-FoMO in a sample of 423 Instagram users aged 14–21 years (M = 17.15, SD = 2.24), suggesting that factors other than those residing solely within an individual can trigger state-FoMO [118].

Thus, scholars have divergent thoughts regarding FoMO’s stability over time and its associations with personality traits. A multidimensional perspective, introduced by Wegmann et al. (2017), suggests both trait-FoMO and state-FoMO, offering a more comprehensive understanding of this phenomenon [68].

### The multiple roles of FoMO in relation to psychological well-being and digital technology use (RQ2)

In addition to the absence of a shared understanding of what FoMO entails, there is also a lack of understanding regarding how FoMO is related to psychological well-being and digital technology use. By focusing on the second research question, the current review aims to gain more insight into the multifaceted roles that FoMO may play in the context of individuals’ psychological well-being and their use of digital technology. Based on the literature, we posit that such an understanding is impeded by (1) uneven attention to various roles ascribed to FoMO (2) the prevalence of correlational research, (3) a dearth of theoretical guidance, and (4) heterogeneity in the operationalization of psychological well-being and digital technology use.

#### Uneven attention to various roles ascribed to FoMO

**Fig 2:**
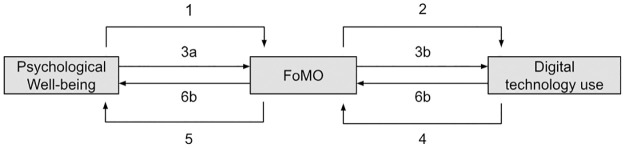
Roles of FoMO in its relationship with psychological well-being and digital technology use.

Since FoMO was introduced to the literature, research has proposed several hypotheses regarding the role of FoMO in the relationship between psychological well-being and digital technology use. Fig 2 visualizes all roles that have been attributed to FoMO in relation to psychological well-being and digital technology use in the included articles.

The various roles that can be ascribed to FoMO stem from the idea that FoMO is a motivational force for digital technology use arising from deficits in psychological well-being, as initially proposed by Przybylski et al. (2013). The studies that have appeared since then have examined FoMO as a consequence of psychological well-being (path 1, e.g., [121, 122]) and/or as an antecedent of digital technology use reflecting the idea that FoMO may drive people to seek solace in digital technology use with increased levels of social media or smartphone use as the result (path 2, e.g., [123, 124]). Along these lines, FoMO is also hypothesized to act as a mediator in some studies to explain the effect of psychological well-being on digital technology use (path 3, e.g., [96, 125]).

Three more roles have been identified for FoMO, that allude to the commonly held idea, initially proposed by JWT intelligence (2011) and echoed by popular media, that digital technologies such as social media constantly remind people of socially rewarding situations in which they cannot participate [5]. Feelings of missing out that may arise from this continuous stream of social information, are believed to impair people’s well-being, with reduced feelings of life satisfaction and stronger feelings of anxiety and depression as the hypothesized result (e.g., [13, 126]. Studies that follow this line of thinking have examined FoMO as a consequence of technology use (path 4, e.g., [1, 127] and an antecedent of deficits in well-being (path 5, e.g., [16, 128]). Additionally FoMO is proposed to serve as a mediator explaining the hypothesized effects of technology use on well-being (path 6, e.g., [13, 91]).

The literature review shows that scholarly attention is not evenly distributed across the six identified paths between FoMO, psychological well-being and technology use. The direct path from psychological well-being to FoMO (path 1) has been examined in 19 articles, the path from FoMO to digital technology use (path 2) in 74 articles, and the path in which FoMO mediates the relationship between psychological well-being and digital technology use (path 3) in 15 articles. In contrast, the direct path from FoMO to digital technology use (path 4) was examined in 16 articles, the path from FoMO to well-being (path 5) in 24 articles, and the mediated path (path 6) in 7 articles. In 4 articles, the authors did not specify the direction of the hypothesized relationship between psychological well-being and FoMO, and in 12 articles, the authors did not specify the direction of the relationship between digital technology use and FoMO.

#### The prevalence of correlational research

In line with the hypotheses that have been proposed, studies suggest that psychological well-being is negatively related with FoMO (path 1), while FoMO is positively related to technology use (path 2). Inverse relations have also been reported with technology use being positively related to FoMO (path 4), and FoMO negatively related to well-being (path 5). These findings suggest that effects can operate in both directions. With the exception of four studies [66, 127, 129, 130], most of the research conducted in this area has been correlational in nature, limiting our ability to make causal claims about the direction of the observed relationships. Only four studies used an experimental design [106, 126, 129, 130] and six used a longitudinal design measuring the variables of interest at multiple (two waves) points in time [64, 66, 67, 78, 131, 132].

Experimental studies were only conducted to test the path from digital technology use to FoMO (path 4), and the path from FoMO on psychological well-being (path 5). Brown and Kuss (2020) conducted an experimental study with 61 participants aged 20–49 years, focusing on the impact of seven days of social media abstinence and its downstream consequences for well-being to test path 4. Their findings revealed significant decreases in FoMO following an abstinence period [129]. Eide and colleagues (2018) also conducted an experimental study, with 127 smartphone users aged 18–48 years (M = 25.0, SD = 4.8), to test path 4. The results of their 72-hour smartphone restriction experiment showed that restricted smartphone use led to increased FoMO, suggesting that the inability to interact with one’s smartphone may also provoke FoMO [130]. Additionally, Fitz et al., (2019) showed in their randomized field experiment involving 237 MTurk smartphone owners (M = 30.3) that receiving no notifications at all–compared to receiving notifications as usual–led to higher levels of phone-related FoMO (C-FoMO-Scale; [133]). However, the indirect effect of never receiving notifications on anxiety via FoMO was not significant (path 5) [126].

Although in correlational research increased social media use is typically associated with more FoMO, in experimental research decreased social media is also found to contribute to more FoMO. This may indicate that the relationship between digital technology use and FoMO follows an inverted U-shape, such that people experience more FoMO when they are unable to use digital technology as well as when they use digital technology extensively. However, an alternative explanation may be provided by the idea that both limited use and excessive and uncontrolled use may threaten feelings of autonomy, one of the three basic human needs according to STD [134], which, if not met, are thought to lead to FoMO [5].

Milyavaskaya et al. (2018) reported the results of two studies that aimed to test path 6. In the first study, the authors used nightly diaries and end-of-semester measures of 151 college students (M = 18.0, SD = 1.04). They showed that those who experienced FoMO more frequently also reported increased levels of negative affect and stress at the end of the semester compared to the beginning of the school year. In a second experimental study among adult participants, the authors showed that experiencing FoMO led to a number of negative outcomes, such as increased negative affect and decreased positive affect in adult participants. Both results emphasize FoMO’s impact on well-being [66].

The two longitudinal studies that were conducted focused mostly on examining potential reciprocal relations between FoMO and technology use (path 2 and path 4) and were restricted to two-wave data. In a study among 242 adolescents aged 12–16 years (M = 14.16, SD = 0.99), Lo Coco et al. (2020) examined the cross-lagged relationship between two domains of FoMO; fear and control [87] and problematic smartphone use (PSU) using a two-wave panel model with a one-year interval. The researchers hypothesized that FoMO could lead to increased problematic social media use. However, the reverse causal effect was also hypothesized, such that an increase in PSU would be expected to lead to an increase in FoMO. The results of the bidirectional relationships examined showed that FoMO and problematic smartphone use were positively related at both points in time. However, the bidirectional findings were not supported longitudinally [67].

In another two-wave longitudinal study amongst 506 Facebook users aged 13–77 (M = 20.7, SD = 9.10), aimed to test path 6, Buglass et al. (2017) showed that an increase in self-reported social media use led to increased levels of FoMO six months later. Moreover, significant indirect effects were observed between SNS use, FoMO, and psychological well-being as measured by self-esteem. The longitudinal results also hinted at a potential cyclic relationship between the main study variables, with low self-esteem at T1 appearing to drive FoMO six months later [67].

To conclude, the current body of research on FoMO and its relationship with psychological well-being and technology use has primarily relied on correlational studies, limiting our ability to establish causal claims (cf. [66]). The few experimental findings supported the impact of FoMO on digital technology use (path 2), as well as the association between restricted technology use and increased FoMO (path 4). However, it is important to note that the results across studies were not consistent, as Lo Coco et al. (2020) did not find cross-lagged support for both of these pathways in their longitudinal study [67]. Also, none of the longitudinal studies employed three wave designs which are better suited to establish causal effects [135]. Nevertheless, there is longitudinal evidence supporting the relationship between digital technology use, FoMO, and well-being (path 6), emphasizing the significant influence of FoMO on psychological well-being.

#### A dearth of theoretical guidance

Although some scholarly works analyzed in this review rely on theory to substantiate their hypotheses, there is a noticeable deficit in the use and advancement of theory. In general, it appears that about 45% of studies are theory driven. The other studies merely refers to the role of FoMO within a nomological web without clarity regarding a specific theory or conceptual framework, highlighting a dearth of theoretical guidance for understanding FoMO’s role in the relationship between digital technology usage and psychological well-being. Some hypothesized paths (see Fig 2) are better substantiated with theory than others: 15 of 19 articles in path 1, the path from psychological well-being to FoMO, refer to theory, 38 of 74 articles in path 2, the path from FoMO to digital technology use, 4 of 16 articles in path 4, the path from digital technology use to FoMO, and 10 of 24 articles in path 5, the path from FoMO to psychological well-being.

In theorizing about FoMO, scholars have primarily adopted Przybylski et al.’s perspective that departs from Self-Determination Theory (SDT; [136]) and explains FoMO from a motivational lens. SDT holds that individuals’ purposeful, goal-driven actions are driven by three intrinsic psychological needs that they seek to fulfill in relation to others: the need for autonomy, competence, and relatedness. According to Przybylski et al. (2013), FoMO arises from deficiencies in one or more of these fundamental psychological needs, as these needs must be satisfied for people to function in an optimal and healthy way [5]. Surprisingly, none–except one–of the included studies have examined the relation between psychological needs and FoMO. Studies loosely allude to SDT without actually subjecting it to empirical testing. This holds for articles examining path 1 predicting FoMO as a consequence of deficits in psychological well-being, as SDT seems to suggest, but also for articles examining the reversed relationship (path 5), as well as other paths (paths 2; the path from FoMO to digital technology use and 3, the path in which FoMO mediates the relationship between psychological well-being and digital technology use). The only study that examined the relationship between psychological need satisfaction was correlational. This study, which included 306 university students aged 18–30 years (M = 21.8, SD = 3.19), showed moderate associations between psychological need satisfaction and FoMO (i.e., autonomy *r* = -.50, competence, *r* = -.46, relatedness, *r* = -.43; [137]).

In line with the motivational lens that Przybylski et al. propose, the Compensatory Internet Use Theory (CIUT; [138] is mentioned to explain the relationship between FoMO and technology use (path 2), and vice versa (path 4). CIUT is a psychological framework that was originally proposed to explain (problematic) internet use [138], but has also been applied to explain social media use and smartphone use (e.g., [123, 139]. The theory argues that people turn to digital technologies to compensate for unmet needs or deficits in their offline lives and cope with emotional or psychological issues. For example, individuals experiencing loneliness may engage excessively in social media to fulfill their need for social connection, using these platforms as a means to compensate for their lack of offline social interactions.

A more comprehensive framework that is believed to explain interrelations between FoMO, psychological well-being and digital technology use, is the Interaction of Person-Affect-Cognition-Execution (I-PACE; [140]); a process model grounded in the addiction literature, predicting problematic technology use from a dynamic interplay of a person’s core characteristics and cognitive, affective, and executive processes. The first level of the model comprises of core characteristics such as personality traits (e.g., impulsivity and low conscientiousness), psychopathological symptoms (e.g., depression and social anxiety), use motives, biopsychological factors (e.g., sex and age) and social cognitions (e.g., loneliness and perceived lack of social support), as well as an individual’s subjective perception of situational factors. The second level of the model includes mediators and moderators that can either amplify or mitigate the influence of core characteristics, ultimately shaping an individual’s decision to use technology to satisfy their needs or seek gratification. These factors include cognitive biases linked to technology (i.e., unrealistic expectations about the outcomes of using technology, leading to the expected gratification of pleasure, for instance), as well as cognitive and emotional responses such as those observed in other addictive behaviors (e.g., craving, urge for mood regulation, attentional bias, cue-reactivity). The feeling of gratification strengthens factors residing on the first and second level of the model, resulting in a reinforcing cycle that ultimately leads to addictive use of digital technology at the third level.

Within the I-PACE model, FoMO has been attributed different roles by various scholars. For instance, Wegmann et al. (2017) (cf. [141]) conceptualize FoMO as a cognitive bias, defined as "the expectation of experiencing pleasure or avoiding negative emotions when using Internet-communication applications such as social media" [68]. In the same line, Elhai, Yang, Fang, et al. (2020) [125] and Elhai, Yang, Rozgonjuk, et al (2020) [142] views FoMO as a cognitive biases, although Elhai, Gallinari, Rozgonjuk, et al. (2020) [95], sees FoMO also as an affective response. While these authors ascribe different roles to FoMO, they share the hypothesis that FoMO serves as a variable linking psychological well-being to digital technology use (paths 1, 2, and 3). On the other hand, Fabris (2020), presents an alternative perspective, arguing that FoMO can be regarded as a core characteristic, specifically a need or motive, that leads to decreased well-being manifested as emotional distress [128] (path 5; cf. [143]). Ambiguity regarding the role of FoMO in the relationship between psychological well-being and digital technology thus seems to stem from ambiguity in the conceptualization of FoMO (see RQ1).

In addition to these theories, various other theoretical approaches have been mentioned to explain the relationship between FoMO, psychological well-being and digital technology use, including Social Comparison Theory (SCT; [144]), Differential Susceptibility to Media Effects Model (DSMM; [145]), and Threaded Cognition Model (TCM; [146]). Since these theories are referenced in the context of hypothesis development but not empirically tested, there is a limited empirical basis to confirm their value in explaining hypothesized relationships. This hinders the construction of theory development to guide our understanding of FoMO and the role it plays in the relationship with psychological well-being and digital technology use.

#### Heterogeneity in the operationalization of FoMO’s antecedents and consequences

The review shows that research is inconsistent in how it operationalizes and assesses digital technology use. This can be attributed to various factors, including differences in the specific digital technology behaviors being studied (e.g., engagement with social media use, smartphone use, or general internet browsing) and variations in the timeframes employed for data collection. Some studies focus on immediate effects resulting from brief interactions with specific social media platforms, spanning minutes to hours (e.g., [77]), while others explore the consequences of prolonged usage based on overall social media engagement [147].

Moreover, there is an uneven distribution of attention across paths in terms of the type of digital technology studied. Studies examining FoMO as a consequence of digital technology use (path 4) and those examining FoMO as a mediator in the relationship between technology use and psychological well-being (path 6), have primarily focused on social media use, with less emphasis on smartphone use. In contrast, studies that examined the reversed path–i.e., FoMO as a mediator in the relationship between psychological well-being and technology use (path 3)–focused primarily on various forms of smartphone use, such as average time spent on smartphones and problematic smartphone use, with less attention to social media use. What may further complicate our understanding is the fact that individuals often access social media through their smartphones (3). This is supported by large associations between (problematic) smartphone use and (problematic) social media use (e.g., [148]), indicating that two forms of digital technology use are partly overlapping phenomena (3).

There has not only been limited research on how social media relates to smartphones in their potential to trigger FoMO, but there has also been scant exploration of distinctions between social media. Assuming that platforms differ in the types of users they attract, the types of interactions they facilitate (private vs. public), and the types of content they feature, they are likely to engender differential effects on the manifestation of FoMO. In a large-scale study involving 2,663 high school pupils (M = 14.87, SD = 1.67), Franchina et al. (2018) offers preliminary insights, indicating that FoMO was a stronger predictor of social media use on more private platforms, exemplified by Facebook and SnapChat, compared to more public platforms like YouTube and Twitter. The authors posit that private platforms, distinguished by more restricted content access, are better suited to provide relief from FoMO, due to their ability to provide users with a sense of relief from anxieties associated with staying informed about the activities of friends and family. In contrast, platforms with content accessible to a broader and predominantly unknown audience may not offer the same level of reassurance [77].

Another study by Fumagalli et al. (2021), involving 334 young adults (M = 21.50, SD = 2.03), aligns with this notion, suggesting that the nature of social network usage can significantly impact feelings of FoMO [149]. The findings indicate that social networking apps like Facebook and Instagram, characterized by passive content consumption enabling users to observe others’ activities, are associated with increased feeling of FoMO. In contrast, interactive messaging apps like WhatsApp or iMessage, which facilitate a more direct form of peer-to-peer communication, are not linked to heightened FoMO.

The nature of the content being viewed may be particularly imperative to study, as people might feel a sense of missing out only if exposed to content illustrating socially rewarding situations from which they were excluded. Hence, the predominant focus on digital technology use in terms of time spent, motivations, and problematic use, should be broadened to also include the specific content being consumed.

Finally, most studies rely on self-reported technology use. Only four studies [131, 149–151] have employed objective measures of digital technology use such as providing screenshots to display usage, while all other studies have relied on self-report measures. In a study involving 85 adolescents aged 12–16 years (M = 14.04, SD = 1.09), Sela et al. (2020) demonstrated that although objective time spent online did not exhibit a significant correlation with FoMO, the objective duration of engagement with social networking sites did (*r* = 0.28, *p* < 0.05) [150]. Similarly, Hunt et al. (2018), in a study with 143 undergraduate university students, also established a significant association between FoMO and objectively measured social media usage (*r* = 0.20, *p* < 0.05) [131]. The findings from Shoval et al.’s (2020) study involving 40 college students aged 19–30 years (M = 23.0, SD = 2.4), highlight the significance of disparities between objective and subjective measures. In their study, only those identified by the objective measure as checking their smartphone during the night showed significant differences (checked vs not checked) in FoMO experiences, whereas the subjectively measured data did not [151].

Heterogeneity also exists in terms of variables studied as indicators of psychological well-being or a lack thereof. The review shows that studies on FoMO have examined a diverse range of indicators of well-being (e.g. life satisfaction, positive affect, e.g., [121]), as well as a range of indicators of ill-being (e.g. depression, negative affect) (e.g., [140]). Among all pathways, ill-being indicators have received significantly more attention in the literature compared to well-being indicators, particularly depression and anxiety (e.g., [126, 152]), which have emerged as the most frequently studied aspects in the context of FoMO research. Only on Path 6, the mediated path of digital technology psychological well-being via FoMO, the ill- and well-being indicators have been studied more proportionately.

Moreover, many studies lump together different psychological well-being indicators (ill-being as well as well-being indicators) to draw general conclusions about the impact of FoMO on individuals psychological well-being and vice versa. For instance, Akyol et al. (2021) combine measures of depression, anxiety, and stress under the umbrella term "mental health" in their examination of its impact on FoMO [153], while Wegmann et al. (2017) aggregate measures of depression and interpersonal sensitivity to assess the construct of “psychopathological symptoms” [68].

The inconsistencies in the operationalization and assessment of digital technology use and psychological well-being make it challenging to synthesize the results of the studies and draw conclusions about the relationships between FoMO, digital technology use and psychological well-being, as the effects may vary depending on the digital technology use and psychological well-being indicator studied. This challenge becomes evident in the studies of Yin et al. (2019) [102] and Błachnio and Przepiórka (2018) [108], both of which used the Facebook Intrusion Questionnaire [154], but Yin et al. aimed to measure SNS addiction, whereas Błachnio and Przepiórka aimed to measure Facebook intrusion. Researchers have coined the term "jingle-jangle problem" to elucidate the potential confusion and ambiguity arising when similar terms are used to describe different phenomena [155]. This phenomenon is exemplified in instances such as the terms "self-esteem" and "self-confidence", which some researchers may use interchangeably, assuming their equivalence, while others may assert nuanced disparities between these constructs. Similarly, some scholars use “social media use” to describe a wide range of experiences, from active engagement with close friends on platforms like SnapChat (e.g., [107]), to passive scrolling through captivating Twitter threads or Instagram photos (e.g., [13]). Likewise, the terms “online social networking” may refer to the identical phenomena, hindering knowledge accumulation in the field. The diversity in measures and approaches to measure psychological well-being and digital technology use in relation to FoMO presents a significant challenge. Inconsistencies in operationalization, limited use of objective measures, and varying emphasis on well-being and ill-being indicators make it challenging to synthesize findings and advance research in this field.

## Discussion and research agenda

This study conducted a scoping review of the existing literature on FoMO, which has been characterized as fragmented and lacking a unified understanding of its nature, causes, and consequences. Despite a surge in research since its initial conceptualization by Przybylski and colleagues in 2013, a disjointed understanding of FoMO persists. To address these issues, we systematically analyzed the literature since Przybylski et al.’s seminal work, focusing on two key research questions to clarify how FoMO is conceptualized and uncover its roles in psychological well-being and digital technology use. The first research question aimed to gain more insight into how FoMO has been conceptualized in the literature, and how this conceptualization has varied across the extensive body of research published (RQ1). The second research question aimed to investigate the roles attributed to FoMO in relation to psychological well-being and digital technology use and to assess the extent to which findings supported these roles and the variations in findings across studies (RQ2).

By pursuing these research questions, we aimed to offer clarity and insight into the diverse perspectives on FoMO’s definition, determinants, and consequences, as well as its multifaceted roles in shaping individuals’ psychological well-being and digital technology use. This endeavor was driven by the necessity to bridge gaps in the fragmented FoMO literature and to establish a robust foundation for future research in the digital age.

### Moving towards conceptual clarity and parsimony (RQ1)

Our literature review shows that FoMO is universally acknowledged as an unpleasant feeling stemming from a missed opportunity to engage in an experience, often described as apprehension [65]. Scholars widely concur that these missed experiences fundamentally represent social phenomena rooted in human interaction (e.g., [7, 67]). FoMO revolves around the idea that one is not engaged in an experience others are engaged in. Unpleasant feelings arising from this comparison drive a desire to stay continuously connected with ongoing social experiences. This desire is not confined to the physical aspect of presence. It manifests as a need to engage with what others are doing or discussing, transcending the boundaries of physical participation (e.g., [112]). Nor is FoMO confined to the context of social media [66]. Research shows that people may experience FoMO irrespective of the manner in which missed experiences are brought to their attention. Yet, the literature does show that social media may amplify people’s awareness of missed opportunities [66].

Hence, our literature review suggests that scholars widely agree that FoMO can be seen as (1) an unpleasant feeling over (2) missed social experiences (3) revolving around a comparison of one’s own activities with possible alternative activities, (4) that gives rise to a desire to stay connected with what others are involved in. Yet, the literature review also identifies four key debates concerning the conceptualization of FoMO, which present valuable pathways for future research.

#### Examine the range of feelings that can be experienced in relation to FoMO

Although scholars agree that FoMO involves unpleasant feelings that people experience about unattended experiences, there is still debate about the range of feelings that people encounter when they experience FoMO. While some scholars conceptualize FoMO narrowly as feelings of pervasive apprehension, others are in favor of a broader conceptualization that includes other feelings as well. Hayran et al.’s (2020) proposal to rename FoMO to “Feeling of Missing Out” instead of “Fear of Missing Out” and expand the definition to “the negative affective state that individuals encounter […]”, illustrates this line of thinking [104]. However, this broadening of the conceptual scope may lead to conceptual overlap, making it increasingly difficult to distinguish FoMO from other concepts that have been proposed as antecedents or consequences of FoMO. For example, Przybylski et al. (2013) position negative affect as an antecedent rather than a defining component of FoMO, such that negative affect is more likely to lead to FoMO experiences [5]. With this in mind, it is important to examine the range of affective states fundamental to FoMO in order to determine the conceptual scope of the construct, as well as its boundaries with other constructs such as social comparison and regret tendency (e.g., [156, 157]).

#### Gain insight in the manifestations of FoMO to clarify conceptual boundaries

The literature seems to agree that comparison is central to the FoMO experience. However, there is still ambiguity about what this comparison refers to: (1) one’s own experience with that of someone else (social), and (2) one’s present experience with a more rewarding alternative (competitive), or (3) a combination of both (e.g., [66, 98, 114]). These conceptual boundaries need to be clarified through follow-up research to gain more insight into the situations in which FoMO is likely to manifest. This requires scholars to collect data on the nature (e.g., social, non-social) of one’s current activity and that of the potential alternative activities, as well as the manner in which these alternative activities were brought to one’s attention (e.g., social media). It is also important to explore the extent to which these activities are perceived as rewarding and self-relevant [98], as this will shed light on the psychological processes that drive individuals to constantly seek alternative, potentially more rewarding experiences. Diary research combined with content analysis (what do they see, what are they aware of) can potentially address these questions.

#### Test the conceptual structure of FoMO

The review highlights that there is ambiguity regarding the structure and dimensionality of FoMO. The debate surrounding the conceptualization of FoMO centers on the two key elements of FoMO as proposed by Przybylski et al. (2013): "pervasive apprehension for missed social opportunities" and "a desire to stay continuously connected with what others are doing" [5]. The literature review shows that some scholars emphasize one dimension over the other when defining FoMO, leading to variations in its conceptualization. Moreover, some studies have shortened the original FoMO scale, inadvertently emphasizing either pervasive apprehension or the desire to stay connected component. Despite these variations, only a limited number of studies have tested the dimensionality of FoMO, leaving the psychometric structure of FoMO relatively unexplored. If FoMO indeed does not have a unidimensional structure, but rather consists of two subdimensions, as some research suggests (e.g., [56–88]), this may have implications for the development of a coherent theoretical body of literature on FoMO. As argued by Garrido et al. (2019), approaching FoMO as a unidimensional construct carries the potential risks of "leading to biased item parameter estimates, loss of information, and factor score estimates that cannot be univocally interpreted because they reflect the impact of multiple sources of variance”, when actually it is actually a multidimensional construct [158]. Therefore, future research should examine the conceptual structure of FoMO.

#### Disentangle FoMO as a trait or a state

Research to date lacks evidence to support Przybylski et al.’s perspective that FoMO should be viewed as an individual disposition, i.e., a trait, implying that it remains relatively stable across individuals and over time. Initial research seems to indicate that FoMO can be triggered under certain circumstances at a specific moment in time, and thus can also be considered a state (e.g., [64, 67]). The discussion on whether FoMO should be classified as a trait or a state or perhaps a more complex construct that encompasses both trait and state elements (as suggested by [68]), represents a critical juncture in our understanding of this phenomenon. Future research can validate this by using advanced statistical methods such as Random Intercept Cross-Lagged Panel Model (RI-CLPM), which can disentangle between-person effects (trait-like) from within-person effects (state-like) over time [159]. This approach will provide a more rigorous and nuanced examination of the trait versus state dimension of FoMO, shedding light on its fundamental nature. Ultimately, clarifying the nature of FoMO is crucial to advance our theoretical understanding but also for developing targeted interventions and strategies aimed to mitigate FoMO’s impact on individuals’ psychological well-being and digital technology behaviors.

### Unraveling the multifaceted roles of FoMO in psychological well-being and digital technology use (RQ2)

Our comprehensive review of the literature on the second research question reveals a spectrum of roles attributed to FoMO in relation to psychological well-being and digital technology use, with expected relations from psychological well-being to digital technology use via FoMO and vice versa. Overall, there is ample evidence for a negative relationship between psychological well-being and FoMO, while FoMO is positively associated with technology use. The limited experimental studies that have been conducted, also provide preliminary causal support for an opposing effect; i.e., that restricted technology use increases FoMO (path 4). Hence, it’s important to acknowledge that there is variability in the results of these studies. Nevertheless, the longitudinal evidence strengthens the case for the interrelationship between digital technology use, FoMO, and well-being (path 6), underscoring the important role that FoMO plays in shaping psychological well-being. These findings tentatively indicate that the relationships are bidirectional and that FoMO plays a critical role in these complex interactions. In addition, research indicates that the associations between FoMO and problematic digital technology use are markedly more pronounced than the association between FoMO and conventional usage metrics.

#### Balance research efforts

Since its introduction, research has proposed multiple roles for FoMO in the relationship between psychological well-being and digital technology use. These roles include FoMO as a consequence of well-being (path 1), an antecedent of increased technology use (path 2), as well as a mediator linking the relationship between well-being and technology use (path 3). The literature also hypothesizes reciprocal effects where FoMO functions as a consequence of technology use (path 4), an antecedent of well-being deficits (path 5), and a mediator for the effects of technology use on well-being (path 6). Interestingly, the literature review reveals varying levels of scholarly attention across these paths. While paths 1–3 have received considerable attention, highlighting the initial hypothesis that FoMO motivates technology use due to well-being deficits, as suggested by Przybylski et al. (2013) [5], the underexplored paths 4–6 indicate a need for further investigation. In particular, the limited exploration of path 4, where FoMO arises as a consequence of technology use, calls for more comprehensive research to address societal concerns about technology’s impact on FoMO and psychological well-being [160]. This research would contribute to a more nuanced understanding of the complex interplay between FoMO, digital technology use, and psychological well-being.

Unbalanced is also the disproportionate attention given to young adults in the study of FoMO and its antecedents and consequences. Although there was variation in the study samples, most studies have focused on young adults. Therefore, future research is needed among a more diverse sample to enhance the generalizability of findings and understand FoMO’s impact across different demographics.

*In need of causal research*. Empirical findings seem to provide some support for FoMO’s role as both an antecedent and consequence of psychological well-being and digital technology use. However, the vast majority research in this area has been correlational and cross-sectional in nature, limiting our ability to make causal claims. Experimental studies have provided some insights, supporting the impact of restricted technology use on FoMO (path 4; e.g., [130]). Only one longitudinal study has provided further support for an effect of digital technology use on FoMO (path 4), and through FoMO on stress (path 6; [64]), providing some support for FoMO’s influence on psychological well-being. Yet, these findings relied on two-wave data which can’t fully capture bidirectional dynamics of these relationships. These findings imply that further research should employ experimental and longitudinal designs to better establish causality within these complex relationships. Additionally, exploring the potential cyclic nature of these pathways would be another valuable research endeavor. to put such dynamics to the test, these studies would require scholars to measure FoMO, psychological well-being and technology use at three waves or more [135].

#### In need of theoretical guidance

Most studies eschew the inclusion of theory as an explanatory framework for the presumed relations between FoMO and psychological well-being and/ or digital technology usage. Hence, it is imperative that future research explicitly specify the theoretical lens through which it examines the role of FoMO within the broader nomological network, not only to establish a solid foundation but also to ensure theoretical clarity and empirical validation. The studies that do use theory mostly follow Przybylski et al. who adopted a motivation-based perspective grounded in SDT [161] to explain FoMO and its relationships with psychological well-being and digital technology use. More specifically, they proposed that FoMO arises as a negative emotional state stemming from deficiencies in one or more of these basic psychological needs [5], thereby motivating the desire to stay connected to what others are doing through digital technology use. While scholars have largely embraced Przybylski et al.’s perspective, empirical testing of this theoretical premise has been limited, with only one study reporting moderate associations between psychological need satisfaction and FoMO [137].

Besides SDT [161], a number of other theories have been alluded to explain not only the effects of psychological well-being on FoMO and their downstream consequences for technology use, but also vice versa, such as CIUT) [138] and SCT [144]. Many of these theories are not specific in their explanation of how digital technology use contributes to FoMO and how that negatively affects psychological well-being. This is reflected in our observation that these theories are only mentioned as possible explanations for associations between these variables but are not explicitly tested. As a consequence, these theories are used as bulk theories, that is, as theories that are used to explain the effects of psychological well-being and digital technology use as both consequence and effect of FoMO, when in essence these theories were not developed for this for explaining such bi-directional relationships. Hence, hypotheses formulated in the FoMO literature appear to lack a robust integration with the cited foundational theories, raising concerns about the coherence of the theoretical basis underpinning these hypotheses and hindering our ability to accurately assess the antecedents and consequences of FoMO. Particularly given the potential bidirectional relations between psychological well-being, FoMO, and digital technology use.

One way to advance our theoretical understanding of FoMO is to develop a unifying theory that explains the bi-directionality of psychological well-being, FoMO and digital technology use. The I-PACE model [140] attempts to elucidate these dynamics, but due to conceptual ambiguities, there is no consensus regarding the specific role FoMO plays in this model. Developing a unifying theory would address the concerns raised by various scholars regarding the fragility and fragmentation of the theoretical foundations within the current FoMO literature (e.g., [6]).

#### Unifying terminology in FoMO research

The current state of the literature reveals a heterogeneous landscape in how researchers conceptualize, operationalize and assess psychological well-being and digital technology use, presenting barriers to synthesizing findings and advancing research on FoMO. Factors contributing to this lack of coherence include the uneven distribution of attention to indicators of digital technology use across different pathways. For instance, research on the impact of digital technology use on FoMO (path 4) and its subsequent influence on psychological well-being (path 6) tends to focus more on social media use, while the reverse pathway, examining the effect of psychological well-being on digital technology use via FoMO (path 3), is more commonly explored concerning smartphone use, impeding a holistic understanding.

Additionally, variability in operationalizing digital technology behaviors further complicates the issue, as some studies concentrate on smartphone addiction (e.g., [26]), others explore motives for social media use [27], or internet and online communication use (e.g., [14]), making comparison and generalizations challenging. Differences in time frames used for data collection further exacerbate the problem, with research spanning short-term cross-sectional surveys (e.g., [77]) to longitudinal studies spanning months (e.g., [64]). Moreover, the reliance on self-report measures in lieu of objective data, such as device logs, introduces subjectivity and potential reporting biases. Adding to this complexity is the common practice of individuals accessing social media through their smartphones (e.g., [3, 148]), which makes it hard to distinguish smartphone use from social media use. Understanding these intricacies and relationships will require a more integrated approach to research.

In addition, the literature also reveals a noticeable imbalance in the exploration of indicators of psychological well-being, with a greater emphasis on indicators of ill-being than on indicators of well-being. In the absence of standardized terminology and uniform measures, researchers may use overlapping or interchangeable terms to describe phenomena, which can lead to confusion and hinder cumulative knowledge development. The variation in conceptualizations, measurement methods and approaches to assess the connection between FoMO and psychological well-being poses a substantial obstacle. Incoherent operationalization, restricted use of objective metrics, and differences in the emphasis on indicators of well-being and ill-being create difficulties in consolidating results and pushing forward research in this domain.

To address these challenges and support future research, scholars must agree on a common lexicon that covers the full range of relevant variables. Standardized terms and adopting objective measures, will facilitate cross-study comparison and promote clarity enhancing our understanding of the intricate relationships between FoMO, psychological well-being, and digital technology use.

## Supporting information

S1 Checklist PRISMA-ScR checklist. (PDF)

S1 Text Full search strategies. (PDF)

S1 Table Characteristics of included articles. (PDF)
