# Supplementary material for: The relationship between fear of missing out, digital technology use, and psychological well-being: A scoping review of conceptual and empirical issues
Source: PLoS One. 2024 Oct 4;19(10):e0308643. doi: 10.1371/journal.pone.0308643 (PMC11452005; doi:10.1371/journal.pone.0308643)
Supplement: S1 Text — (PDF) [file pone.0308643.s002.pdf]

S2 Full Search Strategies

Search strategy for PubMed (7 July 2023)

Search	Query	Items found
#5	(#1 OR (#2 AND #3 AND #4))	1848
#4	"Anxiety"[Mesh] OR "Fear"[Mesh] OR "Stress, Psychological"[Mesh] OR "Happiness"[Mesh] OR "Depression"[Mesh] OR "Depressive Disorder"[Mesh] OR "Behavior, Addictive"[Mesh] OR "Compulsive Behavior"[Mesh] OR anxiet*[tiab] OR fear*[tiab] OR stress*[tiab] OR happiness[tiab] OR happy[tiab] OR problem use*[tiab] OR problematic use*[tiab] OR well-being*[tiab] OR wellbeing*[tiab] OR depressi*[tiab] OR life satisfaction*[tiab] OR addicti*[tiab] OR compensatory use*[tiab] OR compulsi*[tiab] OR excessive use*[tiab] OR panic*[tiab] OR self esteem*[tiab] OR loneliness[tiab] OR lonely[tiab] OR perceived social support*[tiab]	2014838
#3	"Cell Phone"[Mesh] OR "Cell Phone Use"[Mesh] OR "Computers, handheld"[Mesh] OR "Mobile Applications"[Mesh] OR "Internet"[Mesh] OR cyberspace[tiab] OR cyber Space[tiab] OR app[tiab] OR apps[tiab] OR smartphone*[tiab] OR smart phone*[tiab] OR phone application*[tiab] OR telephone application*[tiab] OR mobile application*[tiab] OR mobile technolog*[tiab] OR mobile phone*[tiab] OR mobile device*[tiab] OR digital technol*[tiab] OR internet*[tiab] OR world wide web*[tiab] OR ipad[tiab] OR ipads[tiab] OR text messag*[tiab] OR laptop*[tiab] OR iphone*[tiab] OR android[tiab] OR game*[tiab] OR gaming[tiab] OR gamification[tiab] OR whatsapp*[tiab] OR wearable*[tiab] OR social media[tiab] OR "online social network"[tiab] OR "online social networks"[tiab] OR facebook[tiab] OR tiktok[tiab] OR instagram[tiab] OR youtube[tiab] OR snapchat[tiab] OR linkedin[tiab] OR twitter[tiab]	351693
#2	"Social Distance"[Mesh] OR need for belonging[tiab] OR need to belong[tiab] OR social relatedness*[tiab] OR social exclusion*[tiab] OR self determination theor*[tiab] OR social inclusion*[tiab] OR belong*[tiab] OR social rejection*[tiab] OR social acceptance*[tiab] OR social distanc*[tiab] OR attachment*[tiab] OR connectedness*[tiab] OR “connect to others”[tiab] OR “connected to others”[tiab]	411115
#1	“fear of missing out”[tiab] OR fomo[tiab]	249

Search strategy for Ebsco/PsycINFO (7 July 2023)

#	Query	Results
S5	S1 OR (S2 AND S3 AND S4)	2,177
S4	DE "Social Anxiety" OR DE "Fear" OR DE "Stress" OR DE "Psychological Stress" OR DE "Social Stress" OR DE "Stress Reactions" OR DE "Anxiety" OR DE "Depression Emotion" OR DE "Distress" OR DE "Happiness" OR DE "Loneliness" OR DE "Pleasure" OR DE "Positive Emotions" OR DE "Addiction" OR DE "Internet Addiction" OR DE "Quality of Life" OR DE "Well Being" OR DE "Life Satisfaction" OR DE "Panic" OR DE "Self-Esteem" OR DE "Social Support" OR TI (anxiet* OR fear* OR stress* OR happiness OR happy OR “problem use*” OR “problematic use*” OR “well-being*” OR wellbeing* OR depressi* OR “life satisfaction*” OR addicti* OR “compensatory use*” OR compulsi* OR “excessive use*” OR panic* OR “self esteem*” OR loneliness OR lonely OR “perceived social support*”) OR AB (anxiet* OR fear* OR stress* OR happiness OR happy OR “problem use*” OR “problematic use*” OR “well-being*” OR wellbeing* OR depressi* OR “life satisfaction*” OR addicti* OR “compensatory use*” OR compulsi* OR “excessive use*” OR panic* OR “self esteem*” OR loneliness OR lonely OR “perceived social support*”) OR KW (anxiet* OR fear* OR stress* OR	1,089,122

	happiness OR happy OR “problem use*” OR “problematic use*” OR “well-being*” OR wellbeing* OR depressi* OR “life satisfaction*” OR addicti* OR “compensatory use*” OR compulsi* OR “excessive use*” OR panic* OR “self esteem*” OR loneliness OR lonely OR “perceived social support*”)	
**S3**	DE "Internet" DE "Computer Mediated Communication" OR DE "Telecommunications Media" OR DE "Cellular Phones" OR DE "Computer Applications" OR DE "Multimedia" OR DE "Microcomputers" OR DE "Mobile Devices" OR DE "Social Media" OR DE "Online Social Networks" OR DE "Text Messaging" OR DE "Digital Gaming" OR DE "Internet" OR DE "Internet Usage" OR DE "Screen Time" OR DE "Smartphone Use" OR DE "Mobile Applications" OR TI (cyberspace OR “cyber space” OR app OR apps OR smartphone* OR “smart phone*” OR “phone application*” OR “telephone application*” OR “mobile application*” OR “mobile technolog*” OR “mobile phone*” OR “mobile device*” OR “digital technol*” OR internet* OR “world wide web*” OR ipad OR ipads OR “text messag*” OR laptop* OR iphone* OR android OR game* OR gaming OR gamification OR whatsapp* OR wearable* OR “social media” OR "online social network" OR "online social networks" OR facebook OR tiktok OR instagram OR youtube OR snapchat OR linkedin OR twitter) OR AB (cyberspace OR “cyber space” OR app OR apps OR smartphone* OR “smart phone*” OR “phone application*” OR “telephone application*” OR “mobile application*” OR “mobile technolog*” OR “mobile phone*” OR “mobile device*” OR “digital technol*” OR internet* OR “world wide web*” OR ipad OR ipads OR “text messag*” OR laptop* OR iphone* OR android OR game* OR gaming OR gamification OR whatsapp* OR wearable* OR “social media” OR "online social network" OR "online social networks" OR facebook OR tiktok OR instagram OR youtube OR snapchat OR linkedin OR twitter) OR KW (cyberspace OR “cyber space” OR app OR apps OR smartphone* OR “smart phone*” OR “phone application*” OR “telephone application*” OR “mobile application*” OR “mobile technolog*” OR “mobile phone*” OR “mobile device*” OR “digital technol*” OR internet* OR “world wide web*” OR ipad OR ipads OR “text messag*” OR laptop* OR iphone* OR android OR game* OR gaming OR gamification OR whatsapp* OR wearable* OR “social media” OR "online social network" OR "online social networks" OR facebook OR tiktok OR instagram OR youtube OR snapchat OR linkedin OR twitter)	**185,832**
**S2**	DE "Social Acceptance" OR DE "Belonging" OR DE "Psychological Needs" OR TI (“need for belonging” OR “need to belong” OR “social relatedness*” OR “social exclusion*” OR “self determination theor*” OR “social inclusion*” OR belong* OR “social rejection*” OR “social acceptance*” OR “social distanc*” OR attachment* OR connectedness* OR “connect to others” OR “connected to others”) OR AB (“need for belonging” OR “need to belong” OR “social relatedness*” OR “social exclusion*” OR “self determination theor*” OR “social inclusion*” OR belong* OR “social rejection*” OR “social acceptance*” OR “social distanc*” OR attachment* OR connectedness* OR “connect to others” OR “connected to others”) OR KW (“need for belonging” OR “need to belong” OR “social relatedness*” OR “social exclusion*” OR “self determination theor*” OR “social inclusion*” OR belong* OR “social rejection*” OR “social acceptance*” OR “social distanc*” OR attachment* OR connectedness* OR “connect to others” OR “connected to others”)	**123,126**
**S1**	TI (“fear of missing out” OR fomo) OR AB (“fear of missing out” OR fomo) OR KW (“fear of missing out” OR fomo)	**293**

Search strategy for Clarivate Analytics/Web of Science Core Collection (7 July 2023)

**6**	#5 OR #1	**4,275**
**5**	#2 AND #3 AND #4	**3,773**
**4**	TS=("anxiet*" OR "fear*" OR "stress*" OR "happiness" OR "happy" OR "problem use*" OR "problematic use*" OR "well-being*" OR "wellbeing*" OR "depressi*" OR "life satisfaction*" OR "addicti*" OR "compensatory use*" OR "compulsi*" OR "excessive use*" OR "panic*" OR "self esteem*" OR "loneliness" OR "lonely" OR "perceived social support*")	**3,617,024**

**3**	TS=("cyberspace" OR "cyber space" OR "app" OR "apps" OR "smartphone*" OR "smart phone*" OR "phone application*" OR "telephone application*" OR "mobile application*" OR "mobile technolog*" OR "mobile phone*" OR "mobile device*" OR "digital technol*" OR "internet*" OR "world wide web*" OR "ipad" OR "ipads" OR "text messag*" OR "laptop*" OR "iphone*" OR "android" OR "game*" OR "gaming" OR "gamification" OR "whatsapp*" OR "wearable*" OR "social media" OR "online social network" OR "online social networks" OR "facebook" OR "tiktok" OR "instagram" OR "youtube" OR "snapchat" OR "linkedin" OR "twitter")	**876,552**
**2**	TS=("need for belonging" OR "need to belong" OR "social relatedness*" OR "social exclusion*" OR "self determination theor*" OR "social inclusion*" OR "belong*" OR "social rejection*" OR "social acceptance*" OR "social distanc*" OR "attachment*" OR "connectedness*" OR "connect to others" OR "connected to others")	**749,442**
**1**	TS=("fear of missing out" OR "fomo")	**591**
