# Supplementary material for: The relationship between fear of missing out, digital technology use, and psychological well-being: A scoping review of conceptual and empirical issues
Source: PLoS One. 2024 Oct 4;19(10):e0308643. doi: 10.1371/journal.pone.0308643 (PMC11452005; doi:10.1371/journal.pone.0308643)
Supplement: S1 Table — (PDF) [file pone.0308643.s003.pdf]

S3 Characteristics of included articles

#Ref	Study	Measures	Hypothesized model	IV	DV	M	r	*p*	Indirect effects (β/ b [CI], SE, *p*)	Study design	Sample size	Participant characteristics	Theory
90	Aygar et al., 2019	*FoMO:*<br>Fear of Missing Out Scale (FoMOs; Przybylski et al., 2013)<br><br>*Problematic internet use (PIU):*<br>Problematic Internet Use Scale (PIUS; Ceyhan et al., 2007)	Path 4	PIU	FoMO		.42	***		Correlational	*n* = 463	University students aged 18-24y<br>*M* = 21.01 (*SD* = 1.7)	n/a
115	Akyol et al., 2021	*FoMO:*<br>Fear of Missing Out Scale (FoMOs; Przybylski et al., 2013)<br><br>*Problematic mobile phone use (PMPU):*<br>Mobile Phone Problem Use Scale (MPPUS-10; Foerster et al. 2015)<br><br>*Worry:*<br>Penn State Worry Questionnaire-Abbreviated (PSWQ-A; Hopko et al., 2003)	Path 3	Worry	PMPU	FoMO			0.07 [-0.009, 0.080]	Correlational	*n* = 235	Undergraduate students aged 18-25y<br>*M* = 21.72 (*SD* = 2.73)	n/a
115	Akyol et al., 2021	*FoMO:*<br>Fear of Missing Out Scale (FoMOs; Przybylski et al., 2013)<br><br>*Mental health (sum of anxiety, depression, and stress):*<br>Depression Anxiety Stress Scale-21 (DASS-21; Lovibond & Lovibond, 1995)	Path 1	Mental health	FoMO		.36	***		Correlational	*n* = 235	Undergraduate students aged 18-25y<br>*M* = 21.72 (*SD* = 2.73)	n/a
115	Akyol et al., 2021	*FoMO:*<br>Fear of Missing Out Scale (FoMOs; Przybylski et al., 2013)<br><br>*Problematic mobile phone use (PMPU)* :<br>Mobile Phone Problem Use Scale (MPPUS-10; Foerster et al. 2015)<br><br>*Depression:*<br>Depression Anxiety Stress Scale-21 (DASS-21; Lovibond & Lovibond, 1995)	Path 1	Depression	PMPU		.32	***		Correlational	*n* = 235	Undergraduate students aged 18-25y<br>*M* = 21.72 (*SD* = 2.73)	n/a
115	Akyol et al., 2021	*FoMO:*<br>Fear of Missing Out Scale (FoMOs; Przybylski et al., 2013)<br><br>*Anxiety:*<br>Depression Anxiety Stress Scale-21 (DASS-21; Lovibond & Lovibond, 1995)	Path 1	Anxiety	FoMO		.35	***		Correlational	*n* = 235	Undergraduate students aged 18-25y<br>*M* = 21.72 (*SD* = 2.73)	n/a
115	Akyol et al., 2021	*FoMO:*<br>Fear of Missing Out Scale (FoMOs; Przybylski et al., 2013)<br><br>*Stress:*<br>Depression Anxiety Stress Scale-21 (DASS-21; Lovibond & Lovibond, 1995)	Path 1	Stress	FoMO		.31	***		Correlational	*n* = 235	Undergraduate students aged 18-25y<br>*M* = 21.72 (*SD* = 2.73)	n/a
115	Akyol et al., 2021	*FoMO:*<br>Fear of Missing Out Scale (FoMOs; Przybylski et al., 2013)<br><br>*Problematic mobile phone use (PMPU):*<br>Mobile Phone Problem Use Scale (MPPUS-10; Foerster et al. 2015)	Path 2	FoMO	PMPU		.45	***		Correlational	*n* = 235	Undergraduate students aged 18-25y<br>*M* = 21.72 (*SD* = 2.73)	n/a
115	Akyol et al., 2021	*FoMO:*<br>Fear of Missing Out Scale (FoMOs; Przybylski et al., 2013)<br><br>*Problematic mobile phone use (PMPU):*<br>Mobile Phone Problem Use Scale (MPPUS-10; Foerster et al. 2015)<br><br>*Mental health (sum of anxiety, depression, and stress):*<br>Depression Anxiety Stress Scale-21 (DASS-21; Lovibond & Lovibond, 1995)	Path 3	Mental health	PMPU	FoMO			0.03 [-0.002, 0.022]	Correlational	*n* = 235	Undergraduate students aged 18-25y<br>*M* = 21.72 (*SD* = 2.73)	n/a

#Ref	Study	Measures	Hypothesized model	IV	DV	M	r	*p*	Indirect effects (β/ b [CI], SE, *p*)	Study design	Sample size	Participant characteristics	Theory
126	Al-Jallad & Radwan, 2021	*FoMO:*<br>Self-designed 3-item questionnaire: 1) "I feel that I want to stay connected to social media", 2) "I feel anxious when I know of an important event or information too late", 3) "I make sure to use any new social media because of the fear of missing out"<br><br>*Social media fatigue (SMF):*<br>Social media fatigue (Xiao & Mou, 2019)	Path 2	FoMO	SMF		.78	***		Correlational	*n* = 350	Social media users aged 18-35y	CLT
9	Alt & Boniel-Nissim, 2018	*FoMO:*<br>Fear of Missing Out Scale (FoMOs; Przybylski et al., 2013)<br><br>*Problematic internet use (PIU):*<br>Short Problematic Internet Use Test (SPIUT; Siciliano et al., 2015)	Path 2	FoMO	PIU		.53	***		Correlational	*n* = 270	Students enrolled at integrative public secondary or high school 45.6% 13-15y, 54.5% 15.5-18y	n/a
10	Alt & Boniel-Nissim, 2018	*FoMO:*<br>Fear of Missing Out Scale (FoMOs; Przybylski et al., 2013)<br><br>*Problematic internet use (PIU):*<br>Short Problematic Internet Use Test (SPIUT; Siciliano et al., 2015)	Path 2	FoMO	PIU		.38	***		Correlational	*n* = 359	Adults<br>*M* = 48.38 (*SD* = 5.36)	n/a
11	Alt & Boniel-Nissim, 2018	*FoMO:*<br>Fear of Missing Out Scale (FoMOs; Przybylski et al., 2013)<br><br>*Problematic internet use (PIU):*<br>Short Problematic Internet Use Test (SPIUT; Siciliano et al., 2015)	Path 2	FoMO	PIU		.53	***		Correlational	*n* = 216	Students enrolled at integrative public secondary or high school aged 13-18	n/a
42	Alt, 2015	*FoMO:*<br>Fear of Missing Out Scale (FoMOs; Przybylski et al., 2013) (referred to as social FoMO), and 8 additional items (4 news-FoMO + 4 commercial information FoMO) (Alt, 2015)<br><br>*Social media engagement (SME):*<br>Social Media Engagement Questionnaire (Alt, 2015)	Path 2	News-FoMO	SME - Social engagement		.14	*		Correlational	*n* = 296	Undergraduate college students<br>*M* = 25.4 (*SD* = 7.1)	SDT
42	Alt, 2015	*FoMO:*<br>Fear of Missing Out Scale (FoMOs; Przybylski et al., 2013) (referred to as social FoMO), and 8 additional items (4 news-FoMO + 4 commercial information FoMO) (Alt, 2015)<br><br>*Social media engagement (SME):*<br>Social Media Engagement Questionnaire (Alt, 2015)	Path 2	Social-FoMO	SME - News information engagement		.22	**		Correlational	*n* = 296	Undergraduate college students<br>*M* = 25.4 (*SD* = 7.1)	SDT
42	Alt, 2015	*FoMO:*<br>Fear of Missing Out Scale (FoMOs; Przybylski et al., 2013) (referred to as social FoMO), and 8 additional items (4 news-FoMO + 4 commercial information FoMO) (Alt, 2015)<br><br>*Social media engagement (SME):*<br>Social Media Engagement Questionnaire (Alt, 2015)	Path 2	Social-FoMO	SME - Commercial info engagement		.28	**		Correlational	*n* = 296	Undergraduate college students<br>*M* = 25.4 (*SD* = 7.1)	SDT
42	Alt, 2015	*FoMO:*<br>Fear of Missing Out Scale (FoMOs; Przybylski et al., 2013) (referred to as social FoMO), and 8 additional items (4 news-FoMO + 4 commercial information FoMO) (Alt, 2015)<br><br>*Social media engagement (SME):*<br>Social Media Engagement Questionnaire (Alt, 2015)	Path 2	Commercial-FoMO	SME - Social engagement		.32	**		Correlational	*n* = 296	Undergraduate college students<br>*M* = 25.4 (*SD* = 7.1)	SDT
42	Alt, 2015	*FoMO:*<br>Fear of Missing Out Scale (FoMOs; Przybylski et al., 2013) (referred to as social FoMO), and 8 additional items (4 news-FoMO + 4 commercial information FoMO) (Alt, 2015)<br><br>*Social media engagement (SME):*<br>Social Media Engagement Questionnaire (Alt, 2015)	Path 2	News-FoMO	SME - Commercial info engagement		.32	**		Correlational	*n* = 296	Undergraduate college students<br>*M* = 25.4 (*SD* = 7.1)	SDT

#Ref	Study	Measures	Hypothesized model	IV	DV	M	r	*p*	Indirect effects (β/ b [CI], SE, *p*)	Study design	Sample size	Participant characteristics	Theory
42	Alt, 2015	*FoMO:*<br>Fear of Missing Out Scale (FoMOs; Przybylski et al., 2013) (referred to as social FoMO), and 8 additional items (4 news-FoMO + 4 commercial information FoMO) (Alt, 2015)<br><br>*Social media engagement (SME):*<br>Social Media Engagement Questionnaire (Alt, 2015)	Path 2	Social-FoMO	SME - Social engagement		.33	**		Correlational	*n* = 296	Undergraduate college students<br>*M* = 25.4 (*SD* = 7.1)	SDT
42	Alt, 2015	*FoMO:*<br>Fear of Missing Out Scale (FoMOs; Przybylski et al., 2013) (referred to as social FoMO), and 8 additional items (4 news-FoMO + 4 commercial information FoMO) (Alt, 2015)<br><br>*Social media engagement (SME):*<br>Social Media Engagement Questionnaire (Alt, 2015)	Path 2	News-FoMO	SME - News information engagement		.36	**		Correlational	*n* = 296	Undergraduate college students<br>*M* = 25.4 (*SD* = 7.1)	SDT
42	Alt, 2015	*FoMO:*<br>Fear of Missing Out Scale (FoMOs; Przybylski et al., 2013) (referred to as social FoMO), and 8 additional items (4 news-FoMO + 4 commercial information FoMO) (Alt, 2015)<br><br>*Social media engagement (SME):*<br>Social Media Engagement Questionnaire (Alt, 2015)	Path 2	Commercial-FoMO	SME - News information engagement		.52	**		Correlational	*n* = 296	Undergraduate college students<br>*M* = 25.4 (*SD* = 7.1)	SDT
42	Alt, 2015	*FoMO:*<br>Fear of Missing Out Scale (FoMOs; Przybylski et al., 2013) (referred to as social FoMO), and 8 additional items (4 news-FoMO + 4 commercial information FoMO) (Alt, 2015)<br><br>*Social media engagement (SME):*<br>Social Media Engagement Questionnaire (Alt, 2015)	Path 2	Commercial-FoMO	SME - Commercial info engagement		.52	**		Correlational	*n* = 296	Undergraduate college students<br>*M* = 25.4 (*SD* = 7.1)	SDT
43	Alt, 2017	*FoMO:*<br>Fear of Missing Out Scale (FoMOs; Przybylski et al., 2013) (referred to as social FoMO), and 8 additional items (4 news-FoMO + 4 commercial information FoMO) (Alt, 2015)<br><br>*Social media engagement (SME):*<br>Social Media Engagement Questionnaire (Alt, 2015)	Path 2	FoMO	SME		.56	***		Correlational	*n* = 279	Undergraduate college students<br>*M* = 24.3 (*SD* = 4.27)	n/a
32	Alt, 2018	*FoMO:*<br>Fear of Missing Out Scale (FoMOs; Przybylski et al., 2013) (referred to as social FoMO), and 8 additional items (4 news-FoMO + 4 commercial information FoMO) (Alt, 2015)<br><br>*Social media engagement (SME):*<br>Social Media Engagement Questionnaire (Alt, 2015)	Path 2	News-FoMO	SME - Social engagement		.16	**		Correlational	*n* = 290	Undergraduate Social-Science students<br>*M* = 24.1 (*SD* = 6.5)	SDT
32	Alt, 2018	*FoMO:*<br>Fear of Missing Out Scale (FoMOs; Przybylski et al., 2013) (referred to as social FoMO), and 8 additional items (4 news-FoMO + 4 commercial information FoMO) (Alt, 2015)<br><br>*Social media engagement (SME):*<br>Social Media Engagement Questionnaire (Alt, 2015)	Path 2	Social-FoMO	SME - News information engagement		.22	**		Correlational	*n* = 290	Undergraduate Social-Science students<br>*M* = 24.1 (*SD* = 6.5)	SDT
32	Alt, 2018	*FoMO:*<br>Fear of Missing Out Scale (FoMOs; Przybylski et al., 2013) (referred to as social FoMO), and 8 additional items (4 news-FoMO + 4 commercial information FoMO) (Alt, 2015)<br><br>*Social media engagement (SME):*<br>Social Media Engagement Questionnaire (Alt, 2015)	Path 2	Commercial-FoMO	SME - Social engagement		.23	**		Correlational	*n* = 290	Undergraduate Social-Science students<br>*M* = 24.1 (*SD* = 6.5)	SDT
32	Alt, 2018	*FoMO:*<br>Fear of Missing Out Scale (FoMOs; Przybylski et al., 2013) (referred to as social FoMO), and 8 additional items (4 news-FoMO + 4 commercial information FoMO) (Alt, 2015)<br><br>*Social media engagement (SME):*<br>Social Media Engagement Questionnaire (Alt, 2015)	Path 2	Social-FoMO	SME - Commercial info engagement		.28	**		Correlational	*n* = 290	Undergraduate Social-Science students<br>*M* = 24.1 (*SD* = 6.5)	SDT

#Ref	Study	Measures	Hypothesized model	IV	DV	M	r	*p*	Indirect effects (β/ b [CI], SE, *p*)	Study design	Sample size	Participant characteristics	Theory
32	Alt, 2018	*FoMO:*<br>Fear of Missing Out Scale (FoMOs; Przybylski et al., 2013) (referred to as social FoMO), and 8 additional items (4 news-FoMO + 4 commercial information FoMO) (Alt, 2015)<br><br>*Social media engagement (SME):*<br>Social Media Engagement Questionnaire (Alt, 2015)	Path 2	Social-FoMO	SME - Social engagement		.33	**		Correlational	*n* = 290	Undergraduate Social-Science students<br>*M* = 24.1 (*SD* = 6.5)	SDT
32	Alt, 2018	*FoMO:*<br>Fear of Missing Out Scale (FoMOs; Przybylski et al., 2013) (referred to as social FoMO), and 8 additional items (4 news-FoMO + 4 commercial information FoMO) (Alt, 2015)<br><br>*Social media engagement (SME):*<br>Social Media Engagement Questionnaire (Alt, 2015)	Path 2	News-FoMO	SME - Commercial info engagement		.37	**		Correlational	*n* = 290	Undergraduate Social-Science students<br>*M* = 24.1 (*SD* = 6.5)	SDT
32	Alt, 2018	*FoMO:*<br>Fear of Missing Out Scale (FoMOs; Przybylski et al., 2013) (referred to as social FoMO), and 8 additional items (4 news-FoMO + 4 commercial information FoMO) (Alt, 2015)<br><br>*Social media engagement (SME):*<br>Social Media Engagement Questionnaire (Alt, 2015)	Path 2	News-FoMO	SME - News information engagement		.44	**		Correlational	*n* = 290	Undergraduate Social-Science students<br>*M* = 24.1 (*SD* = 6.5)	SDT
32	Alt, 2018	*FoMO:*<br>Fear of Missing Out Scale (FoMOs; Przybylski et al., 2013) (referred to as social FoMO), and 8 additional items (4 news-FoMO + 4 commercial information FoMO) (Alt, 2015)<br><br>*Social media engagement (SME):*<br>Social Media Engagement Questionnaire (Alt, 2015)	Path 2	Commercial-FoMO	SME - News information engagement		.52	**		Correlational	*n* = 290	Undergraduate Social-Science students<br>*M* = 24.1 (*SD* = 6.5)	SDT
32	Alt, 2018	*FoMO:*<br>Fear of Missing Out Scale (FoMOs; Przybylski et al., 2013) (referred to as social FoMO), and 8 additional items (4 news-FoMO + 4 commercial information FoMO) (Alt, 2015)<br><br>*Social media engagement (SME):*<br>Social Media Engagement Questionnaire (Alt, 2015)	Path 2	Commercial-FoMO	SME - Commercial info engagement		.52	**		Correlational	*n* = 290	Undergraduate Social-Science students<br>*M* = 24.1 (*SD* = 6.5)	SDT
65	Baker et al., 2016	*FoMO:*<br>Fear of Missing Out Scale (FoMOs; Przybylski et al., 2013)<br><br>*Depression:*<br>Center for Epidemiological Studies--Depression Scale (CES-D; Lewinsohn et al., 1997)	Path 5	FoMO	Depression		.36	***		Correlational	*n* = 386	Undergraduate university students<br>*M* = 21.98 (*SD* = 5.22)	n/a
80	Balta et al., 2018	*FoMO:*<br>Trait-State Fear of Missing Out Scale (T-SFoMOSC; Wegmann et al. 2017)<br><br>*Problematic Instagram use:*<br>Social Media Use Questionnaire (SMUQI; Xanidis and Brignell, 2016)	Path 2	Trait-FoMO	Problematic Instagram use		.38	***		Correlational	*n* = 423	Active Instagram users aged 14-21y<br>*M* = 17.15 (*SD* = 2.24)	n/a
80	Balta et al., 2018	*FoMO:*<br>Trait-State Fear of Missing Out Scale (T-SFoMOSC; Wegmann et al. 2017)<br><br>*Problematic Instagram use:*<br>Social Media Use Questionnaire (SMUQI; Xanidis and Brignell, 2016)	Path 2	State-FoMO	Problematic Instagram use		.60	***		Correlational	*n* = 423	Active Instagram users aged 14-21y<br>*M* = 17.15 (*SD* = 2.24)	n/a
1	Barry & Wong, 2020	*FoMO:*<br>9 items (Q1, Q3, Q4, Q5, Q6, Q7, Q8, Q9, Q10) of Fear of Missing Out Scale (FoMOs; Przybylski et al., 2013)<br><br>*Social media use during daily activities:*<br>Self-designed questionnaire asking participants to indicate whether they check social media during certain activities (e.g., during meals, in the 15 min before going to sleep)	Path 4	SMU during daily activities	FoMO		.49	***		Correlational	*n* = 419	Individuals aged 14 -47y	n/a

#Ref	Study	Measures	Hypothesized model	IV	DV	M	r	*p*	Indirect effects (β/ b [CI], SE, *p*)	Study design	Sample size	Participant characteristics	Theory
1	Barry & Wong, 2020	*FoMO:*<br>9 items (Q1, Q3, Q4, Q5, Q6, Q7, Q8, Q9, Q10) of Fear of Missing Out Scale (FoMOs; Przybylski et al., 2013)<br><br>*Loneliness:*<br>UCLA Loneliness Scale-3 (UCLA-3; Russel, 1996)	Path 5	Loneliness	FoMO		.26	***		Correlational	*n* = 419	Individuals aged 14 -47y	n/a
1	Barry & Wong, 2020	*FoMO:*<br>9 items (Q1, Q3, Q4, Q5, Q6, Q7, Q8, Q9, Q10) of Fear of Missing Out Scale (FoMOs; Przybylski et al., 2013)<br><br>*Self-esteem:*<br>Rosenberg Self-Esteem Scale (RSES; Rosenberg, 1965)	Path 5	Self-esteem	FoMO		-.27	***		Correlational	*n* = 419	Individuals aged 14 -47y	n/a
1	Barry & Wong, 2020	*FoMO:*<br>9 items (Q1, Q3, Q4, Q5, Q6, Q7, Q8, Q9, Q10) of Fear of Missing Out Scale (FoMOs; Przybylski et al., 2013)<br><br>*Satisfaction with life (SwL):*<br>Satisfaction With Life Scale (SWLS; Diener et al., 1985)	Path 5	SwL	FoMO		-.08	ns		Correlational	*n* = 419	Individuals aged 14 -47y	n/a
1	Barry & Wong, 2020	*FoMO:*<br>9 items (Q1, Q3, Q4, Q5, Q6, Q7, Q8, Q9, Q10) of Fear of Missing Out Scale (FoMOs; Przybylski et al., 2013)<br><br>*Self-compassion:*<br>Self-compassion Scale (SCS; Neff, 2003b)	Path 5	Self-compassion	FoMO		-.22	***		Correlational	*n* = 419	Individuals aged 14 -47y	n/a
1	Barry & Wong, 2020	*FoMO:*<br>9 items (Q1, Q3, Q4, Q5, Q6, Q7, Q8, Q9, Q10) of Fear of Missing Out Scale (FoMOs; Przybylski et al., 2013)<br><br>*Frequency of checking social media (SM):*<br>self-designed questionnaire asking participants how frequently they check social media	Path 4	Frequency of checking SM	FoMO		.15	**		Correlational	*n* = 419	Individuals aged 14 -47y	n/a
1	Barry & Wong, 2020	*FoMO:*<br>9 items (Q1, Q3, Q4, Q5, Q6, Q7, Q8, Q9, Q10) of Fear of Missing Out Scale (FoMOs; Przybylski et al., 2013)<br><br>*Number of social media (SM) accounts:*<br>Self-designed questionnaire asking participants the number of social media accounts they have	Path 4	Number of SM accounts	FoMO		.34	***		Correlational	*n* = 419	Individuals aged 14 -47y	n/a
127	Barry et al., 2017	*FoMO:*<br>Fear of Missing Out Scale (FoMOs; Przybylski et al., 2013)<br><br>*Frequency of checking social media (SM):*<br>Self-designed questionnaire asking participants o.a. frequency of checking	*No path specified*	FoMO	Frequency of checking SM		.35	***		Correlational	*n* = 113	Adolescents aged 14-17y<br>*M* = 15.27 (*SD* = 1.02)	n/a
127	Barry et al., 2017	*FoMO:*<br>Fear of Missing Out Scale (FoMOs; Przybylski et al., 2013)<br><br>*Number of social media (SM) accounts:*<br>Self-designed questionnaire asking participants o.a. number of social media accounts	*No path specified*	FoMO	# of accounts		.40	***		Correlational	*n* = 113	Adolescents aged 14-17y<br>*M* = 15.27 (*SD* = 1.02)	n/a
15	Beyens et al., 2016	*FoMO:*<br>9 items (Q1 + Q2 combined) of Fear of Missing Out Scale (FoMOs; Przybylski et al., 2013)<br><br>*Perceived stress due to not being popular on Facebook:*<br>Self-designed questionnaire measuring experienced stress due to not being popular on Facebook	Path 5	FoMO	Perceived stress due to not being popular on Facebook		.88	***		Correlational	*n* = 402	High school students grades 9-12<br>*M* = 16.41 (*SD* = 1.43)	n/a
15	Beyens et al., 2016	*FoMO:*<br>9 items (1+2 combined) of Fear of Missing Out Scale (FoMOs; Przybylski et al., 2013)<br><br>*Need for Popularity:*<br>11 items of the Popularity Scale (Santor et al., 2000)	Path 1	Need for popularity	FoMO		.48	***		Correlational	*n* = 402	High school students grades 9-12<br>*M* = 16.41 (*SD* = 1.43)	n/a

#Ref	Study	Measures	Hypothesized model	IV	DV	M	r	*p*	Indirect effects (β/ b [CI], SE, *p*)	Study design	Sample size	Participant characteristics	Theory
15	Beyens et al., 2016	*FoMO:*<br>9 items (Q1 + Q2 combined) of Fear of Missing Out Scale (FoMOs; Przybylski et al., 2013)<br><br>*Facebook use:*<br>The Facebook Intensity Scale (Ellison et al., 2007)	Path 2	FoMO	Facebook use		.50	***		Correlational	*n* = 402	High school students grades 9-12<br>*M* = 16.41 (*SD* = 1.43)	n/a
15	Beyens et al., 2016	*FoMO:*<br>9 items (Q1 + Q2 combined) of Fear of Missing Out Scale (FoMOs; Przybylski et al., 2013)<br><br>*Perceived stress due to not belonging on Facebook:*<br>Self-designed questionnaire measuring experienced stress due to not belonging on Facebook	Path 5	FoMO	Perceived stress due to not belonging on Facebook		.74	***		Correlational	*n* = 402	High school students grades 9-12<br>*M* = 16.41 (*SD* = 1.43)	n/a
15	Beyens et al., 2016	*FoMO:*<br>9-items (Q1 + Q2 combined) of Fear of Missing Out Scale (FoMOs; Przybylski et al., 2013)<br><br>*Need to belong:*<br>Need to Belong Scale (NTBS; Leary et al., 2013)	Path 1	Need to belong	FoMO		.53	***		Correlational	*n* = 402	High school students grades 9-12<br>*M* = 16.41 (*SD* = 1.43)	n/a
70	Błachnio & Przepiórka, 2018	*FoMO:*<br>Fear of Missing Out Scale (FoMOs; Przybylski et al., 2013)<br><br>*Satisfaction with life (SwL):*<br>Satisfaction With Life Scale (SWLS; Diener et al., 1985)	Path 5	FoMO	SwL		.10	ns		Correlational	*n* = 360	Facebook users aged 16-53y<br>*M* = 22.22 (*SD* = 6.84)	n/a
70	Błachnio & Przepiórka, 2018	*FoMO:*<br>Fear of Missing Out Scale (FoMOs; Przybylski et al., 2013)<br><br>*Facebook intrusion:*<br>Facebook Intrusion Questionnaire (Elphinston & Noller, 2011)	Path 2	FoMO	Facebook intrusion		.45	***		Correlational	*n* = 360	Facebook users aged 16-53y<br>*M* = 22.22 (*SD* = 6.84)	n/a
81	Blackwell et al., 2017	*FoMO:*<br>Fear of Missing Out Scale (FoMOs; Przybylski et al., 2013)<br><br>*Social media engagement (SME):*<br>Social Media Engagement Questionnaire (SMEQ ; Przybylski et al., 2013)	Path 2	FoMO	SME		.36	***		Correlational	*n* = 207	Individuals aged 17-49y<br>*M* = 22.15 (*SD* = 7.38)	n/a
81	Blackwell et al., 2017	*FoMO:*<br>Fear of Missing Out Scale (FoMOs; Przybylski et al., 2013)<br><br>*Social media addiction (SMA)* :<br>Bergen Social Media Addiction Scale (BSMAS; Andreassen et al., 2012)	Path 2	FoMO	SMA		.56	***		Correlational	*n* = 207	Individuals aged 17-49y<br>*M* = 22.15 (*SD* = 7.38)	n/a
128	Blanca & Bendayan, 2018	*FoMO:*<br>Fear of Missing Out Scale (FoMOs; Przybylski et al., 2013)<br><br>*Communication disturbance:*<br>Phubbing Scale (PS; Karadağ et al., 2015) (EFA showed two factors: Communication disturbance and Phone Obsession)	Path 2	FoMO	Communication disturbance		.22	**		Correlational	*n* = 759	Mobile phone users aged 18+<br>*M* = 29.07 (*SD* = 12.07)	n/a
128	Blanca & Bendayan, 2018	*FoMO:*<br>Fear of Missing Out Scale (FoMOs; Przybylski et al., 2013)<br><br>*Communication disturbance:*<br>Phubbing Scale (PS; Karadağ et al., 2015) (EFA showed two factors: Communication disturbance and Phone Obsession)	Path 2	FoMO	Phone obsession		.31	**		Correlational	*n* = 759	Mobile phone users aged 18+<br>*M* = 29.07 (*SD* = 12.07)	n/a
129	Bloemen & De Coninck, 2020	*FoMO:*<br>Self-designed questionnaire: 1) "If I am someplace where I can't be online, then I am annoyed by this", 2) "I am afraid I will miss things if I don't use social media", and 3) "I feel restless when I receive a social media message and I can't look at it immediately"<br><br>*Social media use (SMU):*<br>Mean of two variables 1) number of minutes spend on SNS during an average weekday and 2) on a average weekend day	Path 4	SMU	FoMO		.29	**		Correlational	*n* = 821	Secondary school students aged 13-18y	SDT

#Ref	Study	Measures	Hypothesized model	IV	DV	M	r	*p*	Indirect effects (β/ b [CI], SE, *p*)	Study design	Sample size	Participant characteristics	Theory
130	Boustead & Flack, 2021	*FoMO:*<br>Fear of Missing Out Scale (FoMOs; Przybylski et al., 2013)<br><br>*Problematic social networking site (SNS) use:*<br>Compulsive Internet Use Scale (CIUS; Meerker et al., 2009)	Path 2	FoMO	Problematic SNS use		.63	**		Correlational	*n* = 188	Individuals aged 18-72y<br>*M* = 31.95 (*SD* = 11.54)	n/a
34	Brailovskaia et al., 2021	*FoMO:*<br>Fear of Missing Out Scale (FoMOs; Przybylski et al., 2013)<br><br>*Sense of control:*<br>Sense of control (Niemeyer et al., 2019)	Path 1	Sense of control	FoMO		-.30	***		Correlational	*n* = 516	Smartphone users aged 18-79<br>*M* = 31.91 (*SD* = 12.96)	I-PACE
34	Brailovskaia et al., 2021	*FoMO:*<br>Fear of Missing Out Scale (FoMOs; Przybylski et al., 2013)<br><br>*Problematic smartphone use (PSU):*<br>Bergen Social Media Addiction Scale (BSMAS; Andreassen et al., 2016)	Path 2	FoMO	PSU		.53	***		Correlational	*n* = 516	Smartphone users aged 18-79<br>*M* = 31.91 (*SD* = 12.96)	I-PACE
34	Brailovskaia et al., 2021	*FoMO:*<br>Fear of Missing Out Scale (FoMOs; Przybylski et al., 2013)<br><br>*Repetitive negative thinking (RNT):*<br>Perseverative Thinking Questionnaire (PTQ; Ehring et al., 2011)	Path 1	RNT	FoMO		.36	***		Correlational	*n* = 516	Smartphone users aged 18-79<br>*M* = 31.91 (*SD* = 12.96)	I-PACE
34	Brailovskaia et al., 2021	*FoMO:*<br>Fear of Missing Out Scale (FoMOs; Przybylski et al., 2013)<br><br>*Repetitive negative thinking (RNT):*<br>Perseverative Thinking Questionnaire (PTQ; Ehring et al., 2011)	Path 2	FoMO	Smartphone use time		.53	***		Correlational	*n* = 516	Smartphone users aged 18-79<br>*M* = 31.91 (*SD* = 12.96)	I-PACE
91	Brown & Kuss, 2020	*Perceived FoMO pre and post social media (SM) abstinence:*<br>Fear of Missing Out Scale (FoMOs; Przybylski et al., 2013)	Path 4	7-day SM abstinence	FoMO		.53			Causal/ experiment	*n* = 61	Opportunity sample willing to abstain from social media for seven days, aged 20-49y	SDT, UGT
26	Buglass et al., 2017	*FoMO:*<br>Fear of Missing Out Scale (FoMOs; Przybylski et al., 2013)<br><br>*Self-esteem:*<br>Rosenberg Self-Esteem Scale (RSE, 1965)	Path 5	FoMO T1	Self-esteem T2		-.20	**		Two-wave longitudinal study (6 months apart; same sample)	Correlational *n* = 506, longitudinal *n* = 175	Facebook users, range 13-77y<br>*M* = 20.7 (*SD* = 9.10)	n/a
26	Buglass et al., 2017	*FoMO:*<br>Fear of Missing Out Scale (FoMOs; Przybylski et al., 2013)<br><br>*Social networking site (SNS) use:*<br>One item measure to assess an individual's daily use of Facebook	Path 4	SNS use T1	FoMO T2		.37	***		Two-wave longitudinal study (6 months apart; same sample)	Correlational *n* = 506, longitudinal *n* = 175	Facebook users, range 13-77y<br>*M* = 20.7 (*SD* = 9.10)	n/a
26	Buglass et al., 2017	*FoMO:*<br>Fear of Missing Out Scale (FoMOs; Przybylski et al., 2013)<br><br>*Social networking site (SNS) use:*<br>One item measure to assess an individual's daily use of Facebook	Path 4	SNS use	FoMO		.25	***		Two-wave longitudinal study (6 months apart; same sample)	Correlational *n* = 506, longitudinal *n* = 175	Facebook users, range 13-77y<br>*M* = 20.7 (*SD* = 9.10)	n/a
26	Buglass et al., 2017	*FoMO:*<br>Fear of Missing Out Scale (FoMOs; Przybylski et al., 2013)<br><br>*Self-esteem:*<br>Rosenberg Self-Esteem Scale (RSE, 1965)	Path 5	FoMO	Self-esteem		.29	***		Two-wave longitudinal study (6 months apart; same sample)	Correlational *n* = 506, longitudinal *n* = 175	Facebook users, range 13-77y<br>*M* = 20.7 (*SD* = 9.10)	n/a
26	Buglass et al., 2017	*FoMO:*<br>Fear of Missing Out Scale (FoMOs; Przybylski et al., 2013)<br><br>*Self-esteem:*<br>Rosenberg Self-Esteem Scale (RSE, 1965)	Path 5	FoMO T1	Self-esteem T1		-.30	***		Two-wave longitudinal study (6 months apart; same sample)	Correlational *n* = 506, longitudinal *n* = 175	Facebook users, range 13-77y<br>*M* = 20.7 (*SD* = 9.10)	n/a
26	Buglass et al., 2017	*FoMO:*<br>Fear of Missing Out Scale (FoMOs; Przybylski et al., 2013)<br><br>*Social networking site (SNS) use:*<br>One item measure to assess an individual's daily use of Facebook	Path 4	SNS use T1	FoMO T2		.37	***		Two-wave longitudinal study (6 months apart; same sample)	Correlational *n* = 506, longitudinal *n* = 175	Facebook users, range 13-77y<br>*M* = 20.7 (*SD* = 9.10)	n/a
26	Buglass et al., 2017	*FoMO:*<br>Fear of Missing Out Scale (FoMOs; Przybylski et al., 2013)<br><br>*Social networking site (SNS) use:*<br>One item measure to assess an individual's daily use of Facebook	Path 4	SNS use T1	FoMO T1		.43	***		Two-wave longitudinal study (6 months apart; same sample)	Correlational *n* = 506, longitudinal *n* = 175	Facebook users, range 13-77y<br>*M* = 20.7 (*SD* = 9.10)	n/a

#Ref	Study	Measures	Hypothesized model	IV	DV	M	r	*p*	Indirect effects (β/ b [CI], SE, *p*)	Study design	Sample size	Participant characteristics	Theory
26	Buglass et al., 2017	*FoMO:*<br>Fear of Missing Out Scale (FoMOs; Przybylski et al., 2013)<br><br>*Social networking site (SNS) use:*<br>One item measure to assess an individual's daily use of Facebook<br><br>*Self-esteem:*<br>Rosenberg Self-Esteem Scale (RSE, 1965)	Path 6	SNS use	Self-esteem	FoMO			-.03 [-0.04, -0.020]	Two-wave longitudinal study (6 months apart; same sample)	Correlational *n* = 506, longitudinal *n* = 175	Facebook users, range 13-77y<br>*M* = 20.7 (*SD* = 9.10)	n/a
13	Burnell et al., 2019	*FoMO:*<br>Fear of Missing Out Scale (FoMOs; Przybylski et al., 2013)<br><br>*Social networking site (SNS) - passive browsing:*<br>One item: 'How many minutes per day do you spend viewing others 'profiles?'	Path 4	SNS passive browsing	FoMO		.17	***		Correlational	*n* = 744	Undergraduate university students aged 18-59y<br>*M* = 21.47 (*SD* = 4.64)	SCT
13	Burnell et al., 2019	FoMO:<br>Fear of Missing Out Scale (FoMOs; Przybylski et al., 2013)<br><br>*Global self-worth:*<br>Subscale of the Self-perception Profile for College Students (Neeman & Harter, 2012)	Path 5	FoMO	Social acceptance		-.26	***		Correlational	*n* = 744	Undergraduate university students aged 18-59y<br>*M* = 21.47 (*SD* = 4.64)	SCT
13	Burnell et al., 2019	*FoMO:*<br>Fear of Missing Out Scale (FoMOs; Przybylski et al., 2013)<br><br>*Depression:*<br>Patient Health Questionnaire (PHQ-9; Kroenke et al., 2001)	Path 5	FoMO	Depression		.34	***		Correlational	*n* = 744	Undergraduate university students aged 18-59y<br>*M* = 21.47 (*SD* = 4.64)	SCT
13	Burnell et al., 2019	*FoMO:*<br>Fear of Missing Out Scale (FoMOs; Przybylski et al., 2013)<br><br>*Global self-worth:*<br>Subscale of the Self-perception Profile for College Students (Neeman & Harter, 2012)	Path 5	FoMO	Global self-worth		-.36	***		Correlational	*n* = 744	Undergraduate university students aged 18-59y<br>*M* = 21.47 (*SD* = 4.64)	SCT
13	Burnell et al., 2019	*FoMO:*<br>Fear of Missing Out Scale (FoMOs; Przybylski et al., 2013)<br><br>*Social networking site (SNS) passive browsing:*<br>One item: 'How many minutes per day do you spend viewing others 'profiles?'<br><br>*Social comparison orientation:*<br>Subscale social comparison of the Comparison and Feedback-Seeking Scale (Nesi, 2014; Nesi & Prinstein, 2015)<br><br>*Social acceptance:*<br>Subscale of the Self-perception Profile for College Students (Neeman & Harter, 2012)	Path 6	SNS passive browsing	Social acceptance	SCO > FoMO			-.03, $p < .001$	Correlational	*n* = 744	Undergraduate university students aged 18-59y<br>*M* = 21.47 (*SD* = 4.64)	SCT
13	Burnell et al., 2019	*FoMO:*<br>Fear of Missing Out Scale (FoMOs; Przybylski et al., 2013)<br><br>*Social networking site (SNS) passive browsing:*<br>One item: 'How many minutes per day do you spend viewing others' profiles?<br><br>*Social comparison orientation:*<br>Subscale social comparison of the Comparison and Feedback-Seeking Scale (Nesi, 2014; Nesi & Prinstein, 2015)<br><br>*Global self-worth:*<br>*S* ubscale of the Self-perception Profile for College Students (Neeman & Harter, 2012)	Path 6	SNS passive browsing	Global self-worth	SCO > FoMO			-.04, $p < .001$	Correlational	*n* = 744	Undergraduate university students aged 18-59y<br>*M* = 21.47 (*SD* = 4.64)	SCT

#Ref	Study	Measures	Hypothesized model	IV	DV	M	r	*p*	Indirect effects (β/ b [CI], SE, *p*)	Study design	Sample size	Participant characteristics	Theory
13	Burnell et al., 2019	*FoMO:*<br>Fear of Missing Out Scale (FoMOs; Przybylski et al., 2013)<br><br>*Social networking site (SNS) passive browsing:*<br>One item: 'How many minutes per day do you spend viewing others' profiles?<br><br>*Social comparison orientation (SCO):*<br>Subscale social comparison of the Comparison and Feedback-Seeking Scale (Nesi, 2014; Nesi & Prinstein, 2015)<br><br>*Depression:*<br>Patient Health Questionnair (PHQ-9; Kroenke et al., 2001)	Path 6	SNS passive browsing	Depression	SCO > FoMO			-.04, *p* < .001	Correlational	*n* = 744	Undergraduate university students aged 18-59y<br>*M* = 21.47 (*SD* = 4.64)	SCT
12	Busch et al., 2021	*FoMO:*<br>3 items (Q2, Q3, Q10) of Fear of Missing Out Scale (FoMOs; Przybylski et al., 2013)<br><br>*Problematic Smartphone Use (PSU):*<br>Mobile phone problematic use scale (MPPUS-10; Foerster et al., 2015)	Path 2	FoMO	PSU		.39	*		Correlational	*n* = 154	Smartphone users aged 60 years and older	n/a
46	Can & Satici, 2019	*FoMO:*<br>Fear of Missing Out Scale (FoMOs; Przybylski et al., 2013)<br><br>*Life satisfaction (LS):*<br>Satisfaction with Life Scale (SWLS; Diener et al., 1985)	*No path specified*	FoMO	LS		-.21	**		Correlational	*n* = 371	Social media users aged 15-70y<br>*M* = 33.65 (*SD* = 12.33)	n/a
46	Can & Satici, 2019	*FoMO:*<br>Fear of Missing Out Scale (FoMOs; Przybylski et al., 2013)<br><br>*Average time on social media (SM):*<br>One item: average time that they spent on social media as a daily routine	*No path specified*	FoMO	Average time on SM		.24	**		Correlational	*n* = 371	Social media users aged 15-70y<br>*M* = 33.65 (*SD* = 12.33)	n/a
46	Can & Satici, 2019	*FoMO:*<br>Fear of Missing Out Scale (FoMOs; Przybylski et al., 2013)<br><br>*Problematic Facebook use:*<br>Bergen Facebook Addiction Scale (BFAS; Andreassen et al., 2012)	*No path specified*	FoMO	Problematic Facebook use		.43	**		Correlational	*n* = 371	Social media users aged 15-70y<br>*M* = 33.65 (*SD* = 12.33)	n/a
131	Casale et al., 2018	*FoMO:*<br>Fear of Missing Out Scale (FoMOs; Przybylski et al., 2013)<br><br>*Problematic social networking site (SNS) use:*<br>Bergen Social Media Addiction Scale (BSMAS; Monacis et al., 2017)	Path 2	FoMO	Problematic SNSs use (females)		.44	**		Correlational	*n* = 579	Undergraduates enrolled at university<br>*M* = 22.39 (*SD* = 2.82)	UGT
131	Casale et al., 2018	*FoMO:*<br>Fear of Missing Out Scale (FoMOs; Przybylski et al., 2013)<br><br>*Problematic social networking site (SNS) use:*<br>Bergen Social Media Addiction Scale (BSMAS; Monacis et al., 2017)	Path 2	FoMO	Problematic SNSs use (males)		.47	**		Correlational	*n* = 579	Undergraduates enrolled at university<br>*M* = 22.39 (*SD* = 2.82)	UGT
124	Çatiker et al., 2021	*FoMO:*<br>Fear of Missing Out Scale (FoMOs; Przybylski et al., 2013)<br><br>*Smartphone addiction:*<br>Smartphone Addiction Scale (SAS; Kwon et al., 2013)	*No path specified*	FoMO	Smartphone addiction		.33	*		Correlational	*n* = 97	Nursing students who use a smartphone	n/a
132	Chen & Cheung, 2019	*FoMO:*<br>Fear of Missing Out Scale (FoMOs; Przybylski et al., 2013)<br><br>*Engagement with ephemeral content:*<br>Social Media Engagement (SME) Questionnaire (Alt, 2015)	Path 2	FoMO	Engagement with ephemeral content		.14	*		Correlational	*n* = 303	Social media users aged 18-39y	UGT
68	Chotpitayasunondh & Douglas, 2016	*FoMO:*<br>Fear of Missing Out Scale (FoMOs; Przybylski et al., 2013)<br><br>*Smartphone addiction:*<br>Smartphone Addiction Scale - Short Version (SAS-SV; Kwon et al., 2013)	Path 2	FoMO	Smartphone addiction		.58	**		Correlational	*n* = 251	Smartphone users aged 18-66y<br>*M* = 27.70 (SD = 9.59)	n/a

#Ref	Study	Measures	Hypothesized model	IV	DV	M	r	*p*	Indirect effects (β/ b [CI], SE, *p*)	Study design	Sample size	Participant characteristics	Theory
133	Classen et al., 2020	*FoMO:*<br>Fear of Missing Out Scale (FoMOs; Przybylski et al., 2013)<br><br>*Psychological need satisfaction (PNS):*<br>Psychological Need Satisfaction Scale (PNSS; La Guardia et al., 2000)	Path 1	PNS	SME		-.18	**		Correlational	*n* = 218	Social media users aged 18-49y	SDT
133	Classen et al., 2020	*FoMO:*<br>Fear of Missing Out Scale (FoMOs; Przybylski et al., 2013)<br><br>*Social media engagement (SME):*<br>Social Media Engagement Questionnaire (SMEQ; Przybylski et al., 2013)	Path 2	FoMO	SME		.33	**		Correlational	*n* = 218	Social media users aged 18-49y	SDT
133	Classen et al., 2020	*FoMO:*<br>Fear of Missing Out Scale (FoMOs; Przybylski et al., 2013)<br><br>*Social media engagement (SME):*<br>Social Media Engagement Questionnaire (SMEQ; Przybylski et al., 2013)<br><br>*Psychological need satisfaction (PNS):*<br>Psychological Need Satisfaction Scale (PNSS; La Guardia et al., 2000)	Path 3	PNS	SME	FoMO			.06 [-0.12, -0.02]	Correlational	*n* = 218	Social media users aged 18-49y	SDT
67	Çoşkun & Muslu, 2019	*FoMO:*<br>Fear of Missing Out Scale (FoMOs; Przybylski et al., 2013)<br><br>*Problematic phone use (PU) consequences:*<br>Scale for Problematic Mobile Phone Use (PU; Augner & Hacker,	Path 2	FoMO	Problematic PU Consequences		.29	***		Correlational	*n* = 1630	High school students<br>58.7% aged 15-16y, 41.3% aged 17-18y	n/a
67	Çoşkun & Muslu, 2019	*FoMO:*<br>Fear of Missing Out Scale (FoMOs; Przybylski et al., 2013)<br><br>*Problematic phone use social relationships:*<br>Scale for Problematic Mobile Phone Use (PU; Augner & Hacker,	Path 2	FoMO	Problematic PU Social Relationships		.30	***		Correlational	*n* = 1630	High school students<br>58.7% aged 15-16y, 41.3% aged 17-18y	n/a
67	Çoşkun & Muslu, 2019	*FoMO:*<br>Fear of Missing Out Scale (FoMOs; Przybylski et al., 2013)<br><br>*Problematic phone use dependence:*<br>Scale for Problematic Mobile Phone Use (PU; Augner & Hacker,	Path 2	FoMO	Problematic PU Dependence		.36	***		Correlational	*n* = 1630	High school students<br>58.7% aged 15-16y, 41.3% aged 17-18y	n/a
67	Çoşkun & Muslu, 2019	*FoMO:*<br>Fear of Missing Out Scale (FoMOs; Przybylski et al., 2013)<br><br>*Problematic phone use:*<br>Scale for Problematic Mobile Phone Use (PU; Augner & Hacker,	Path 2	FoMO	Problematic PU (total)		.38	***		Correlational	*n* = 1630	High school students<br>58.7% aged 15-16y, 41.3% aged 17-18y	n/a
85	Dempsey et al., 2019	*FoMO:*<br>Fear of Missing Out Scale (FoMOs; Przybylski et al., 2013)<br><br>*Life satisfaction (LS):*<br>The Satisfaction with Life Scale (SWLS; Diener et al., 1985)	Path 1	LS	FoMO		-.11	ns		Correlational	*n* = 291	Undergraduate psychology students aged 18-25y<br>*M* = 20.03 (*SD* = 3.06)	CIUT,<br>I-PACE, and,<br>DSMM
85	Dempsey et al., 2019	*FoMO:*<br>Fear of Missing Out Scale (FoMOs; Przybylski et al., 2013)<br><br>*Problematic Facebook use:*<br>Bergen Facebook Addiction Scale (BFAS; Andreassen et al., 2012)<br><br>*Social anxiety:*<br>The Patient Health Questionnaire-9 (PHQ-9; Spitzer et al., 1999)	Path 3	Social anxiety	Problematic Facebook use	FoMO			.12, *SE* = .03, *p* < .001	Correlational	*n* = 291	Undergraduate psychology students aged 18-25y<br>*M* = 20.03 (*SD* = 3.06)	CIUT,<br>I-PACE, and,<br>DSMM
85	Dempsey et al., 2019	*FoMO:*<br>Fear of Missing Out Scale (FoMOs; Przybylski et al., 2013)<br><br>*Facebook use frequency:*<br>Self-designed questionnaire asking how often participants engage in various Facebook features (e.g., "Change or update your status on Facebook," "Click the 'like' button next to other people's status, photos, links, or other posts on Facebook")	Path 2	FoMO	Facebook use frequency		-.19	**		Correlational	*n* = 291	Undergraduate psychology students aged 18-25y<br>*M* = 20.03 (*SD* = 3.06)	CIUT,<br>I-PACE, and,<br>DSMM

#Ref	Study	Measures	Hypothesized model	IV	DV	M	r	*p*	Indirect effects (β/ b [CI], SE, *p*)	Study design	Sample size	Participant characteristics	Theory
85	Dempsey et al., 2019	*FoMO:*<br>Fear of Missing Out Scale (FoMOs; Przybylski et al., 2013)<br><br>*Depression:*<br>The Patient Health Questionnaire-9 (PHQ-9; Spitzer et al., 1999)	Path 1	Depression	FoMO		.26	**		Correlational	*n* = 291	Undergraduate psychology students aged 18-25y<br>*M* = 20.03 (*SD* = 3.06)	CIUT,<br>I-PACE, and,<br>DSMM
85	Dempsey et al., 2019	*FoMO:*<br>Fear of Missing Out Scale (FoMOs; Przybylski et al., 2013)<br><br>*Problematic Facebook use:*<br>Bergen Facebook Addiction Scale (BFAS; Andreassen et al., 2012)	Path 2	FoMO	Problematic Facebook use		.32	**		Correlational	*n* = 291	Undergraduate psychology students aged 18-25y<br>*M* = 20.03 (*SD* = 3.06)	CIUT,<br>I-PACE, and,<br>DSMM
85	Dempsey et al., 2019	*FoMO:*<br>Fear of Missing Out Scale (FoMOs; Przybylski et al., 2013)<br><br>*Social anxiety:*<br>The Patient Health Questionnaire-9 (PHQ-9; Spitzer et al., 1999)	Path 1	Social anxiety	FoMO		.42	**		Correlational	*n* = 291	Undergraduate psychology students aged 18-25y<br>*M* = 20.03 (*SD* = 3.06)	CIUT,<br>I-PACE, and,<br>DSMM
85	Dempsey et al., 2019	*FoMO:*<br>Fear of Missing Out Scale (FoMOs; Przybylski et al., 2013)<br><br>*Problematic social media use (PSMU):*<br>Bergen Facebook Addiction Scale (BFAS; Andreassen et al., 2012)<br><br>*Depression:*<br>The Patient Health Questionnaire-9 (PHQ-9; Spitzer et al., 1999)	Path 3	Depression	PSMU	FoMO		ns		Correlational	*n* = 291	Undergraduate psychology students aged 18-25y<br>*M* = 20.03 (*SD* = 3.06)	CIUT,<br>I-PACE, and,<br>DSMM
85	Dempsey et al., 2019	*FoMO:*<br>Fear of Missing Out Scale (FoMOs; Przybylski et al., 2013)<br><br>*Problematic social media use (PSMU):*<br>Bergen Facebook Addiction Scale (BFAS; Andreassen et al., 2012)<br><br>*Life satisfaction (LS):*<br>The Satisfaction with Life Scale (SWLS; Diener et al., 1985)	Path 3	LS	PSMU	FoMO		ns		Correlational	*n* = 291	Undergraduate psychology students aged 18-25y<br>*M* = 20.03 (*SD* = 3.06)	CIUT,<br>I-PACE, and,<br>DSMM
54	Deniz, 2021	*FoMO:*<br>Fear of Missing Out Scale (FoMOs; Przybylski et al., 2013)<br><br>*Satisfaction with life (SwL):*<br>Satisfaction with Life Scale (SWLS; Diener et al., 1985)	Path 5	FoMO	SwL		-.41	***		Correlational	*n* = 323	Individuals aged 18-32years<br>*M* = 21.52 (*SD* = 2.69)	n/a
36	Dhir et al., 2018	*FoMO:*<br>3 items (Q1, Q2, Q3) of Fear of Missing Out Scale (FoMOs; Przybylski et al., 2013)<br><br>*Social media fatigue:*<br>Fatigue due to SNS use (FSNS; Bright et al., 2015)	Path 2	FoMO	Fatigue due to SNS use		Wave 1 .23	***		Correlational<br>Two waves (same schools; participants of wave 1 and 2 may or may not differ from each other; 5	Wave 1 *n* = 1554	Wave 1<br>Adolescent social media users aged 12-18y<br>M = 14.63 (SD = 1.73)	SSO
36	Dhir et al., 2018	*FoMO:*<br>3 items (Q1, Q2, Q3) of Fear of Missing Out Scale (FoMOs; Przybylski et al., 2013)<br><br>*Social media fatigue:*<br>Fatigue due to SNS use (FSNS; Bright et al., 2015)	Path 2	FoMO	Fatigue due to SNS use		Wave 2 .12	***		Correlational<br>Two waves (same schools; participants of wave 1 and 2 may or may not differ from each other; 5	Wave 2 *n* = 1144	Adolescent social media users aged 12–18y<br>*M* = 14.88 (*SD* = 1.41)	SSO
36	Dhir et al., 2018	*FoMO:*<br>3 items (Q1, Q2, Q3) of Fear of Missing Out Scale (FoMOs; Przybylski et al., 2013)<br><br>*Compulsive social networking site (SNS) use:*<br>Bergen Social Media Addiction Scale (BSMAS; Andreassen et al., 2012)	*No path specified*	FoMO	Compulsive SNS use		Wave 1 .37	***		Correlational<br>Two waves (same schools; participants of wave 1 and 2 may or may not differ from each other; 5 months apart)	Wave 1 *n* = 1554	Wave 1<br>Adolescent social media users aged 12-18y<br>M = 14.63 (SD = 1.73)	SSO
36	Dhir et al., 2018	*FoMO:*<br>3 items (Q1, Q2, Q3) of Fear of Missing Out Scale (FoMOs; Przybylski et al., 2013)<br><br>*Compulsive social networking site (SNS) use:*<br>Bergen Social Media Addiction Scale (BSMAS; Andreassen et al., 2012)	*No path specified*	FoMO	Compulsive SNS use		Wave 2 .24	***		Correlational<br>Two waves (same schools; participants of wave 1 and 2 may or may not differ from each other; 5 months apart)	Wave 2 *n* = 1144	Adolescent social media users aged 12–18y<br>*M* = 14.88 (*SD* = 1.41)	SSO

#Ref	Study	Measures	Hypothesized model	IV	DV	M	r	*p*	Indirect effects (β/ b [CI], SE, *p*)	Study design	Sample size	Participant characteristics	Theory
36	Dhir et al., 2018	FoMO:<br>3 items (Q1, Q2, Q3) of Fear of Missing Out Scale (FoMOs; Przybylski et al., 2013)<br><br>*Depression:*<br>Depression (DEP; Salokangas et al., 1995)	Path 5	FoMO	Depression		Wave 1 .53	***		Correlational<br>Two waves (same schools; participants of wave 1 and 2 may or may not differ from each other; 5	Wave 1 *n* = 1554	Wave 1<br>Adolescent social media users aged 12-18y<br>M = 14.63 (SD = 1.73)	SSO
36	Dhir et al., 2018	FoMO:<br>3 items (Q1, Q2, Q3) of Fear of Missing Out Scale (FoMOs; Przybylski et al., 2013)<br><br>*Depression:*<br>Depression (DEP; Salokangas et al., 1995)	Path 5	FoMO	Depression		Wave 2 .64	***		Correlational<br>Two waves (same schools; participants of wave 1 and 2 may or may not differ from each other; 5	Wave 2 *n* = 1144	Adolescent social media users aged 12–18y<br>*M* = 14.88 (*SD* = 1.41)	SSO
36	Dhir et al., 2018	*FoMO:*<br>3 items (Q1, Q2, Q3) of Fear of Missing Out Scale (FoMOs; Przybylski et al., 2013)<br><br>*Anxiety:*<br>Anxiety (AX; La Greca & Lopez, 1998)	Path 5	FoMO	Anxiety		Wave 1 .61	***		Correlational<br>Two waves (same schools; participants of wave 1 and 2 may or may not differ from each other; 5	Wave 1 *n* = 1554	Wave 1<br>Adolescent social media users aged 12-18y<br>M = 14.63 (SD = 1.73)	SSO
36	Dhir et al., 2018	*FoMO:*<br>3 items (Q1, Q2, Q3) of Fear of Missing Out Scale (FoMOs; Przybylski et al., 2013)<br><br>*Anxiety:*<br>Anxiety (AX; La Greca & Lopez, 1998)	Path 5	FoMO	Anxiety		Wave 2 .67	***		Correlational<br>Two waves (same schools; participants of wave 1 and 2 may or may not differ from each other; 5	Wave 2 *n* = 1144	Adolescent social media users aged 12–18y<br>*M* = 14.88 (*SD* = 1.41)	SSO
109	D'Lima & Higgins, 2021	*FoMO:*<br>Fear of Missing Out Scale (FoMOs; Przybylski et al., 2013)<br><br>*Social media engagement (SME):*<br>Social Media Engagement questionnaire (SMEQ; Przybylski et al., 2013)	*No path specified*	FoMO	SME		.22	*		Correlational	*n* = 91	Primary school children aged 9-11y<br>*M* = 10.04 (*SD* = 0.76)	SDT
33	Duan & Tang, 2020	*FoMO:*<br>Fear of Missing Out Scale (FoMOs; Przybylski et al., 2013)<br><br>*Online social anxiety (OSA):*<br>Social Anxiety Scale for Social Media Users (SAS-SMU; Alkis et al., 2017)	Path 5	FoMO	OSA		.49	**		Correlational	*n* = 796	Social media users<br>*M* = 21.12 (*SD* = 1.67)	SDT, The Conceptualization Model of Social Anxiety and Self-presentation
134	Durak & Seferoğlu, 2020	*FoMO:*<br>Fear of Missing Out Scale (FoMOs; Przybylski et al., 2013)<br><br>*Social media use (SMU) frequency:*<br>Self-designed questionnaire on social media usage frequency	Path 2	FoMO	SMU frequency		.16	***		Correlational	*n* = 1284	University students that use social media<br>*M* = 23.04	n/a
134	Durak & Seferoğlu, 2020	*FoMO:*<br>Fear of Missing Out Scale (FoMOs; Przybylski et al., 2013)<br><br>*Number of friends in social media environments:*<br>Self-designed questionnaire on average number of friends/ followers possessed in social media environments	Path 2	FoMO	Number of friends in social media environments		.21	***		Correlational	*n* = 1284	University students that use social media<br>*M* = 23.04	n/a
92	Eide et al., 2018	*FoMO:*<br>Fear of Missing Out Scale (FoMOs; Przybylski et al., 2013)	Path 4	Smartphone use	FoMO		.18	*		Causal/ experiment<br>Randomly assigned to: 1) hand in smartphone for 72h or 2) use smartphone as usual	*n* = 127<br>Experimental group *n* = 67<br>Control group *n* = 60	Smartphone users who use phone for at least 1h on a daily basis aged 18-48y<br>*M* = 25.0 (*SD* = 4.8)	n/a
92	Eide et al., 2018	*FoMO:*<br>Fear of Missing Out Scale (FoMOs; Przybylski et al., 2013)	Path 4	Smartphone use	FoMO		.26	**		Causal/ experiment<br>Randomly assigned to: 1) hand in smartphone for 72h or 2) use smartphone as usual	*n* = 127<br>Experimental group *n* = 67<br>Control group *n* = 60	Smartphone users who use phone for at least 1h on a daily basis aged 18-48y<br>*M* = 25.0 (*SD* = 4.8)	n/a

#Ref	Study	Measures	Hypothesized model	IV	DV	M	r	*p*	Indirect effects (β/ b [CI], SE, *p*)	Study design	Sample size	Participant characteristics	Theory
45	Elhai et al., 2018	*FoMO:*<br>Fear of Missing Out Scale (FoMOs; Przybylski et al., 2013)<br><br>*Smartphone use frequency (SUF):*<br>Smartphone use frequency scale (Elhai et al., 2016)<br><br>*Rumination:*<br>Ruminative thought style questionnaire (RTSQ; Brinker & Dozois, 2009)	Path 3	Rumination	SUF	FoMO	.07	ns		Correlational	*n* = 296	Undergraduate university students aged 18-25y<br>*M* = 19.44 (*SD* = 2.16)	SDT
45	Elhai et al., 2018	*FoMO:*<br>Fear of Missing Out Scale (FoMOs; Przybylski et al., 2013)<br><br>*Smartphone use frequency (SUF):*<br>Smartphone use frequency scale (Elhai et al., 2016)<br><br>*Stress:*<br>Depression anxiety stress Scale-21 (DASS-21; Lovibond & Lovibond, 1995)	Path 3	Stress	SUF	FoMO	.10	ns		Correlational	*n* = 296	Undergraduate university students aged 18-25y<br>*M* = 19.44 (*SD* = 2.16)	SDT
45	Elhai et al., 2018	*FoMO:*<br>Fear of Missing Out Scale (FoMOs; Przybylski et al., 2013)<br><br>*Smartphone use frequency (SUF):*<br>Smartphone use frequency scale (Elhai et al., 2016)<br><br>*Anxiety:*<br>Depression anxiety stress Scale-21 (DASS-21; Lovibond & Lovibond, 1995)	Path 3	Anxiety	SUF	FoMO	.11	*		Correlational	*n* = 296	Undergraduate university students aged 18-25y<br>*M* = 19.44 (*SD* = 2.16)	SDT
45	Elhai et al., 2018	*FoMO:*<br>Fear of Missing Out Scale (FoMOs; Przybylski et al., 2013)<br><br>*Smartphone use frequency (SUF):*<br>Smartphone use frequency scale (Elhai et al., 2016)<br><br>*Boredom proneness:*<br>Boredom proneness scale-short form (BPS-S; Struk et al., 2017)	Path 3	Boredom proneness	SUF	FoMO	.11	*		Correlational	*n* = 296	Undergraduate university students aged 18-25y<br>*M* = 19.44 (*SD* = 2.16)	SDT
45	Elhai et al., 2018	*FoMO:*<br>Fear of Missing Out Scale (FoMOs; Przybylski et al., 2013)<br><br>*Smartphone use frequency (SUF):*<br>Smartphone use frequency scale (Elhai et al., 2016)<br><br>*Depression:*<br>Depression anxiety stress Scale-21 (DASS-21; Lovibond & Lovibond, 1995)	Path 3	Depression	SUF	FoMO	.12	*		Correlational	*n* = 296	Undergraduate university students aged 18-25y<br>*M* = 19.44 (*SD* = 2.16)	SDT
45	Elhai et al., 2018	*FoMO:*<br>Fear of Missing Out Scale (FoMOs; Przybylski et al., 2013)<br><br>*Smartphone use frequency (SUF):*<br>Smartphone use frequency scale (Elhai et al., 2016)	Path 2	FoMO	SUF		.13	*		Correlational	*n* = 296	Undergraduate university students aged 18-25y<br>*M* = 19.44 (*SD* = 2.16)	SDT
45	Elhai et al., 2018	*FoMO:*<br>Fear of Missing Out Scale (FoMOs; Przybylski et al., 2013)<br><br>*Social use:*<br>Process and Social Use Scale (van Deursen et al., 2015)	Path 2	FoMO	Social use		.20	***		Correlational	*n* = 296	Undergraduate university students aged 18-25y<br>*M* = 19.44 (*SD* = 2.16)	SDT
45	Elhai et al., 2018	*FoMO:*<br>Fear of Missing Out Scale (FoMOs; Przybylski et al., 2013)<br><br>*Problematic smartphone use (PSU):*<br>Smartphone Addiction Scale (SAS; Kwon et al., 2013)<br><br>*Boredom proneness:*<br>Boredom proneness scale-short form (BPS-S; Struk et al., 2017)	Path 3	Boredom proneness	PSU	FoMO	.22	***		Correlational	*n* = 296	Undergraduate university students aged 18-25y<br>*M* = 19.44 (*SD* = 2.16)	SDT

#Ref	Study	Measures	Hypothesized model	IV	DV	M	r	*p*	Indirect effects (β/ b [CI], SE, *p*)	Study design	Sample size	Participant characteristics	Theory
45	Elhai et al., 2018	*FoMO:*<br>Fear of Missing Out Scale (FoMOs; Przybylski et al., 2013)<br><br>*Problematic smartphone use (PSU):*<br>Smartphone Addiction Scale (SAS; Kwon et al., 2013)<br><br>*Rumination:*<br>Ruminative thought style questionnaire (RTSQ; Brinker & Dozois, 2009)	Path 3	Rumination	PSU	FoMO	.24	***		Correlational	*n* = 296	Undergraduate university students aged 18-25y<br>*M* = 19.44 (*SD* = 2.16)	SDT
45	Elhai et al., 2018	*FoMO:*<br>Fear of Missing Out Scale (FoMOs; Przybylski et al., 2013)<br><br>*Problematic smartphone use (PSU):*<br>Smartphone Addiction Scale (SAS; Kwon et al., 2013)<br><br>*Anxiety:*<br>Depression Anxiety Stress Scale-21 (DASS-21; Lovibond & Lovibond, 1995)	Path 3	Anxiety	PSU	FoMO	.25	***		Correlational	*n* = 296	Undergraduate university students aged 18-25y<br>*M* = 19.44 (*SD* = 2.16)	SDT
45	Elhai et al., 2018	*FoMO* :<br>Fear of Missing Out Scale (FoMOs; Przybylski et al., 2013)<br><br>*Problematic smartphone use (PSU):*<br>Smartphone Addiction Scale (SAS; Kwon et al., 2013)<br><br>*Stress:*<br>Depression Anxiety Stress Scale-21 (DASS-21; Lovibond & Lovibond, 1995)	Path 3	Stress	PSU	FoMO	.25	***		Correlational	*n* = 296	Undergraduate university students aged 18-25y<br>*M* = 19.44 (*SD* = 2.16)	SDT
45	Elhai et al., 2018	*FoMO:*<br>Fear of Missing Out Scale (FoMOs; Przybylski et al., 2013)<br><br>*Problematic smartphone use (PSU):*<br>Smartphone Addiction Scale (SAS; Kwon et al., 2013)<br><br>*Depression:*<br>Depression Anxiety Stress Scale-21 (DASS-21; Lovibond & Lovibond, 1995)	Path 3	Depression	PSU	FoMO	.26	***		Correlational	*n* = 296	Undergraduate university students aged 18-25y<br>*M* = 19.44 (*SD* = 2.16)	SDT
45	Elhai et al., 2018	*FoMO:*<br>Fear of Missing Out Scale (FoMOs; Przybylski et al., 2013)<br><br>*Boredom proneness:*<br>Boredom proneness scale-short form (BPS-S; Struk et al., 2017)	Path 5	FoMO	Boredom proneness		.42	***		Correlational	*n* = 296	Undergraduate university students aged 18-25y<br>*M* = 19.44 (*SD* = 2.16)	SDT
45	Elhai et al., 2018	*FoMO:*<br>Fear of Missing Out Scale (FoMOs; Przybylski et al., 2013)<br><br>*Rumination:*<br>Ruminative thought style questionnaire (RTSQ; Brinker & Dozois, 2009)	Path 5	FoMO	Rumination		.43	***		Correlational	*n* = 296	Undergraduate university students aged 18-25y<br>*M* = 19.44 (*SD* = 2.16)	SDT
45	Elhai et al., 2018	*FoMO:*<br>Fear of Missing Out Scale (FoMOs; Przybylski et al., 2013)<br><br>*Process use:*<br>Process and Social Use Scale (van Deursen et al., 2015)	Path 2	FoMO	Process use		.45	***		Correlational	*n* = 296	Undergraduate university students aged 18-25y<br>*M* = 19.44 (*SD* = 2.16)	SDT
45	Elhai et al., 2018	*FoMO:*<br>Fear of Missing Out Scale (FoMOs; Przybylski et al., 2013)<br><br>*Anxiety:*<br>Depression Anxiety Stress Scale-21 (DASS-21; Lovibond & Lovibond, 1995)	Path 5	FoMO	Anxiety		.46	***		Correlational	*n* = 296	Undergraduate university students aged 18-25y<br>*M* = 19.44 (*SD* = 2.16)	SDT
45	Elhai et al., 2018	*FoMO:*<br>Fear of Missing Out Scale (FoMOs; Przybylski et al., 2013)<br><br>*Depression:*<br>Depression Anxiety Stress Scale-21 (DASS-21; Lovibond & Lovibond, 1995)	Path 5	FoMO	Depression		.47	***		Correlational	*n* = 296	Undergraduate university students aged 18-25y<br>*M* = 19.44 (*SD* = 2.16)	SDT

#Ref	Study	Measures	Hypothesized model	IV	DV	M	r	*p*	Indirect effects (β/ b [CI], SE, *p*)	Study design	Sample size	Participant characteristics	Theory
45	Elhai et al., 2018	*FoMO:*<br>Fear of Missing Out Scale (FoMOs; Przybylski et al., 2013)<br><br>*Problematic smartphone use (PSU):*<br>Smartphone Addiction Scale (SAS; Kwon et al., 2013)	Path 2	FoMO	PSU		.51	***		Correlational	*n* = 296	Undergraduate university students aged 18-25y<br>*M* = 19.44 (*SD* = 2.16)	SDT
45	Elhai et al., 2018	*FoMO:*<br>Fear of Missing Out Scale (FoMOs; Przybylski et al., 2013)<br><br>*Stress:*<br>Depression Anxiety Stress Scale-21 (DASS-21; Lovibond & Lovibond, 1995)	Path 5	FoMO	Stress		.51	***		Correlational	*n* = 296	Undergraduate university students aged 18-25y<br>*M* = 19.44 (*SD* = 2.16)	SDT
27	Elhai et al., 2021	*FoMO:*<br>Fear of Missing Out Scale (FoMOs; Przybylski et al., 2013)<br><br>*Depression:*<br>Depression Anxiety Stress Scale-21 (DASS-21; Lovibond & Lovibond, 1995)	*No path specified*	Depression	FoMO		.23	***		Correlational	*n* = 296	Undergraduate university students aged 18-25y<br>*M* = 19.44 (*SD* = 2.16)	SDT, and, I-PACE
27	Elhai et al., 2021	*FoMO:*<br>Fear of Missing Out Scale (FoMOs; Przybylski et al., 2013)<br><br>*Anxiety:*<br>Depression Anxiety Stress Scale-21 (DASS-21; Lovibond & Lovibond, 1995)	*No path specified*	Anxiety	FoMO		.27	***		Correlational	*n* = 296	Undergraduate university students aged 18-25y<br>*M* = 19.44 (*SD* = 2.16)	SDT, and, I-PACE
27	Elhai et al., 2021	*FoMO:*<br>Fear of Missing Out Scale (FoMOs; Przybylski et al., 2013)<br><br>*Anxiety:*<br>Depression Anxiety Stress Scale-21 (DASS-21; Lovibond & Lovibond, 1995)	*No path specified*	Stress	FoMO		.27	***		Correlational	*n* = 296	Undergraduate university students aged 18-25y<br>*M* = 19.44 (*SD* = 2.16)	SDT, and, I-PACE
57	Elhai, Gallinari, Rozgonjuk, et al., 2020	*FoMO:*<br>Fear of Missing Out Scale (FoMOs; Przybylski et al., 2013)<br><br>*Social use:*<br>Process and Social Use Scale (van Deursen et al., 2015)<br><br>*Depression:*<br>Depression Anxiety Stress Scale-21 (DASS-21; Lovibond & Lovibond, 1995)	Path 3	Depression	Social use	FoMO	.01	ns		Correlational	*n* = 316	Undergraduate students aged 18-25y<br>*M* = 19.21 (*SD* = 1.74)	SDT, and, I-PACE
57	Elhai, Gallinari, Rozgonjuk, et al., 2020	*FoMO:*<br>Fear of Missing Out Scale (FoMOs; Przybylski et al., 2013)<br><br>*Process use:*<br>Process and Social use scale (van Deursen et al., 2015)<br><br>*Depression:*<br>Depression Anxiety Stress Scale-21 (DASS-21; Lovibond & Lovibond, 1995)	Path 3	Depression	Process use	FoMO	.14	*		Correlational	*n* = 316	Undergraduate students aged 18-25y<br>*M* = 19.21 (*SD* = 1.74)	SDT, and, I-PACE
57	Elhai, Gallinari, Rozgonjuk, et al., 2020	*FoMO:*<br>Fear of Missing Out Scale (FoMOs; Przybylski et al., 2013)<br><br>*Process use:*<br>Process and Social Use Scale (van Deursen et al., 2015)<br><br>*Anxiety:*<br>Depression Anxiety Stress Scale-21 (DASS-21; Lovibond & Lovibond, 1995)	Path 3	Anxiety	Process use	FoMO	.11	ns		Correlational	*n* = 316	Undergraduate students aged 18-25y<br>*M* = 19.21 (*SD* = 1.74)	SDT, and, I-PACE

#Ref	Study	Measures	Hypothesized model	IV	DV	M	r	*p*	Indirect effects (β/ b [CI], SE, *p*)	Study design	Sample size	Participant characteristics	Theory
57	Elhai, Gallinari, Rozgonjuk, et al., 2020	*FoMO:* Fear of Missing Out Scale (FoMOs; Przybylski et al., 2013)<br><br>*Social use:* Process and Social Use Scale (van Deursen et al., 2015)<br><br>*Anxiety:* Depression Anxiety Stress Scale-21 (DASS-21; Lovibond & Lovibond, 1995)	Path 3	Anxiety	Social use	FoMO	.08	ns		Correlational	*n* = 316	Undergraduate students aged 18-25y *M* = 19.21 (*SD* = 1.74)	SDT, and, I-PACE
57	Elhai, Gallinari, Rozgonjuk, et al., 2020	*FoMO:* Fear of Missing Out Scale (FoMOs; Przybylski et al., 2013)<br><br>*Social use:* Process and Social Use Scale (van Deursen et al., 2015)	Path 2	FoMO	Social use		.13	*		Correlational	*n* = 316	Undergraduate students aged 18-25y *M* = 19.21 (*SD* = 1.74)	SDT, and, I-PACE
57	Elhai, Gallinari, Rozgonjuk, et al., 2020	*FoMO:* Fear of Missing Out Scale (FoMOs; Przybylski et al., 2013)<br><br>*Problematic smartphone use (PSU):* Smartphone addiction scale-short version (SAS-SV; Kwon et al., 2013)<br><br>*Anxiety:* Depression Anxiety Stress Scale-21 (DASS-21; Lovibond & Lovibond,	Path 3	Anxiety	PSU	FoMO			.16, *SE* = .07, *p* = .04	Correlational	*n* = 316	Undergraduate students aged 18-25y *M* = 19.21 (*SD* = 1.74)	SDT, and, I-PACE
57	Elhai, Gallinari, Rozgonjuk, et al., 2020	*FoMO:* Fear of Missing Out Scale (FoMOs; Przybylski et al., 2013)<br><br>*Process use:* Process and Social Use Scale (van Deursen et al., 2015)	Path 2	FoMO	Process use		.27	**		Correlational	*n* = 316	Undergraduate students aged 18-25y *M* = 19.21 (*SD* = 1.74)	SDT, and, I-PACE
57	Elhai, Gallinari, Rozgonjuk, et al., 2020	*FoMO:* Fear of Missing Out Scale (FoMOs; Przybylski et al., 2013)<br><br>*Problematic smartphone use (PSU):* Smartphone addiction scale-short version (SAS-SV; Kwon et al., 2013)<br><br>*Depression:* Depression Anxiety Stress Scale-21 (DASS-21; Lovibond & Lovibond,	Path 3	Depression	PSU	FoMO			.23, *SE* = .06, *p* < .001	Correlational	*n* = 316	Undergraduate students aged 18-25y *M* = 19.21 (*SD* = 1.74)	SDT, and, I-PACE
57	Elhai, Gallinari, Rozgonjuk, et al., 2020	*FoMO:* Fear of Missing Out Scale (FoMOs; Przybylski et al., 2013)<br><br>*Anxiety:* Depression Anxiety Stress Scale-21 (DASS-21; Lovibond & Lovibond, 1995)	Path 1	Anxiety	FoMO		.40	**		Correlational	*n* = 316	Undergraduate students aged 18-25y *M* = 19.21 (*SD* = 1.74)	SDT, and, I-PACE
57	Elhai, Gallinari, Rozgonjuk, et al., 2020	*FoMO:* Fear of Missing Out Scale (FoMOs; Przybylski et al., 2013)<br><br>*Depression:* Depression Anxiety Stress Scale-21 (DASS-21; Lovibond & Lovibond, 1995)	Path 1	Depression	FoMO		.44	**		Correlational	*n* = 316	Undergraduate students aged 18-25y *M* = 19.21 (*SD* = 1.74)	SDT, and, I-PACE
57	Elhai, Gallinari, Rozgonjuk, et al., 2020	*FoMO:* Fear of Missing Out Scale (FoMOs; Przybylski et al., 2013)<br><br>*Problematic smartphone use (PSU):* Smartphone addiction scale-short version (SAS-SV; Kwon et al.,	Path 2	FoMO	PSU		.51	**		Correlational	*n* = 316	Undergraduate students aged 18-25y *M* = 19.21 (*SD* = 1.74)	SDT, and, I-PACE
87	Elhai, Yang, Fang, et al., 2020	*FoMO:* Fear of Missing Out Scale (FoMOs; Przybylski et al., 2013)<br><br>*Smartphone use frequency (SUF):* Smartphone use frequency scale (Elhai et al., 2016)<br><br>*Depression:* Depression Anxiety Stress Scale-21 (DASS-21; Lovibond & Lovibond, 1995)	Path 3	Depression	SUF	FoMO			.03, *SE* = .05, *p* = .53	Correlational	*n* = 1034	Psychology faculty students aged 16+ *M* = 19.43 (*SD* = 1.61)	CIUT, and, I-PACE

#Ref	Study	Measures	Hypothesized model	IV	DV	M	r	*p*	Indirect effects (β/ b [CI], SE, *p*)	Study design	Sample size	Participant characteristics	Theory
87	Elhai, Yang, Fang, et al., 2020	*FoMO:* Fear of Missing Out Scale (FoMOs; Przybylski et al., 2013)<br><br>*Problematic smartphone use (PSU):* Smartphone Addiction Scale-Short version (SAS-SV; Kwon et al., 2013)<br><br>*Depression:* Depression Anxiety Stress Scale-21 (DASS-21; Lovibond & Lovibond,	Path 3	Depression	PSU	FoMO			-.12, *SE* = .19, *p* = .53	Correlational	*n* = 1034	Psychology faculty students aged 16+ *M* = 19.43 (*SD* = 1.61)	CIUT, and, I-PACE
87	Elhai, Yang, Fang, et al., 2020	*FoMO:* Fear of Missing Out Scale (FoMOs; Przybylski et al., 2013)<br><br>*Smartphone use frequency (SUF)* : Smartphone use frequency scale (Elhai et al., 2016)	Path 2	FoMO	SUF		.20	***		Correlational	*n* = 1034	Psychology faculty students aged 16+ *M* = 19.43 (*SD* = 1.61)	CIUT, and, I-PACE
87	Elhai, Yang, Fang, et al., 2020	*FoMO:* Fear of Missing Out Scale (FoMOs; Przybylski et al., 2013)<br><br>*Smartphone use frequency (SUF):* Smartphone use frequency scale (Elhai et al., 2016)<br><br>*Anxiety:* Depression Anxiety Stress Scale-21 (DASS-21; Lovibond & Lovibond, 1995)	Path 3	Anxiety	SUF	FoMO			.16, *SE* = .06, *p* = .006	Correlational	*n* = 1034	Psychology faculty students aged 16+ *M* = 19.43 (*SD* = 1.61)	CIUT, and, I-PACE
87	Elhai, Yang, Fang, et al., 2020	*FoMO:* Fear of Missing Out Scale (FoMOs; Przybylski et al., 2013)<br><br>*Depression:* Depression Anxiety Stress Scale-21 (DASS-21; Lovibond & Lovibond, 1995)	Path 1	Depression	FoMO		.29	***		Correlational	*n* = 1034	Psychology faculty students aged 16+ *M* = 19.43 (*SD* = 1.61)	CIUT, and, I-PACE
87	Elhai, Yang, Fang, et al., 2020	*FoMO:* Fear of Missing Out Scale (FoMOs; Przybylski et al., 2013)<br><br>*Problematic smartphone use (PSU):* Smartphone Addiction Scale-Short version (SAS-SV; Kwon et al.,	Path 2	FoMO	PSU		.29	***		Correlational	*n* = 1034	Psychology faculty students aged 16+ *M* = 19.43 (*SD* = 1.61)	CIUT, and, I-PACE
87	Elhai, Yang, Fang, et al., 2020	*FoMO:* Fear of Missing Out Scale (FoMOs; Przybylski et al., 2013)<br><br>*Anxiety:* Depression Anxiety Stress Scale-21 (DASS-21; Lovibond & Lovibond, 1995)	Path 1	Anxiety	FoMO		.33	***		Correlational	*n* = 1034	Psychology faculty students aged 16+ *M* = 19.43 (*SD* = 1.61)	CIUT, and, I-PACE
87	Elhai, Yang, Fang, et al., 2020	*FoMO:* Fear of Missing Out Scale (FoMOs; Przybylski et al., 2013)<br><br>*Problematic smartphone use (PSU):* Smartphone Addiction Scale-Short version (SAS-SV; Kwon et al., 2013)<br><br>*Anxiety:* Depression Anxiety Stress Scale-21 (DASS-21; Lovibond & Lovibond,	Path 3	Anxiety	PSU	FoMO			.63, *SE* = .19, *p* < .001	Correlational	*n* = 1034	Psychology faculty students aged 16+ *M* = 19.43 (*SD* = 1.61)	CIUT, and, I-PACE
104	Elhai, Yang, Rozgonjuk, et al., 2020	*FoMO:* Fear of Missing Out Scale (FoMOs; Przybylski et al., 2013)<br><br>*Problematic smartphone use (PSU):* Smartphone Addiction Scale-Short Version (SAS-SV; Kwon et al.,	Path 2	FoMO	PSU		.40	**		Correlational	*n* = 1097	Undergraduate students *M* = 19.38 (*SD* = 1.18)	CIUT, and, I-PACE
104	Elhai, Yang, Rozgonjuk, et al., 2020	*FoMO:* Fear of Missing Out Scale (FoMOs; Przybylski et al., 2013)<br><br>*Rumination:* Ruminative thought style questionnaire (RTSQ; Brinker & Dozois, 2009)	*Path 5*	FoMO	Rumination		.56	**		Correlational	*n* = 1097	Undergraduate students *M* = 19.38 (*SD* = 1.18)	CIUT, and, I-PACE

#Ref	Study	Measures	Hypothesized model	IV	DV	M	r	*p*	Indirect effects (β/ b [CI], SE, *p*)	Study design	Sample size	Participant characteristics	Theory
104	Elhai, Yang, Rozgonjuk, et al., 2020	*FoMO:*<br>Fear of Missing Out Scale (FoMOs; Przybylski et al., 2013)<br><br>*Depression:*<br>Depression Anxiety Stress Scale-21 (DASS-21; Lovibond & Lovibond, 1995)	*Path 5*	*FoMO*	Depression		*.57*	**		Correlational	*n* = 1097	Undergraduate students<br>*M* = 19.38 (*SD* = 1.18)	I-PACE
104	Elhai, Yang, Rozgonjuk, et al., 2020	*FoMO:*<br>Fear of Missing Out Scale (FoMOs; Przybylski et al., 2013)<br><br>*Anxiety:*<br>Depression Anxiety Stress Scale-21 (DASS-21; Lovibond & Lovibond, 1995)	*Path 5*	FoMO	Anxiety		.63	**		Correlational	*n* = 1097	Undergraduate students<br>*M* = 19.38 (*SD* = 1.18)	I-PACE
90	Fabris et al., 2020	*FoMO:*<br>Fear of Missing Out Scale (FoMOs; Przybylski et al., 2013)<br><br>*Sensitivity to stress associated negative reactions by online peers (SS-NeR):*<br>Self-designed questionnaire assessing sensitivity to stress associated with experiences of negative reactions by other users	Path 5	FoMO	SS-NeR		.26	**		Correlational	*n* = 472	Middle school students aged 11-19y<br>*M* = 13.49 (*SD* = 1.87)	SDT, and, I-PACE
90	Fabris et al., 2020	*FoMO:*<br>Fear of Missing Out Scale (FoMOs; Przybylski et al., 2013)<br><br>*Sensitivity to stress associated with neglect by online peers (SS-N):*<br>Self-designed questionnaire assessing sensitivity to stress associated with experiences of neglect by other users	Path 5	FoMO	SS-N		.31	**		Correlational	*n* = 472	Middle school students aged 11-19y<br>*M* = 13.49 (*SD* = 1.87)	SDT, and, I-PACE
90	Fabris et al., 2020	*FoMO:*<br>Fear of Missing Out Scale (FoMOs; Przybylski et al., 2013)<br><br>*Emotional symptoms:*<br>Emotional symptoms subscale of Strengths and Difficulties Questionnaire (SDQ; Goodman et al., 1998)	Path 5	FoMO	Emotional symptoms		.40	**		Correlational	*n* = 472	Middle school students aged 11-19y<br>*M* = 13.49 (*SD* = 1.87)	SDT, and, I-PACE
90	Fabris et al., 2020	*FoMO:*<br>Fear of Missing Out Scale (FoMOs; Przybylski et al., 2013)<br><br>*Social media addiction (SMA):*<br>Bergen Social Media Addiction Scale (BSMAS; Andreassen et al., 2016)	Path 2	FoMO	SMA		.48	**		Correlational	*n* = 472	Middle school students aged 11-19y<br>*M* = 13.49 (*SD* = 1.87)	SDT, and, I-PACE
88	Fitz et al, 2019	*Phone FoMO:*<br>C-FoMO-Scale (Hato, 2013)<br><br>*Anxiety:*<br>Self-designed question "Today, I felt anxious", and six item version of State-Trait Anxiety (STAI; Marteau & Bekker, 1992)	Path 6	Never receiving notifications	Anxiety	FoMO			b = 0.27 [0.09, 0.53]	Causal/ experiment	*n* = 237	MTurk smartphone owners<br>*M* = 30.3	n/a
88	Fitz et al, 2019	*Phone FoMO:*<br>C-FoMO-Scale (Hato, 2013)<br><br>Anxiety:<br>Self-designed question "Today, I felt anxious", and six item version of State-Trait Anxiety (STAI; Marteau & Bekker, 1992)	Path 5	Phone FoMO	Anxiety		.31	***		Causal/ experiment	*n* = 237	MTurk smartphone owners<br>*M* = 30.3	n/a
88	Fitz et al, 2019	*Phone FoMO:*<br>C-FoMO-Scale (Hato, 2013)	Path 4	Notifications batched 3x per day	Phone FoMO		.21	**		Causal/ experiment	*n* = 237	MTurk smartphone owners<br>*M* = 30.3	n/a
88	Fitz et al, 2019	*Phone FoMO:*<br>C-FoMO-Scale (Hato, 2013)	Path 4	Notifications batched hourly	Phone FoMO		.16	*		Causal/ experiment	*n* = 237	MTurk smartphone owners<br>*M* = 30.3	n/a
88	Fitz et al, 2019	*Phone FoMO:*<br>C-FoMO-Scale (Hato, 2013)	Path 4	No notifications	Phone FoMO		.29	***		Causal/ experiment	*n* = 237	MTurk smartphone owners<br>*M* = 30.3	n/a
88	Fitz et al, 2019	*Notifications FoMO:*<br>Self-designed 1-item measure "Today, I felt I was missing out on important notifications"	Path 4	Notifications batched 3x per day	Notification FoMO		.32	***		Causal/ experiment	*n* = 237	MTurk smartphone owners<br>*M* = 30.3	n/a
88	Fitz et al, 2019	*Notifications FoMO:*<br>Self-designed 1-item measure "Today, I felt I was missing out on important notifications"	Path 4	Notifications batched hourly	Notification FoMO		.20	**		Causal/ experiment	*n* = 237	MTurk smartphone owners<br>*M* = 30.3	n/a
88	Fitz et al, 2019	*Notifications FoMO:*<br>Self-designed 1-item measure "Today, I felt I was missing out on important notifications"	Path 4	No notifications	Notification FoMO		.26	***		Causal/ experiment	*n* = 237	MTurk smartphone owners<br>*M* = 30.3	n/a

#Ref	Study	Measures	Hypothesized model	IV	DV	M	r	*p*	Indirect effects (β/ b [CI], SE, *p)*	Study design	Sample size	Participant characteristics	Theory
39	Franchina et al., 2018	*FoMO:*<br>4 items (Q2, Q5, Q7, Q10) of Fear of Missing Out Scale (FoMOs; Przybylski et al., 2013)<br><br>*Frequency of YouTube use:*<br>Self-designed questionnaire asking for each active platform how frequently it was used	Path 2	FoMO	Frequency of YouTube use		.00	ns		Correlational	*n* = 2663	High school pupils<br>*M* = 14.87 (*SD* = 1.67)	n/a
39	Franchina et al., 2018	*FoMO:*<br>4 items (Q2, Q5, Q7, Q10) of Fear of Missing Out Scale (FoMOs; Przybylski et al., 2013)<br><br>*Frequency of Twitter use:*<br>Self-designed questionnaire asking for each active platform how frequently it was used	Path 2	FoMO	Frequency of Twitter use		.06	***		Correlational	*n* = 2663	High school pupils<br>*M* = 14.87 (*SD* = 1.67)	n/a
39	Franchina et al., 2018	*FoMO:*<br>4 items (Q2, Q5, Q7, Q10) of Fear of Missing Out Scale (FoMOs; Przybylski et al., 2013)<br><br>*Frequency of Facebook use:*<br>Self-designed questionnaire asking for each active platform how frequently it was used	Path 2	FoMO	Frequency of Facebook use		.16	***		Correlational	*n* = 2663	High school pupils<br>*M* = 14.87 (*SD* = 1.67)	n/a
39	Franchina et al., 2018	*FoMO:*<br>4 items (Q2, Q5, Q7, Q10) of Fear of Missing Out Scale (FoMOs; Przybylski et al., 2013)<br><br>*Frequency of Snapchat use:*<br>Self-designed questionnaire asking for each active platform how frequently it was used	Path 2	FoMO	Frequency of Snapchat use		.17	***		Correlational	*n* = 2663	High school pupils<br>*M* = 14.87 (*SD* = 1.67)	n/a
111	Fumagalli et al., 2021	*FoMO:*<br>3 items (Q3, Q6, Q8) of Fear of Missing Out Scale (FoMOs; Przybylski et al., 2013)<br><br>*Loneliness:*<br>UCLA Loneliness Scale (Hays & DiMatteo, 1987)	Path 5	FoMO	Loneliness		.26	***		Correlational	*n* = 334	Young adults<br>*M* = 21.50 (*SD* = 2.03)	n/a
111	Fumagalli et al., 2021	*FoMO:*<br>3 items (Q3, Q6, Q8) of Fear of Missing Out Scale (FoMOs; Przybylski et al., 2013)<br><br>*Social networking apps usage:*<br>Coded smartphone screenshots, summing usage of social network apps<br><br>*Loneliness:*<br>UCLA Loneliness Scale (Hays & DiMatteo, 1987)	Path 6	Social networking apps usage (objectively measured)	FoMO	Loneliness			b = .002 [0.001; 0.005], *p* = .001	Correlational	*n* = 334	Young adults<br>*M* = 21.50 (*SD* = 2.03)	n/a
111	Fumagalli et al., 2021	*FoMO:*<br>3 items (Q3, Q6, Q8) of Fear of Missing Out Scale (FoMOs; Przybylski et al., 2013)<br><br>*Messaging and VoIP apps usage:*<br>Coded smartphone screenshots, summing usage of messaging and VoIP apps (WhatsApp, Zoom, etc.)<br><br>*Loneliness:*<br>UCLA Loneliness Scale (Hays & DiMatteo, 1987)	Path 6	Messaging and VoIP apps usage (objectively measured)	FoMO	Loneliness			b = -.001 [-0.00, 0.002], *p* = .001	Correlational	*n* = 334	Young adults<br>*M* = 21.50 (*SD* = 2.03)	n/a
111	Fumagalli et al., 2021	*FoMO:*<br>3 items (Q3, Q6, Q8) of Fear of Missing Out Scale (FoMOs; Przybylski et al., 2013)<br><br>*Social networking apps usage:*<br>Coded smartphone screenshots, summing usage of social network apps	Path 4	Social networking apps usage (objectively measured)	FoMO		.19	***		Correlational	*n* = 334	Young adults<br>*M* = 21.50 (*SD* = 2.03)	n/a

#Ref	Study	Measures	Hypothesized model	IV	DV	M	r	p	Indirect effects (β/ b [CI], SE, *p*)	Study design	Sample size	Participant characteristics	Theory
111	Fumagalli et al., 2021	*FoMO:*<br>3 items (Q3, Q6, Q8) of Fear of Missing Out Scale (FoMOs; Przybylski et al., 2013)<br><br>*Messaging and VoIP apps usage:*<br>Coded smartphone screenshots, summing usage of messaging and VoIP apps (WhatsApp, Zoom, etc.)	Path 4	Messaging and VoIP apps usage (objectively measured)	FoMO		-.03	ns		Correlational	*n* = 334	Young adults<br>*M* = 21.50 (*SD* = 2.03)	n/a
31	Fúster et al., 2017	*FoMO:*<br>Fear of Missing Out Scale (FoMOs; Przybylski et al., 2013)<br><br>*Social network access via the mobile phone:*<br>Social network intensity (SNI) drawn from survey by Salehan & Negahban (2013)	Path 2	FoMO	Social network access via the mobile phone		.26	***		Correlational	*n* = 5280	Social media users aged 13-50y<br>*M* = 15.47 (*SD* = 6.1)	n/a
31	Fúster et al., 2017	*FoMO:*<br>Fear of Missing Out Scale (FoMOs; Przybylski et al., 2013)<br><br>*Social media engagement (SME):*<br>Social Media Engagement Questionnaire (SMEQ; Przybylski et al., 2013)	Path 2	FoMO	SME		.32	***		Correlational	*n* = 5280	Social media users aged 13-50y<br>*M* = 15.47 (*SD* = 6.1)	n/a
31	Fúster et al., 2017	*FoMO:*<br>Fear of Missing Out Scale (FoMOs; Przybylski et al., 2013)<br><br>*Social network intensity (SNI):*<br>Social network intensity (SNI) drawn from survey by Salehan & Negahban (2013)	Path 2	FoMO	SNI		.43	***		Correlational	*n* = 5280	Social media users aged 13-50y<br>*M* = 15.47 (*SD* = 6.1)	n/a
31	Fúster et al., 2017	*FoMO:*<br>Fear of Missing Out Scale (FoMOs; Przybylski et al., 2013)<br><br>*Mobile phone addiction (MPA):*<br>Mobile phone addiction survey (Salehan & Negahban, 2013)	Path 2	FoMO	MPA		.45	***		Correlational	*n* = 5280	Social media users aged 13-50y<br>*M* = 15.47 (*SD* = 6.1)	n/a
31	Fúster et al., 2017	*FoMO:*<br>Fear of Missing Out Scale (FoMOs; Przybylski et al., 2013)<br><br>*Experiences related to mobile phone use (MPU):*<br>Questionnaire on experiences related to mobile phone use (CERM; Beranuy et al., 2009)	Path 2	FoMO	Experiences related to mobile phone use		.57	***		Correlational	*n* = 5280	Social media users aged 13-50y<br>*M* = 15.47 (*SD* = 6.1)	n/a
135	Gugushvili et al., 2020	*FoMO:*<br>Fear of Missing Out Scale (FoMOs; Przybylski et al., 2013)<br><br>*Problematic smartphone use (PSU):*<br>The Estonian Smartphone Addiction Proneness Scale (E-SAPS18; Rozgonjuk et al., 2016)	Path 2	FoMO	Problematic smartphone use		.42	***		Correlational	*n* = 426	Aged 18-56y<br>*M* = 26.74 (*SD* = 8.16)	I-PACE
135	Gugushvili et al., 2020	*FoMO:*<br>Fear of Missing Out Scale (FoMOs; Przybylski et al., 2013)<br><br>*Emotional well-being:*<br>Emotional State Questionnaire (Aluoja et al., 1999; Oopik et al., 2006)	Path 5	FoMO	Emotional well-being		-.42	***		Correlational	*n* = 426	Aged 18-56y<br>*M* = 26.74 (*SD* = 8.16)	I-PACE
82	Hadlington & Scase, 2018	*FoMO:*<br>Fear of Missing Out Scale (FoMOs; Przybylski et al., 2013)<br><br>*Internet addiction:*<br>Online Cognition Scale (OCS; Davis et al., 2002)	Path 2	FoMO	Internet addiction		.63	***		Correlational	*n* = 630	Qualtrics Panel participants aged 18-68y<br>*M* = 41.41 (*SD* = 14.18)	n/a
136	Handa & Ahuja, 2020	*FoMO:*<br>4 items (Q1, Q2, Q3, Q4) of Fear of Missing Out Scale (FoMOs; Przybylski et al., 2013)<br><br>*Smartphone addiction:*<br>18 items of Zhitomirsky-Geffet and Blau (2016)	Path 2	FoMO	Smartphone addiction		.41	***		Correlational	*n* = 240	College going smartphone users aged 18-25y	n/a

#Ref	Study	Measures	Hypothesized model	IV	DV	M	r	p	Indirect effects (β/ b [CI], SE, p)	Study design	Sample size	Participant characteristics	Theory
44	Hayran & Anik, 2021	*Trait-FoMO:* Fear of Missing Out Scale (FoMOs; Przybylski et al., 2013)<br><br>*Actual engagement in virtual activities:* Self-designed question asking participants if and how much they experienced FoMO while trying to keep up several online activities	Path 2	Trait-FoMO	Actual engagement in virtual activities		Wave 1 .18	*		Correlational Two waves (different samples)	Wave 1 *n* = 178	Wave 1 Undergraduate students *M* = 21.35 (*SD* = 1.82)	n/a
44	Hayran & Anik, 2021	*Trait-FoMO:* Fear of Missing Out Scale (FoMOs; Przybylski et al., 2013)<br><br>*Actual engagement in virtual activities:* Self-designed question asking participants if and how much they experienced FoMO while trying to keep up several online activities	Path 2	Trait-FoMO	Actual engagement in virtual activities		Wave 2 .13	*		Correlational Two waves (different samples)	Wave 2 *n* = 215	Wave 2 Undergraduate students *M* = 21.57 (*SD* = 1.56)	n/a
44	Hayran & Anik, 2021	*Trait-FoMO:* Fear of Missing Out Scale (FoMOs; Przybylski et al., 2013)<br><br>*Real-time events:* Self-designed question asking participants if and how much they experienced FoMO while trying to keep up several online activities	Path 2	Trait-FoMO	Real-time events		Wave 1 .30	***		Correlational Two waves (different samples)	Wave 1 *n* = 178	Wave 1 Undergraduate students *M* = 21.35 (*SD* = 1.82)	n/a
44	Hayran & Anik, 2021	*Trait-FoMO:* Fear of Missing Out Scale (FoMOs; Przybylski et al., 2013)<br><br>*Real-time events:* Self-designed question asking participants if and how much they experienced FoMO while trying to keep up several online activities	Path 2	Trait-FoMO	Real-time events		Wave 2 .34	***		Correlational Two waves (different samples)	Wave 2 *n* = 215	Wave 2 Undergraduate students *M* = 21.57 (*SD* = 1.56)	n/a
44	Hayran & Anik, 2021	*Trait-FoMO:* Fear of Missing Out Scale (FoMOs; Przybylski et al., 2013)<br><br>*Virtual gatherings with family and friends:* Self-designed question asking participants if and how much they experienced FoMO while trying to keep up several online activities	Path 2	Trait-FoMO	Virtual gatherings with family and friends		Wave 1 .51	***		Correlational Two waves (different samples)	Wave 1 *n* = 178	Wave 1 Undergraduate students *M* = 21.35 (*SD* = 1.82)	n/a
44	Hayran & Anik, 2021	*Trait-FoMO:* Fear of Missing Out Scale (FoMOs; Przybylski et al., 2013)<br><br>*Virtual gatherings with family and friends:* Self-designed question asking participants if and how much they experienced FoMO while trying to keep up several online activities	Path 2	Trait-FoMO	Virtual gatherings with family and friends		Wave 2 .39	***		Correlational Two waves (different samples)	Wave 2 *n* = 215	Wave 2 Undergraduate students *M* = 21.57 (*SD* = 1.56)	n/a
44	Hayran & Anik, 2021	*Trait-FoMO:* Fear of Missing Out Scale (FoMOs; Przybylski et al., 2013)<br><br>*Others' social media posts:* Self-designed question asking participants if and how much they experienced FoMO while trying to keep up several online activities	Path 2	Trait-FoMO	Others' social media posts		Wave 1 .40	***		Correlational Two waves (different samples)	Wave 1 *n* = 178	Wave 1 Undergraduate students *M* = 21.35 (*SD* = 1.82)	n/a
44	Hayran & Anik, 2021	*Trait-FoMO:* Fear of Missing Out Scale (FoMOs; Przybylski et al., 2013)<br><br>*Others' social media posts:* Self-designed question asking participants if and how much they experienced FoMO while trying to keep up several online activities	Path 2	Trait-FoMO	Others' social media posts		Wave 2 .43	***		Correlational Two waves (different samples)	Wave 2 *n* = 215	Wave 2 Undergraduate students *M* = 21.57 (*SD* = 1.56)	n/a
44	Hayran & Anik, 2021	*Trait-FoMO:* Fear of Missing Out Scale (FoMOs; Przybylski et al., 2013)<br><br>*News on pandemic:* Self-designed question asking participants if and how much they experienced FoMO while trying to keep up several online activities	Path 2	Trait-FoMO	News on pandemic		Wave 1 .28	***		Correlational Two waves (different samples)	Wave 1 *n* = 178	Wave 1 Undergraduate students *M* = 21.35 (*SD* = 1.82)	n/a
44	Hayran & Anik, 2021	*Trait-FoMO:* Fear of Missing Out Scale (FoMOs; Przybylski et al., 2013)<br><br>*News on pandemic:* Self-designed question asking participants if and how much they experienced FoMO while trying to keep up several online activities	Path 2	Trait-FoMO	News on pandemic		Wave 2 .13	ns		Correlational Two waves (different samples)	Wave 2 *n* = 215	Wave 2 Undergraduate students *M* = 21.57 (*SD* = 1.56)	n/a

#Ref	Study	Measures	Hypothesized model	IV	DV	M	r	p	Indirect effects (β/ b [CI], SE, *p*)	Study design	Sample size	Participant characteristics	Theory
44	Hayran & Anik, 2021	State-FoMO:<br>Self-designed question "To what extent do you feel like you are missing out on ongoing virtual activities and experiences that take place in your environment?"<br><br>*Actual engagement in virtual activities:*<br>Self-designed question asking participants if and how much they experienced FoMO while trying to keep up several online activities	Path 2	State-FoMO	Actual engagement in virtual activities		Wave 1<br>.41	***		Correlational<br>Two waves (different samples)	Wave 1<br>*n* = 178	Wave 1<br>Undergraduate students<br>*M* = 21.35 (*SD* = 1.82)	n/a
44	Hayran & Anik, 2021	State-FoMO:<br>Self-designed question "To what extent do you feel like you are missing out on ongoing virtual activities and experiences that take place in your environment?"<br><br>*Actual engagement in virtual activities:*<br>Self-designed question asking participants if and how much they experienced FoMO while trying to keep up several online activities	Path 2	State-FoMO	Actual engagement in virtual activities		Wave 2<br>.38	***		Correlational<br>Two waves (different samples)	Wave 2<br>*n* = 215	Wave 2<br>Undergraduate students<br>*M* = 21.57 (*SD* = 1.56)	n/a
44	Hayran & Anik, 2021	State-FoMO:<br>Self-designed question "To what extent do you feel like you are missing out on ongoing virtual activities and experiences that take place in your environment?"<br><br>*Real-time events:*<br>Self-designed question asking participants if and how much they experienced FoMO while trying to keep up several online activities	Path 2	State-FoMO	Real-time events		Wave 1<br>.30	***		Correlational<br>Two waves (different samples)	Wave 1<br>*n* = 178	Wave 1<br>Undergraduate students<br>*M* = 21.35 (*SD* = 1.82)	n/a
44	Hayran & Anik, 2021	State-FoMO:<br>Self-designed question "To what extent do you feel like you are missing out on ongoing virtual activities and experiences that take place in your environment?"<br><br>*Real-time events:*<br>Self-designed question asking participants if and how much they experienced FoMO while trying to keep up several online activities	Path 2	State-FoMO	Real-time events		Wave 2<br>.37	***		Correlational<br>Two waves (different samples)	Wave 2<br>*n* = 215	Wave 2<br>Undergraduate students<br>*M* = 21.57 (*SD* = 1.56)	n/a
44	Hayran & Anik, 2021	State-FoMO:<br>Self-designed question "To what extent do you feel like you are missing out on ongoing virtual activities and experiences that take place in your environment?"<br><br>*Virtual gatherings with family and friends:*<br>Self-designed question asking participants if and how much they experienced FoMO while trying to keep up several online activities	Path 2	State-FoMO	Virtual gatherings with family and friends		Wave 1<br>.26	**		Correlational<br>Two waves (different samples)	Wave 1<br>*n* = 178	Wave 1<br>Undergraduate students<br>*M* = 21.35 (*SD* = 1.82)	n/a
44	Hayran & Anik, 2021	State-FoMO:<br>Self-designed question "To what extent do you feel like you are missing out on ongoing virtual activities and experiences that take place in your environment?"<br><br>*Virtual gatherings with family and friends:*<br>Self-designed question asking participants if and how much they experienced FoMO while trying to keep up several online activities	Path 2	State-FoMO	Virtual gatherings with family and friends		Wave 2<br>.42	***		Correlational<br>Two waves (different samples)	Wave 2<br>*n* = 215	Wave 2<br>Undergraduate students<br>*M* = 21.57 (*SD* = 1.56)	n/a
44	Hayran & Anik, 2021	State-FoMO:<br>Self-designed question "To what extent do you feel like you are missing out on ongoing virtual activities and experiences that take place in your environment?"<br><br>*Others' social media posts:*<br>Self-designed question asking participants if and how much they experienced FoMO while trying to keep up several online activities	Path 2	State-FoMO	Others' social media posts		Wave 1<br>.35	***		Correlational<br>Two waves (different samples)	Wave 1<br>*n* = 178	Wave 1<br>Undergraduate students<br>*M* = 21.35 (*SD* = 1.82)	n/a
44	Hayran & Anik, 2021	State-FoMO:<br>Self-designed question "To what extent do you feel like you are missing out on ongoing virtual activities and experiences that take place in your environment?"<br><br>*Others' social media posts:*<br>Self-designed question asking participants if and how much they experienced FoMO while trying to keep up several online activities	Path 2	State-FoMO	Others' social media posts		Wave 2<br>.40	***		Correlational<br>Two waves (different samples)	Wave 2<br>*n* = 215	Wave 2<br>Undergraduate students<br>*M* = 21.57 (*SD* = 1.56)	n/a

#Ref	Study	Measures	Hypothesized model	IV	DV	M	r	*p*	Indirect effects (β/ b [CI], SE, *p*)	Study design	Sample size	Participant characteristics	Theory
44	Hayran & Anik, 2021	State-FoMO:<br>Self-designed question "To what extent do you feel like you are missing out on ongoing virtual activities and experiences that take place in your environment?"<br><br>*News on pandemic:*<br>Self-designed question asking participants if and how much they experienced FoMO while trying to keep up several online activities	Path 2	State-FoMO	News on pandemic		Wave 1<br>.19	*		Correlational<br>Two waves (different samples)	Wave 1<br>*n* = 178	Wave 1<br>Undergraduate students<br>*M* = 21.35 (*SD* = 1.82)	n/a
44	Hayran & Anik, 2021	State-FoMO:<br>Self-designed question "To what extent do you feel like you are missing out on ongoing virtual activities and experiences that take place in your environment?"<br><br>*News on pandemic:*<br>Self-designed question asking participants if and how much they experienced FoMO while trying to keep up several online activities	Path 2	State-FoMO	News on pandemic		Wave 2<br>.29	***		Correlational<br>Two waves (different samples)	Wave 2<br>*n* = 215	Wave 2<br>Undergraduate students<br>*M* = 21.57 (*SD* = 1.56)	n/a
93	Hunt et al., 2018	*FoMO:*<br>Fear of Missing Out Scale (FoMOs; Przybylski et al., 2013)<br><br>*Social media use (SMU) objectively measured:*<br>providing screenshots to display usage	Path 2	FoMO (baseline)	SMU objectively measured (in the week following completing FoMOs)		.20	*		Two-wave longitudinal study (one week apart; same sample)	*n* = 143	Undergraduate university students, required to have Facebook, Instagram, and Snapchat accounts, and to own an iPhone	n/a
158	J. Wang et al., 2019	*FoMO:*<br>Fear of Missing Out Scale (FoMOs; Przybylski et al., 2013)<br><br>*Smartphone addiction:*<br>Smartphone Addiction Scale - Short Version (SAS-SV; Kwon et al. 2013)	Path 2	FoMO	Smartphone addiction		.40	***		Correlational	*n* = 794	Middle school students aged 15-19y<br>*M* = 16.80 (*SD* = 0.73)	n/a
41	Koessmeier & Büttner, 2021	*Offline FoMO:*<br>Trait-State Fear of Missing Out Scale (T-SFoMOSC; Wegmann et al. 2017) - Trait-FoMO referred to as offline-FoMO<br><br>*Problematic social media use (PSMU) - loss of control:*<br>Internet addiction scale modified for social networking sites (s-IAT-SNS; Wegmann et al., 2015), with two dimensions loss of control and craving	Path 2	Offline-FoMO	PSMU - Loss of control		.54	***		Correlational	*n* = 329	Social media users<br>*M* = 42.58 (*SD* = 14.75)	UGT
41	Koessmeier & Büttner, 2021	*Offline FoMO:*<br>Trait-State Fear of Missing Out Scale (T-SFoMOSC; Wegmann et al. 2017) - Trait-FoMO referred to as offline-FoMO<br><br>*Problematic social media use (PSMU) - craving:*<br>Internet addiction scale modified for social networking sites (s-IAT-SNS; Wegmann et al., 2015), with two dimensions loss of control and craving	Path 2	Offline-FoMO	PSMU - craving		.56	***		Correlational	*n* = 329	Social media users<br>*M* = 42.58 (*SD* = 14.75)	UGT
41	Koessmeier & Büttner, 2021	*State FoMO:*<br>Trait-State Fear of Missing Out Scale (T-SFoMOSC; Wegmann et al. 2017) - State-FoMO as online-FoMO<br><br>*Problematic social media use (PSMU) - loss of control:*<br>Internet addiction scale modified for social networking sites (s-IAT-SNS; Wegmann et al., 2015), with two dimensions loss of control and craving	Path 2	Online-FoMO	PSMU - Loss of control		.63	***		Correlational	*n* = 329	Social media users<br>*M* = 42.58 (*SD* = 14.75)	UGT
41	Koessmeier & Büttner, 2021	*State FoMO:*<br>Trait-State Fear of Missing Out Scale (T-SFoMOSC; Wegmann et al. 2017) - State-FoMO as online-FoMO<br><br>*Problematic social media use (PSMU) - craving:*<br>Internet addiction scale modified for social networking sites (s-IAT-SNS; Wegmann et al., 2015), with two dimensions loss of control and craving	Path 2	Online-FoMO	PSMU - craving		.63	***		Correlational	*n* = 329	Social media users<br>*M* = 42.58 (*SD* = 14.75)	UGT

#Ref	Study	Measures	Hypothesized model	IV	DV	M	r	*p*	Indirect effects (β/ b [CI], SE, *p*)	Study design	Sample size	Participant characteristics	Theory
137	Lee et al., 2020	*FoMO:*<br>Three subscales of relatedness, autonomy, and competence from Manderin version FoMO scale (Lai et al., 2016)<br><br>*Social networking site (SNS) use:*<br>Self-designed question assessing average time of daily SNS use (min)	Path 2	FoMO Relatedness	SNS use		.13	*		Correlational	*n* = 259	Habitually SNS users<br>*M* = 36.10 (*SD* = 12.40)	SCT
137	Lee et al., 2020	*FoMO:*<br>Three subscales of relatedness, autonomy, and competence from Manderin version FoMO scale (Lai et al., 2016)<br><br>*Social networking site (SNS) use:*<br>Self-designed question assessing average time of daily SNS use (min)	Path 2	FoMO Autonomy	SNS use		.14	*		Correlational	*n* = 259	Habitually SNS users<br>*M* = 36.10 (*SD* = 12.40)	SCT
137	Lee et al., 2020	*FoMO:*<br>Three subscales of relatedness, autonomy, and competence from Manderin version FoMO scale (Lai et al., 2016)<br><br>*Social networking site (SNS) use:*<br>Self-designed question assessing average time of daily SNS use (min)	Path 2	FoMO Competence	SNS use		.12	ns		Correlational	*n* = 259	Habitually SNS users<br>*M* = 36.10 (*SD* = 12.40)	SCT
114	Leung et al., 2021	*FoMO:*<br>Fear of Missing Out Scale (FoMOs; Przybylski et al., 2013)<br><br>*Time spent on social networking sites (SNS):*<br>Self-designed question assessing amount of time spent on SNS per day<br><br>*Depression:*<br>Center for Epidemiologic Studies Depression Scale (CES-D; Radloff,	Path 6	Time spent on SNS	Depression	FoMO			b = .67 [0.33, 1.02], *SE* = 0.18	Correlational	*n* = 180	Undergraduate students aged 18-26y<br>*M* = 21.5 (*SD* = 1.67)	n/a
114	Leung et al., 2021	*FoMO:*<br>Fear of Missing Out Scale (FoMOs; Przybylski et al., 2013)<br><br>*Time spent on social networking sites (SNS):*<br>Self-designed question assessing amount of time spent on SNS per	Path 4	Time spent on SNS	FoMO		.26	***		Correlational	*n* = 180	Undergraduate students aged 18-26y<br>*M* = 21.5 (*SD* = 1.67)	n/a
114	Leung et al., 2021	*FoMO:*<br>Fear of Missing Out Scale (FoMOs; Przybylski et al., 2013)<br><br>*Depression:*<br>Center for Epidemiologic Studies Depression Scale (CES-D; Radloff, 1977)	Path 5	FoMO	Depression		.65	***		Correlational	*n* = 180	Undergraduate students aged 18-26y<br>*M* = 21.5 (*SD* = 1.67)	n/a
83	Li et al., 2020	*FoMO:*<br>Trait-State Fear of Missing Out Scale (T-SFoMOSC; Wegmann et al. 2017)<br><br>*Positive affect:*<br>International Positive and Negative Affect Scale Short-Form (I-PANAS-SF; Thompson, 2007)	Path 1	Positive affect	FoMO		.05	ns		Correlational	*n* = 1164	University students<br>*M* = 20.1 (*SD* = 1.6)	I-PACE
83	Li et al., 2020	*FoMO:*<br>Trait-State Fear of Missing Out Scale (T-SFoMOSC; Wegmann et al. 2017)<br><br>*Positive affect:* International Positive and Negative Affect Scale Short-Form (I-PANAS-SF; Thompson, 2007)	Path 1	Positive affect	Trait-FoMO		-.02	ns		Correlational	*n* = 1164	University students<br>*M* = 20.1 (*SD* = 1.6)	I-PACE
83	Li et al., 2020	*FoMO:*<br>Trait-State Fear of Missing Out Scale (T-SFoMOSC; Wegmann et al. 2017)<br><br>*Positive affect:* International Positive and Negative Affect Scale Short-Form (I-PANAS-SF; Thompson, 2007)	Path 1	Positive affect	State-FoMO		.09	**		Correlational	*n* = 1164	University students<br>*M* = 20.1 (*SD* = 1.6)	I-PACE
83	Li et al., 2020	*FoMO:*<br>Trait-State Fear of Missing Out Scale (T-SFoMOSC; Wegmann et al. 2017)<br><br>*Negative affect:* International Positive and Negative Affect Scale Short-Form (I-PANAS-SF; Thompson, 2007)	Path 1	Negative affect	FoMO		.24	**		Correlational	*n* = 1164	University students<br>*M* = 20.1 (*SD* = 1.6)	I-PACE

#Ref	Study	Measures	Hypothesized model	IV	DV	M	r	*p*	Indirect effects (β/ b [CI], SE, *p*)	Study design	Sample size	Participant characteristics	Theory
83	Li et al., 2020	*FoMO:*<br>Trait-State Fear of Missing Out Scale (T-SFoMOSC; Wegmann et al. 2017)<br><br>*Negative affect:* International Positive and Negative Affect Scale Short-Form (I-PANAS-SF; Thompson, 2007)	Path 1	Negative affect	Trait-FoMO		.26	**		Correlational	*n* = 1164	University students<br>*M* = 20.1 (*SD* = 1.6)	I-PACE
83	Li et al., 2020	*FoMO:*<br>Trait-State Fear of Missing Out Scale (T-SFoMOSC; Wegmann et al. 2017)<br><br>*Negative affect:* International Positive and Negative Affect Scale Short-Form (I-PANAS-SF; Thompson, 2007)	Path 1	Negative affect	State-FoMO		.16	**		Correlational	*n* = 1164	University students<br>*M* = 20.1 (*SD* = 1.6)	I-PACE
83	Li et al., 2020	*FoMO:*<br>Trait-State Fear of Missing Out Scale (T-SFoMOSC; Wegmann et al. 2017)<br><br>*Smartphone addiction:*<br>Mobile Phone Addiction Index (MPAI; Huang, Yang & Li., 2008)	Path 2	FoMO	Smartphone addiction		.45	**		Correlational	*n* = 1164	University students<br>*M* = 20.1 (*SD* = 1.6)	I-PACE
83	Li et al., 2020	*FoMO:*<br>Trait-State Fear of Missing Out Scale (T-SFoMOSC; Wegmann et al. 2017)<br><br>*Smartphone addiction:*<br>Mobile Phone Addiction Index (MPAI; Huang, Yang & Li., 2008)	Path 2	Trait-FoMO	Smartphone addiction		.33	**		Correlational	*n* = 1164	University students<br>*M* = 20.1 (*SD* = 1.6)	I-PACE
83	Li et al., 2020	*FoMO:*<br>Trait-State Fear of Missing Out Scale (T-SFoMOSC; Wegmann et al. 2017)<br><br>*Smartphone addiction:*<br>Mobile Phone Addiction Index (MPAI; Huang, Yang & Li., 2008)	Path 2	State-FoMO	Smartphone addiction		.42	**		Correlational	*n* = 1164	University students<br>*M* = 20.1 (*SD* = 1.6)	I-PACE
55	Lin et al., 2021	*FoMO:*<br>Fear of Missing Out Scale (FoMOs; Przybylski et al., 2013)<br><br>*Interpersonal sensitivity:*<br>Interpersonal Sensitivity Measure (IPSM; Boyce & Parker, 1989)	Path 1	Interpersonal sensitivity	FoMO		.56	**		Correlational	*n* = 881	College students aged 17-23y<br>*M* = 20.93	CIUT
55	Lin et al., 2021	*FoMO:*<br>Fear of Missing Out Scale (FoMOs; Przybylski et al., 2013)<br><br>*Mobile phone addiction (MPA):*<br>Smartphone Addiction Scale for College Students (SAS-C; Su et al., 2014)	Path 2	FoMO	MPA		.45	**		Correlational	*n* = 881	College students aged 17-23y<br>*M* = 20.93	CIUT
140	Liu & Ma, 2020	*FoMO:*<br>Fear of Missing Out Scale (FoMOs; Przybylski et al., 2013)<br><br>*Problematic smartphone use (PSU):*<br>Smartphone Addiction Scale-Short Version (SAS-SV; Kwon et al., 2013)	Path 2	FoMO	PSU		.36	**		Correlational	*n* = 465	Undergraduates colleges students with at least six months 'experience of social media use aged 16-24y<br>*M* = 18.83 (*SD* = 1.08)	SCT
140	Liu & Ma, 2020	*FoMO:*<br>Fear of Missing Out Scale (FoMOs; Przybylski et al., 2013)<br><br>*Social networking site (SNS) addiction:*<br>Social media addiction scale (Liu & Ma., 2018)	Path 2	FoMO	SNS addiction		.40	**		Correlational	*n* = 465	Undergraduates colleges students with at least six months 'experience of social media use aged 16-24y<br>*M* = 18.83 (*SD* = 1.08)	SCT
29	Lo Coco et al., 2020	*FoMO:*<br>Fear of Missing Out Scale (FoMOs; Przybylski et al., 2013) (Covers two domains: FoMO-Fear and FoMO-Control)<br><br>*Problematic smartphone use (PSU):*<br>Smartphone Addiction Scale-short version (SAS-SV; Kwon et al.,	*No path specified*	FoMO Fear T0	PSU T0		.38	**		Two-wave longitudinal study (one year apart; same sample)	*n* = 242 (participants who completed both questionnaire	Adolescents aged 12-16y<br>*M* = 14.16 (*SD* = 0.99)	CIUT, and, SDT
29	Lo Coco et al., 2020	*FoMO:*<br>Fear of Missing Out Scale (FoMOs; Przybylski et al., 2013) (Covers two domains: FoMO-Fear and FoMO-Control)<br><br>*Problematic smartphone use (PSU):*<br>Smartphone Addiction Scale-short version (SAS-SV; Kwon et al.,	*No path specified*	FoMO Control T0	PSU T0		.44	**		Two-wave longitudinal study (one year apart; same sample)	*n* = 242 (participants who completed both questionnaire	Adolescents aged 12-16y<br>*M* = 14.16 (*SD* = 0.99)	CIUT, and, SDT

#Ref	Study	Measures	Hypothesized model	IV	DV	M	r	*p*	Indirect effects (β/ b [CI], SE, *p*)	Study design	Sample size	Participant characteristics	Theory
29	Lo Coco et al., 2020	*FoMO:*<br>Fear of Missing Out Scale (FoMOs; Przybylski et al., 2013) (Covers two domains: FoMO-Fear and FoMO-Control)<br><br>*Problematic smartphone use (PSU):*<br>Smartphone Addiction Scale-short version (SAS-SV; Kwon et al.,	*No path specified*	FoMO Control T1	PSU T0		.17	*		Two-wave longitudinal study (one year apart; same sample)	*n* = 242 (participants who completed both questionnaire	Adolescents aged 12-16y<br>*M* = 14.16 (*SD* = 0.99)	CIUT, and, SDT
29	Lo Coco et al., 2020	*FoMO:*<br>Fear of Missing Out Scale (FoMOs; Przybylski et al., 2013) (Covers two domains: FoMO-Fear and FoMO-Control)<br><br>*Problematic smartphone use (PSU):*<br>Smartphone Addiction Scale-short version (SAS-SV; Kwon et al.,	*No path specified*	FoMO Fear T1	PSU T0		.06	ns		Two-wave longitudinal study (one year apart; same sample)	*n* = 242 (participants who completed both questionnaire	Adolescents aged 12-16y,<br>*M* = 14.16 (*SD* = 0.99)	CIUT, and, SDT
29	Lo Coco et al., 2020	*FoMO:*<br>Fear of Missing Out Scale (FoMOs; Przybylski et al., 2013) (Covers two domains: FoMO-Fear and FoMO-Control)<br><br>*Problematic smartphone use (PSU):*<br>Smartphone Addiction Scale-short version (SAS-SV; Kwon et al.,	*No path specified*	FoMO Fear T0	PSU T1		.14	*		Two-wave longitudinal study (one year apart; same sample)	*n* = 242 (participants who completed both questionnaire	Adolescents aged 12-16y,<br>*M* = 14.16 (*SD* = 0.99)	CIUT, and, SDT
29	Lo Coco et al., 2020	*FoMO:*<br>Fear of Missing Out Scale (FoMOs; Przybylski et al., 2013) (Covers two domains: FoMO-Fear and FoMO-Control)<br><br>*Problematic smartphone use (PSU):*<br>Smartphone Addiction Scale-short version (SAS-SV; Kwon et al.,	*No path specified*	FoMO Control T0	PSU T1		.21	**		Two-wave longitudinal study (one year apart; same sample)	*n* = 242 (participants who completed both questionnaire	Adolescents aged 12-16y,<br>*M* = 14.16 (*SD* = 0.99)	CIUT, and, SDT
29	Lo Coco et al., 2020	*FoMO:*<br>Fear of Missing Out Scale (FoMOs; Przybylski et al., 2013) (Covers two domains: FoMO-Fear and FoMO-Control)<br><br>*Problematic smartphone use (PSU):*<br>Smartphone Addiction Scale-short version (SAS-SV; Kwon et al.,	*No path specified*	FoMO Fear T1	PSU T1		.39	**		Two-wave longitudinal study (one year apart; same sample)	*n* = 242 (participants who completed both questionnaire	Adolescents aged 12-16y,<br>*M* = 14.16 (*SD* = 0.99)	CIUT, and, SDT
29	Lo Coco et al., 2020	*FoMO:*<br>Fear of Missing Out Scale (FoMOs; Przybylski et al., 2013) (Covers two domains: FoMO-Fear and FoMO-Control)<br><br>*Problematic smartphone use (PSU):*<br>Smartphone Addiction Scale-short version (SAS-SV; Kwon et al.,	*No path specified*	FoMO Control T1	PSU T1		.41	**		Two-wave longitudinal study (one year apart; same sample)	*n* = 242 (participants who completed both questionnaire	Adolescents aged 12-16y,<br>*M* = 14.16 (*SD* = 0.99)	CIUT, and, SDT
141	Marengo et al., 2021	*FoMO:*<br>Fear of Missing Out Scale (FoMOs; Przybylski et al., 2013)<br><br>*Exclusion from WhatsApp classmate groups:*<br>Adapting items from Aizenkot & Kashy-Rosenbaum (2019)<br><br>*Emotional symptoms:*<br>Strengths and Difficulties Questionnaire (SDQ; Goodman et al.,	Path 6	Exclusion from WhatsApp classmate groups	Emotional symptoms	FoMO			.08 [0.05, 0.16]	Correlational	*n* = 398	Middle schoolers aged 11-15y<br>*M* = 12.54 (*SD* = .96)	n/a
141	Marengo et al., 2021	*FoMO:*<br>Fear of Missing Out Scale (FoMOs; Przybylski et al., 2013)<br><br>*Exclusion from WhatsApp classmate groups:*<br>Adapting items from Aizenkot & Kashy-Rosenbaum (2019)	Path 4	Exclusion from WhatsApp classmate groups	FoMO		.29	**		Correlational	*n* = 398	Middle schoolers aged 11-15y<br>*M* = 12.54 (*SD* = .96)	n/a
141	Marengo et al., 2021	*FoMO:*<br>Fear of Missing Out Scale (FoMOs; Przybylski et al., 2013)<br><br>*Emotional symptoms:*<br>Strengths and Difficulties Questionnaire (SDQ; Goodman et al.,	Path 5	FoMO	Emotional symptoms		.42	**		Correlational	*n* = 398	Middle schoolers aged 11-15y<br>*M* = 12.54 (*SD* = .96)	n/a
28	Milyavskaya et al., 2018	Study 1:<br>*FoMO (more frequently during the day):*<br>At each signal; "Is there something your friends or peers are doing right now, that you feel you are missing out on?" (yes/ no response option)<br><br>*Stress (over the semester):*<br>Self-designed 3-item questionnaire measuring 'academic/ school stress', 'interpersonal stress', and 'general stress', combined to measure overall stress	Path 5	FoMO	Stress		.31	**		Mixed method study<br>*Study 1 longitudinal experiences sampling (nightly diaries and end-of-semester measures)*	*n* = 151	College freshmen<br>*M* = 18.0 (*SD* = 1.04)	Economic Rational Choice Theory

#Ref	Study	Measures	Hypothesized model	IV	DV	M	r	*p*	Indirect effects (β/ b [CI], SE, *p*)	Study design	Sample size	Participant characteristics	Theory
28	Milyavskaya et al., 2018	FoMO:<br>"How strongly would you feel that you were missing out on the second option?" (definition of Przybylski et al. (2013))<br><br>*Affect:*<br>At the fixed evening signal, and at the start of the semester. Participants rated affect during the day using a 9 item scale of affect (Emmons, 1992) asking how much they would have felt each emotion in the imagined scenario	Path 5	FoMO	Negative affect		.45	***		Mixed method study<br>*Study 2 causal/ experiment (vignette study)*	*n* = 268	Mturk adults aged 18-72y<br>*M* = 30.8 (*SD* = 11.8)	Economic Rational Choice Theory
28	Milyavskaya et al., 2018	FoMO:<br>"How strongly would you feel that you were missing out on the second option?" (definition of Przybylski et al. (2013))<br><br>*Affect:*<br>At the fixed evening signal, and at the start of the semester. Participants rated affect during the day using a 9 item scale of affect (Emmons, 1992) asking how much they would have felt each emotion in the imagined scenario	Path 5	FoMO	Positive affect		-.30	***		Mixed method study<br>*Study 2 causal/ experiment (vignette study)*	*n* = 268	Mturk adults aged 18-72y<br>*M* = 30.8 (*SD* = 11.8)	Economic Rational Choice Theory
125	Moore & Crăciun, 2021	*FoMO:*<br>Fear of Missing Out Scale (FoMOs; Przybylski et al., 2013)<br><br>*Attitude towards Instagram:*<br>Attitude toward Social Media Scale (Pittman & Reich, 2016)	Path 2	FoMO	Attitude towards Instagram		.37	**		Correlational	*n* = 156	Undergraduate students<br>Median = 20y	n/a
125	Moore & Crăciun, 2021	*FoMO:*<br>Fear of Missing Out Scale (FoMOs; Przybylski et al., 2013)<br><br>*Number of accounts followed on Instagram:*<br>Objective data retrieved from user's Instagram profile page	Path 2	FoMO	Number of accounts followed on Instagram		.23	*		Correlational	*n* = 156	Undergraduate students<br>Median = 20y	n/a
125	Moore & Crăciun, 2021	*FoMO:*<br>Fear of Missing Out Scale (FoMOs; Przybylski et al., 2013)<br><br>*Frequency of posting on Instagram:*<br>Objective data retrieved from user's Instagram profile page	Path 2	FoMO	Frequency of posting on Instagram		.14	ns		Correlational	*n* = 156	Undergraduate students<br>Median = 20y	n/a
125	Moore & Crăciun, 2021	*FoMO:*<br>Fear of Missing Out Scale (FoMOs; Przybylski et al., 2013)<br><br>*Social media addictive tendencies:*<br>The Addictive Tendencies Scale (Walsh et al., 2017)	Path 2	FoMO	Social media addictive tendencies		.43	**		Correlational	*n* = 156	Undergraduate students<br>Median = 20y	n/a
142	Müller et al., 2020	*State-FoMO:*<br>State subscale of the Trait-State Fear of Missing Out Scale (T-SFoMOSC; Wegmann et al. 2017)<br><br>*Problematic social networking working (SNS) use:*<br>SNS-specific short Internet Addiction Test (sIAT_SNS; Wegmann, Stodt & Brand, 2015)	Path 2	State-FoMO	Problematic SNS use		.50	**		Correlational	*n* = 226	Individuals aged 17-37y<br>*M* = 22.00 (*SD* = 3.30)	n/a
52	Oberst et al., 2016	*FoMO:*<br>Fear of Missing Out Scale (FoMOs; Przybylski et al., 2013)<br><br>*Depression:*<br>Hospital Anxiety and Depression Scale (HADS; Quintana et al., 2003)	Path 1	Depression	FoMO		.19	***		Correlational	*n* = 1468	Social media users aged 16-18y<br>*M* = 16.59 (*SD* = 0.62)	SDT
52	Oberst et al., 2016	*FoMO:*<br>Fear of Missing Out Scale (FoMOs; Przybylski et al., 2013)<br><br>*Anxiety:*<br>Hospital Anxiety and Depression Scale (HADS; Quintana et al., 2003)	Path 1	Anxiety	FoMO		.41	***		Correlational	*n* = 1468	Social media users aged 16-18y<br>*M* = 16.59 (*SD* = 0.62)	SDT
52	Oberst et al., 2016	*FoMO:*<br>Fear of Missing Out Scale (FoMOs; Przybylski et al., 2013)<br><br>*Social network intensity (SNI):*<br>Self-designed 5 item scale drawn from a survey by Salehan and Negahban (2013)	Path 2	FoMO	SNI		.46	***		Correlational	*n* = 1468	Social media users aged 16-18y<br>*M* = 16.59 (*SD* = 0.62)	SDT

#Ref	Study	Measures	Hypothesized model	IV	DV	M	r	*p*	Indirect effects (β/ b [CI], SE, *p*)	Study design	Sample size	Participant characteristics	Theory
16	Orta, 2020	*FoMO:*<br>Fear of Missing Out Scale (FoMOs; Przybylski et al., 2013)<br><br>*Internet addiction:*<br>Internet Addiction Scale (Young, 1998)	Path 2	FoMO	Internet addiction		.33	**		Correlational	*n* = 322	Undergraduate students<br>*M* = 22.16 (*SD* = 2.72)	CIUT
16	Orta, 2020	*FoMO:*<br>Fear of Missing Out Scale (FoMOs; Przybylski et al., 2013)<br><br>*Psychological problems:*<br>Brief Symptom Inventory (Derogatis, 1977)	Path 5	FoMO	Psychological problems		.36	**		Correlational	*n* = 322	Undergraduate students<br>*M* = 22.16 (*SD* = 2.72)	CIUT
63	P. Wang et al., 2019	*FoMO:*<br>Fear of Missing Out Scale (FoMOs; Przybylski et al., 2013)<br><br>*Problematic smartphone use (PSU):*<br>Smartphone Addiction Scale (SAS-SV ; Kwon et al., 2013)	Path 2	FoMO	PSU		.40	***		Correlational	*n* = 724	Middle school students<br>*M* = 16.79 (*SD* = 0.91)	n/a
143	Patel et al., 2021	*FoMO:*<br>Fear of Missing Out Scale (FoMOs; Przybylski et al., 2013)<br><br>*Social media addiction (SMA):*<br>The Social Media Disorder Scale (Regina et al., 2016)	*No path specified*	FoMO	SMA		.46	***		Correlational	*n* = 712	Undergraduate health professionals<br>*M* = 21.4 (*SD* = 2.15)	n/a
144	Pontes et al., 2018	*FoMO:*<br>Fear of Missing Out Scale (FoMOs; Przybylski et al., 2013)<br><br>*Problems due to social networking site (SNS) use:* Self-designed yes/ no question	*No path specified*	FoMO	Problems due to SNS use		.31	**		Correlational	*n* = 532	Social network site users<br>13.3% adolescents (16–19y),<br>58.1% young adults (20–35y),<br>28.6% were adults (>36y)	n/a
144	Pontes et al., 2018	*FoMO:*<br>Fear of Missing Out Scale (FoMOs; Przybylski et al., 2013)<br><br>*Preference for online social interaction:*<br>Subscale from the Generalized Problematic Internet Use Scale-2 (Caplan, 2010)	*No path specified*	FoMO	Preference for online social interaction		.44	**		Correlational	*n* = 532	Social network site users<br>13.3% adolescents (16–19y),<br>58.1% young adults (20–35y),<br>28.6% were adults (>36y)	n/a
144	Pontes et al., 2018	*FoMO:*<br>Fear of Missing Out Scale (FoMOs; Przybylski et al., 2013)<br><br>*Pschiatric distress:*<br>Symptom Checklist-6 (Rosen et al., 2000)	*No path specified*	FoMO	Pschiatric distress		.46	**		Correlational	*n* = 532	Social network site users<br>13.3% adolescents (16–19y),<br>58.1% young adults (20–35y),<br>28.6% were adults (>36y)	n/a
144	Pontes et al., 2018	*FoMO:*<br>Fear of Missing Out Scale (FoMOs; Przybylski et al., 2013)<br><br>*Dysfunctional emotion regulation:*<br>Difficulties in Emotion Regulation Scale-Short Form (Kaufman et al., 2016)	*No path specified*	FoMO	Dysfunctional emotion regulation		.62	**		Correlational	*n* = 532	Social network site users<br>13.3% adolescents (16–19y),<br>58.1% young adults (20–35y),<br>28.6% were adults (>36y)	n/a
144	Pontes et al., 2018	*FoMO:*<br>Fear of Missing Out Scale (FoMOs; Przybylski et al., 2013)<br><br>*Social networking site (SNS) addiction:*<br>Bergen Social Media Addiction Scale (BSMAS; Andreassen et al., 2017)	*No path specified*	FoMO	SNS addiction		.68	**		Correlational	*n* = 532	Social network site users<br>13.3% adolescents (16–19y),<br>58.1% young adults (20–35y),<br>28.6% were adults (>36y)	n/a
5	Przybylski et al., 2013	*FoMO:*<br>Fear of Missing Out Scale (FoMOs; Przybylski et al., 2013)<br><br>*Life satisfaction (LS):*<br>Self-designed questionnaire assessing LS across four areas 1) physical health, 2) emotional health, 3) personal relationships, and 4) life as a whole	Path 1	LS	FoMO		-.19	***		Correlational	Study 2<br>*n* = 2079	Working age adults aged 22-65y<br>*M* = 43.21 (*SD* = 11.49)	SDT
5	Przybylski et al., 2013	*FoMO:*<br>Fear of Missing Out Scale (FoMOs; Przybylski et al., 2013)<br><br>*General mood:*<br>Adapted nine-item version of the Emmons Mood Indicator (Diener & Emmons, 1984)	Path 1	General mood	FoMO		-.24	***		Correlational	Study 2<br>*n* = 2079	Working age adults aged 22-65y<br>*M* = 43.21 (*SD* = 11.49)	SDT

#Ref	Study	Measures	Hypothesized model	IV	DV	M	r	*p*	Indirect effects (β/ b [CI], SE, *p*)	Study design	Sample size	Participant characteristics	Theory
5	Przybylski et al., 2013	*FoMO:*<br>Fear of Missing Out Scale (FoMOs; Przybylski et al., 2013)<br><br>*Psychological need satisfaction (PNS):*<br>Individual difference version of the Need Satisfaction Scale (La Guardia et al., 2000)	Path 1	PNS	FoMO		-.29	***		Correlational	Study 2<br>*n* = 2079	Working age adults aged 22-65y<br>*M* = 43.21 (*SD* = 11.49)	SDT
5	Przybylski et al., 2013	*FoMO:*<br>Fear of Missing Out Scale (FoMOs; Przybylski et al., 2013)<br><br>*Social media engagement (SME):*<br>*Self-designed questionnaire assessing the extent to which participants used social media in their daily lives (e.g.,* within 15 min of waking up, within 15 min of going to sleep, etc.)	Path 2	FoMO	SME		.40	***		Correlational	Study 2<br>*n* = 2079	Working age adults aged 22-65y<br>*M* = 43.21 (*SD* = 11.49)	SDT
5	Przybylski et al., 2013	*FoMO:*<br>Fear of Missing Out Scale (FoMOs; Przybylski et al., 2013)<br><br>*Social media engagement (SME):*<br>Self-designed questionnaire assessing the extent to which participants used social media in their daily lives (e.g., within 15 min of waking up, within 15 min of going to sleep, etc.)<br><br>*Psychological need satisfaction (PNS):*<br>Individual difference version of the Need Satisfaction Scale (La Guardia et al., 2000)	Path 3	PNS	SME	FoMO			[-0.65, -0.100]	Correlational	Study 2<br>*n* = 2079	Working age adults aged 22-65y<br>*M* = 43.21 (*SD* = 11.49)	SDT
5	Przybylski et al., 2013	*FoMO:*<br>Fear of Missing Out Scale (FoMOs; Przybylski et al., 2013)<br><br>*Social media engagement (SME):*<br>Self-designed questionnaire assessing the extent to which participants used social media in their daily lives (e.g., within 15 min of waking up, within 15 min of going to sleep, etc.).<br><br>*Life satisfaction (LS):*<br>Measured with an assessment that tapped into LS across four areas (physical health, emotional health, personal relationships, and life as a whole)	Path 3	LS	SME	FoMO			[-0.36, -0.61]	Correlational	Study 2<br>*n* = 2079	Working age adults aged 22-65y<br>*M* = 43.21 (*SD* = 11.49)	SDT
5	Przybylski et al., 2013	*FoMO:*<br>Fear of Missing Out Scale (FoMOs; Przybylski et al., 2013)<br><br>*Social media engagement (SME):*<br>Self-designed questionnaire assessing the extent to which participants used social media in their daily lives (e.g., within 15 min of waking up, within 15 min of going to sleep, etc.).<br><br>*General mood:*<br>Adapted nine-item version of the Emmons Mood Indicator (Diener & Emmons, 1984)	Path 3	General mood	SME	FoMO			[-0.57, -0.99]	Correlational	Study 2<br>*n* = 2079	Working age adults aged 22-65y<br>*M* = 43.21 (*SD* = 11.49)	SDT
145	Quaglieri et al., 2021	*FoMO:*<br>Fear of Missing Out Scale (FoMOs; Przybylski et al., 2013)<br><br>*Internet addiction:*<br>The Internet Addiction Test (IAT; Young, 1998)	*No path specified*	FoMO	Internet addiction		.53	**		Correlational	*n* = 397	Young adults aged 18-35y<br>*M* = 22 (*SD* = 3.83)	n/a
145	Quaglieri et al., 2021	*FoMO:*<br>Fear of Missing Out Scale (FoMOs; Przybylski et al., 2013)<br><br>*Social media addiction (SMA):* Bergen Social Media Addiction Scale (BSMAS; Andreassen et al., 2016)	*No path specified*	FoMO	SMA		.49	**		Correlational	*n* = 397	Young adults aged 18-35y<br>*M* = 22 (*SD* = 3.83)	n/a
35	Qutishat & Sharour, 2019	*FoMO:*<br>Fear of Missing Out Scale (FoMOs; Przybylski et al., 2013)<br><br>*Notifications in class:*<br>Self-designed questionnaire including notification in class (e.g., ignore the notification until class ends, send a message saying 'in	Path 4	Notifications in class	FoMO			ns		Correlational	*n* = 147	Undergraduate University students with at least one smartphone device that was continuously connected to the internet aged 18-22y<br>*M* = 21.00	n/a

#Ref	Study	Measures	Hypothesized model	IV	DV	M	r	*p*	Indirect effects (β/ b [CI], SE, *p*)	Study design	Sample size	Participant characteristics	Theory
35	Qutishat & Sharour, 2019	*FoMO:* Fear of Missing Out Scale (FoMOs; Przybylski et al., 2013)<br><br>*Number of Smartphones:*<br>Self-designed questionnaire including number of smartphones	Path 4	Number of smartphones	FoMO			ns		Correlational	*n* = 147	Undergraduate University students with at least one smartphone device that was continuously connected to the internet aged 18-22y<br>*M* = 21.00	n/a
35	Qutishat & Sharour, 2019	*FoMO:*<br>Fear of Missing Out Scale (FoMOs; Przybylski et al., 2013)<br><br>*Purpose in class:*<br>Self-designed questionnaire including purpose use in classroom (study, work, chatting, etc.)	Path 4	Purpose use in class	FoMO			ns		Correlational	*n* = 147	Undergraduate University students with at least one smartphone device that was continuously connected to the internet aged 18-22y<br>*M* = 21.00	n/a
35	Qutishat & Sharour, 2019	*FoMO:*<br>Fear of Missing Out Scale (FoMOs; Przybylski et al., 2013)<br><br>*Smartphone status:*<br>Self-designed questionnaire including smartphone status (loud, silence, vibrate, off)	Path 4	Smartphone status	FoMO			ns		Correlational	*n* = 147	Undergraduate University students with at least one smartphone device that was continuously connected to the internet aged 18-22y<br>*M* = 21.00	n/a
14	Radić et al., 2020	*FoMO:*<br>Fear of missing out on important events and information when not using the Internet scale (Reinecke et al., 2016)<br><br>*Internet multitasking:*<br>Communication load and Internet Multitasking scale (Reinecke et al., 2016)	Path 4	Internet multitasking	FoMO		.30	***		Correlational	*n* = 532	Cruise ship employees aged 21-50y	CIUT
62	Reer et al., 2019	*FoMO:*<br>Fear of Missing Out Scale (FoMOs; Przybylski et al., 2013)<br><br>*Social media engagement (SME):*<br>Social Media Engagement Questionnaire (SMEQ; Przybylski et al. 2013)<br><br>*Social comparison orientation (SCO):*<br>Lowa-Netherlands Comparison Orientation Measure (Gibbons & Buunk, 1999)<br><br>*Anxiety:*<br>Patient Health Questionnaire-4 (PHQ-4; Kroenke et al., 2009)	Path 3	Anxiety	SME	SCO > FoMO			.09 [0.07, 0.11], *p* < .001	Correlational	*n* = 1865	Internet users aged 14-39y<br>*M* = 27.65 (*SD* = 6.85)	SCT
62	Reer et al., 2019	*FoMO:*<br>Fear of Missing Out Scale (FoMOs; Przybylski et al., 2013)<br><br>*Social media engagement (SME):*<br>Social Media Engagement Questionnaire (SMEQ; Przybylski et al. 2013)<br><br>*Social comparison orientation (SCO):*<br>Lowa-Netherlands Comparison Orientation Measure (Gibbons & Buunk, 1999)<br><br>*Depression:*<br>Patient Health Questionnaire-4 (PHQ-4; Kroenke et al., 2009)	Path 3	Depression	SME	SCO > FoMO			.09 [0.07, 0.11], *p* < .001	Correlational	*n* = 1865	Internet users aged 14-39y<br>*M* = 27.65 (*SD* = 6.85)	SCT
62	Reer et al., 2019	*FoMO:*<br>Fear of Missing Out Scale (FoMOs; Przybylski et al., 2013)<br><br>*Social media engagement (SME):*<br>Social Media Engagement Questionnaire (SMEQ; Przybylski et al. 2013)<br><br>*Social comparison orientation (SCO):*<br>Lowa-Netherlands Comparison Orientation Measure (Gibbons & Buunk, 1999)<br><br>*Loneliness:* Loneliness Short Scale (Hughes et al.,2004)	Path 3	Loneliness	SME	SCO > FoMO			.10 [0.08, 0.12], *p* < .001	Correlational	*n* = 1865	Internet users aged 14-39y<br>*M* = 27.65 (*SD* = 6.85)	SCT

#Ref	Study	Measures	Hypothesized model	IV	DV	M	r	*p*	Indirect effects (β/ b [CI], SE, *p*)	Study design	Sample size	Participant characteristics	Theory
62	Reer et al., 2019	*FoMO:*<br>Fear of Missing Out Scale (FoMOs; Przybylski et al., 2013)<br><br>*Social media engagement (SME):*<br>Social Media Engagement Questionnaire (SMEQ; Przybylski et al. 2013)	Path 2	FoMO	SME		.30	**		Correlational	*n* = 1865	Internet users aged 14-39y<br>*M* = 27.65 (*SD* = 6.85)	SCT
62	Reer et al., 2019	*FoMO:*<br>Fear of Missing Out Scale (FoMOs; Przybylski et al., 2013)<br><br>*Loneliness:*<br>Loneliness short scale (Hughes et al., 2004)	Path 1	Loneliness	FoMO		.31	**		Correlational	*n* = 1865	Internet users aged 14-39y<br>*M* = 27.65 (*SD* = 6.85)	SCT
62	Reer et al., 2019	*FoMO:*<br>Fear of Missing Out Scale (FoMOs; Przybylski et al., 2013)<br><br>*Anxiety:*<br>Patient Health Questionnaire-4 (PHQ-4; Kroenke et al., 2009)	Path 1	Anxiety	FoMO		.37	**		Correlational	*n* = 1865	Internet users aged 14-39y<br>*M* = 27.65 (*SD* = 6.85)	SCT
62	Reer et al., 2019	*FoMO:*<br>Fear of Missing Out Scale (FoMOs; Przybylski et al., 2013)<br><br>*Depression:*<br>Patient Health Questionnaire-4 (PHQ-4; Kroenke et al., 2009)	Path 1	Depression	FoMO		.39	**		Correlational	*n* = 1865	Internet users aged 14-39y<br>*M* = 27.65 (*SD* = 6.85)	SCT
62	Reer et al., 2019	*FoMO:*<br>Fear of Missing Out Scale (FoMOs; Przybylski et al., 2013)<br><br>*Social comparison orientation (SCO):*<br>Lowa-Netherlands Comparison Orientation Measure (Gibbons & Buunk, 1999)	Path 1	SCO	FoMO		.52	**		Correlational	*n* = 1865	Internet users aged 14-39y<br>*M* = 27.65 (*SD* = 6.85)	SCT
146	Reyes et al., 2018	*FoMO:*<br>Fear of Missing Out Scale (FoMOs; Przybylski et al., 2013)<br><br>*Social networking time:*<br>Social Networking Time Use Scale (SONTUS; Olufadi, 2016)	Path 2	FoMO	Social networking time		.41	**		Correlational	*n* = 1060	Individuals aged 18-65y<br>*M* = 25.22 (*SD* = 9.83)	SDT
146	Reyes et al., 2018	*FoMO:*<br>Fear of Missing Out Scale (FoMOs; Przybylski et al., 2013)<br><br>*Problematic internet use (PIU):*<br>Internet Addiction Test (IAT; Young, 1998)	Path 2	FoMO	PIU		.57	**		Correlational	*n* = 1060	Individuals aged 18-65y<br>*M* = 25.22 (*SD* = 9.83)	SDT
86	Riordan et al., 2018	*FoMO:*<br>Fear of Missing Out Scale (FoMOs; Przybylski et al., 2013)<br><br>*Facebook engagement:*<br>Social Media Engagement Questionnaire (SMEQ; Przybylski et al. 2013)	Path 2	FoMO	Facebook engagement		.28	***		Correlational	*n* = 330	University students aged 17-40y<br>*M* = 19.6 (*SD* = 2.2)	n/a
94	Rogers & Barber, 2019	*FoMO:*<br>Fear of Missing Out Scale (FoMOs; Przybylski et al., 2013)<br><br>*Technology engagement before sleep:*<br>"Please reflect on how you used information/ communication devices over the past week and report the number of times you used it within 15 min of going to sleep" (Barber & Cucalon, 2017)	Path 2	FoMO	Technology engagement before sleep (pre intervention)		-.01	ns		Two-wave study (measures pre and post a sleep intervention; 1 year apart)	*n* = 97 pre intervention	University students<br>*M* = 19.81 (*SD* = 2.55)	n/a
94	Rogers & Barber, 2019	*FoMO:*<br>Fear of Missing Out Scale (FoMOs; Przybylski et al., 2013)<br><br>*Technology engagement before sleep:*<br>"Please reflect on how you used information/ communication devices over the past week and report the number of times you used it within 15 min of going to sleep" (Barber & Cucalon, 2017)	Path 2	FoMO	Technology engagement before sleep (post intervention)		.06	ns		Two-wave study (measures pre and post a sleep intervention; 1 year apart)	*n* = 94 post intervention	University students<br>*M* = 19.81 (*SD* = 2.55)	n/a

#Ref	Study	Measures	Hypothesized model	IV	DV	M	r	*p*	Indirect effects (β/ b [CI], SE, *p*)	Study design	Sample size	Participant characteristics	Theory
94	Rogers & Barber, 2019	*FoMO:*<br>Fear of Missing Out Scale (FoMOs; Przybylski et al., 2013)<br><br>*Technology engagement during sleep:*<br>"Please reflect on how you used information/ communication devices over the past week and report the number of times you used it during sleep (e.g., waking up to respond to a text or phone call)" (Barber & Cucalon, 2017)	Path 2	FoMO	Technology engagement during sleep (pre intervention)		.19	ns		Two-wave study (measures pre and post a sleep intervention; 1 year apart)	*n* = 97 pre intervention	University students<br>*M* = 19.81 (*SD* = 2.55)	n/a
94	Rogers & Barber, 2019	*FoMO:*<br>Fear of Missing Out Scale (FoMOs; Przybylski et al., 2013)<br><br>*Technology engagement during sleep:*<br>"Please reflect on how you used information/ communication devices over the past week and report the number of times you used it during sleep (e.g., waking up to respond to a text or phone call)" (Barber & Cucalon, 2017)	Path 2	FoMO	Technology engagement during sleep (post intervention)		.08	ns		Two-wave study (measures pre and post a sleep intervention; 1 year apart)	*n* = 94 post intervention	University students<br>*M* = 19.81 (*SD* = 2.55)	n/a
94	Rogers & Barber, 2019	*FoMO:*<br>Fear of Missing Out Scale (FoMOs; Przybylski et al., 2013)<br><br>*Social media use (SMU):*<br>Self-designed item measuring how often participants use social media platforms	Path 2	FoMO	SMU (pre intervention)		.21	**		Two-wave study (measures pre and post a sleep intervention; 1 year apart)	*n* = 97 pre intervention	University students<br>*M* = 19.81 (*SD* = 2.55)	n/a
94	Rogers & Barber, 2019	*FoMO:*<br>Fear of Missing Out Scale (FoMOs; Przybylski et al., 2013)<br><br>*Social media use (SMU):*<br>Self-designed item measuring how often participants use social media platforms	Path 2	FoMO	SMU (post intervention)		.11	ns		Two-wave study (measures pre and post a sleep intervention; 1 year apart)	*n* = 94 post intervention	University students<br>*M* = 19.81 (*SD* = 2.55)	n/a
147	Röttinger et al., 2021	*FoMO:*<br>Trait-State Fear of Missing Out Scale (T-SFoMOSC; Wegmann et al. 2017)<br><br>*Compulsive internet use (CIU):*<br>Compulsive Internet Use Scale (CIUS; Meerkerk et al., 2009)<br><br>*Psychopathological symptoms:*<br>Mental Health Inventory (MHI-5; Berwick et al., 1991)	Path 3	Psychopathological symptoms	CIU	Trait-FoMO			Male:<br>-.043, *SE* = 0.011, *p* < .001<br><br>Female:<br>-.029, *SE* = 0.017, *p* = ns	Correlational	*n* = 4973 (subsample of social media users)	Students at vocational schools aged 16-54y<br>*M* = 20.06 (*SD* = 4.14)	n/a
147	Röttinger et al., 2021	*FoMO:*<br>Trait-State Fear of Missing Out Scale (T-SFoMOSC; Wegmann et al. 2017)<br><br>*Compulsive internet use (CIU):*<br>Compulsive Internet Use Scale (CIUS; Meerkerk et al., 2009)<br><br>*Psychopathological symptoms:*<br>Mental Health Inventory (MHI-5; Berwick et al., 1991)	Path 3	Psychopathological symptoms	CIU	State-FoMO			Male:<br>-.015, *SE* = 0.011, *p* = ns<br><br>Female:<br>-.018, *SE* = 0.013, *p* = ns	Correlational	*n* = 4973 (subsample of social media users)	Students at vocational schools aged 16-54y<br>*M* = 20.06 (*SD* = 4.14)	n/a
147	Röttinger et al., 2021	*FoMO:*<br>Trait-State Fear of Missing Out Scale (T-SFoMOSC; Wegmann et al. 2017)<br><br>*Psychopathological symptoms:*<br>Mental Health Inventory (MHI-5; Berwick et al., 1991)	Path 1	Psychopathological symptoms	Trait-FoMO		-.37	**		Correlational	*n* = 4973 (subsample of social media users)	Students at vocational schools aged 16-54y<br>*M* = 20.06 (*SD* = 4.14)	n/a
147	Röttinger et al., 2021	*FoMO:*<br>Trait-State Fear of Missing Out Scale (T-SFoMOSC; Wegmann et al. 2017)<br><br>*Psychopathological symptoms:*<br>Mental Health Inventory (MHI-5; Berwick et al., 1991)	Path 1	Psychopathological symptoms	State-FoMO		-.20	**		Correlational	*n* = 4973 (subsample of social media users)	Students at vocational schools aged 16-54y<br>*M* = 20.06 (*SD* = 4.14)	n/a
147	Röttinger et al., 2021	*FoMO:*<br>Trait-State Fear of Missing Out Scale (T-SFoMOSC; Wegmann et al. 2017)<br><br>*Compulsive internet use (CIU):*<br>Compulsive Internet Use Scale (CIUS; Meerkerk et al., 2009)	Path 2	Trait-FoMO	CIU		.45	**		Correlational	*n* = 4973 (subsample of social media users)	Students at vocational schools aged 16-54y<br>*M* = 20.06 (*SD* = 4.14)	n/a

#Ref	Study	Measures	Hypothesized model	IV	DV	M	r	*p*	Indirect effects (β/ b [CI], SE, *p*)	Study design	Sample size	Participant characteristics	Theory
147	Röttinger et al., 2021	*FoMO:*<br>Trait-State Fear of Missing Out Scale (T-SFoMOSC; Wegmann et al. 2017)<br><br>*Compulsive internet use (CIU):*<br>Compulsive Internet Use Scale (CIUS; Meerkerk et al., 2009)	Path 2	State-FoMO	CIU		.50	**		Correlational	*n* = 4973 (subsample of social media users)	Students at vocational schools aged 16-54y<br>*M* = 20.06 (*SD* = 4.14)	n/a
148	Rozgonjuk et al., 2019	*FoMO:*<br>Fear of Missing Out Scale (FoMOs; Przybylski et al., 2013)<br><br>*Interruptive notifications frequency:*<br>Self-designed questionnaire measuring frequency of receiving different types of pop-up notifications on one's smartphone (or smartwatch) (adapted from Smartphone Use Frequency Scale; Elhai et al., 2016)	Path 2	FoMO	Interruptive notifications frequency		.04	ns		Correlational	*n* = 316	Undergraduate Psychology students<br>*M* = 19.21 (*SD* = 1.74)	SDT, and, TCM
148	Rozgonjuk et al., 2019	*FoMO:*<br>Fear of Missing Out Scale (FoMOs; Przybylski et al., 2013)<br><br>*Daily activity disruptions from interruptive notifications:*<br>Self-designed question "How often do you stop or pause the following activities when you receive a pop-up notification on your smartphone (or smartwatch)?" Followed by a 20-item list of leisure and work related activities (based on Kahneman et al., 2016)	Path 2	FoMO	Daily activity disruptions from interruptive notification		.38	***		Correlational	*n* = 316	Undergraduate Psychology students<br>*M* = 19.21 (*SD* = 1.74)	SDT, and, TCM
148	Rozgonjuk et al., 2020	*FoMO:*<br>Fear of Missing Out Scale (FoMOs; Przybylski et al., 2013)<br><br>*WhatsApp use disorder:*<br>Short Smartphone Use Disorder Scale (d-KV-SSS; Montag, 2018)	Path 2	FoMO	WhatsApp use disorder		.47	***		Correlational	*n* = 748	General population<br>*M* = 38.63 (*SD* = 12.10)	I-PACE
148	Rozgonjuk et al., 2020	*FoMO:*<br>Fear of Missing Out Scale (FoMOs; Przybylski et al., 2013)<br><br>*Facebook use disorder (FBUD):*<br>Short Smartphone Use Disorder Scale (d-KV-SSS; Montag, 2018)	Path 2	FoMO	FBUD		.33	***		Correlational	*n* = 748	General population<br>*M* = 38.63 (*SD* = 12.10)	I-PACE
148	Rozgonjuk et al., 2020	*FoMO:*<br>Fear of Missing Out Scale (FoMOs; Przybylski et al., 2013)<br><br>*Instagram use disorder (FBUD):*<br>Short Smartphone Use Disorder Scale (d-KV-SSS; Montag, 2018)	Path 2	FoMO	IGUD		.46	***		Correlational	*n* = 748	General population<br>*M* = 38.63 (*SD* = 12.10)	I-PACE
148	Rozgonjuk et al., 2020	*FoMO:*<br>Fear of Missing Out Scale (FoMOs; Przybylski et al., 2013)<br><br>*Snapchat use disorder (FBUD):*<br>Short Smartphone Use Disorder Scale (d-KV-SSS; Montag, 2018)	Path 2	FoMO	SCUD		.26	**		Correlational	*n* = 748	General population<br>*M* = 38.63 (*SD* = 12.10)	I-PACE
40	Schmuck, 2021	*FoMO:*<br>Trait-State Fear of Missing Out Scale (T-SFoMOSC; Wegmann et al. 2017)<br><br>*Following social media (SM) influencers:*<br>Self-designed questionnaire assessing degree to which adolescents follow vloggers on social media	Path 4	Following SM influencers T1	FoMO T2		.30	***		Two-wave longitudinal study (4 months apart; same sample)	Wave 1 (T1)<br>*n* = 822<br><br>Wave 2 (T2)<br>*n* = 384	Wave 1 (T1)<br>Early adolescents aged 10-14y<br>*M* = 12.09 (*SD* = 1.37)<br><br>Wave 2 (T2)<br>Early adolescents aged 10-14y<br>*M* = 12.37 (*SD* = 1.48)	n/a
40	Schmuck, 2021	*FoMO:*<br>Trait-State Fear of Missing Out Scale (T-SFoMOSC; Wegmann et al. 2017)<br><br>*Social well-being:*<br>Self-designed questionnaire based on the social life subscale of KINDL questionnaire (Ravens-Sieberer & Bullinger, 1998)	Path 5	FoMO T2	Social well-being T2		-.18	***		Two-wave longitudinal study (4 months apart; same sample)	Wave 1 (T1)<br>*n* = 822<br><br>Wave 2 (T2)<br>*n* = 384	Wave 1 (T1)<br>Early adolescents aged 10-14y<br>*M* = 12.09 (*SD* = 1.37)<br><br>Wave 2 (T2)<br>Early adolescents aged 10-14y<br>*M* = 12.37 (*SD* = 1.48)	n/a
150	Scott & Woods, 2018	*FoMO:*<br>Fear of Missing Out Scale (FoMOs; Przybylski et al., 2013)<br><br>*Night time social media use (SMU):*<br>Woods & Scott, 2016	Path 2	FoMO	Nighttime SMU		.36	***		Correlational	*n* = 101	Secondary school students aged 12-18y<br>*M* = 14.0	n/a

#Ref	Study	Measures	Hypothesized model	IV	DV	M	r	*p*	Indirect effects (β/ b [CI], SE, *p*)	Study design	Sample size	Participant characteristics	Theory
112	Sela et al., 2020	*FoMO:*<br>Fear of Missing Out Scale (FoMOs; Przybylski et al., 2013)<br><br>*Objective time online (h/ day):*<br>Online activity logs gather for 14 days measuring average time spent on online activity (total, day, night)	Path 2	FoMO	Objective time online daytime		.06	ns		Correlational	*n* = 85	Adolescents aged 12-16y<br>*M* = 14.04 (*SD* = 1.09)	n/a
112	Sela et al., 2020	*FoMO:*<br>Fear of Missing Out Scale (FoMOs; Przybylski et al., 2013)<br><br>*Objective time online (h/ day):*<br>Online activity logs gather for 14 days measuring average time spent on online activity (total, day, night)	Path 2	FoMO	Objective time online nighttime		-.09	ns		Correlational	*n* = 85	Adolescents aged 12-16y<br>*M* = 14.04 (*SD* = 1.09)	n/a
112	Sela et al., 2020	*FoMO:*<br>Fear of Missing Out Scale (FoMOs; Przybylski et al., 2013)<br><br>*Objective time online (h/ day):*<br>Online activity logs gather for 14 days measuring average time spent on online activity (total, day, night)	Path 2	FoMO	Objective time online (total)		.10	ns		Correlational	*n* = 85	Adolescents aged 12-16y<br>*M* = 14.04 (*SD* = 1.09)	n/a
112	Sela et al., 2020	*FoMO:*<br>Fear of Missing Out Scale (FoMOs; Przybylski et al., 2013)<br><br>*Objective time online on pornography:*<br>Online activity logs gather for 14 days measuring average time spent on watching pornography online	Path 2	FoMO	Objective time online on pornography		.14	ns		Correlational	*n* = 85	Adolescents aged 12-16y<br>*M* = 14.04 (*SD* = 1.09)	n/a
112	Sela et al., 2020	*FoMO:*<br>Fear of Missing Out Scale (FoMOs; Przybylski et al., 2013)<br><br>Problematic internet use (PIU):<br>Generalized Problematic Internet Use Scale 2 (GPIUS 2; Caplan, 2010)	Path 2	FoMO	PIU		.24	*		Correlational	*n* = 85	Adolescents aged 12-16y<br>*M* = 14.04 (*SD* = 1.09)	n/a
112	Sela et al., 2020	*FoMO:*<br>Fear of Missing Out Scale (FoMOs; Przybylski et al., 2013)<br><br>*Objective time on social netweroking sites (SNS)*<br>Online activity logs gather for 14 days measuring average time spent on SNS	Path 2	FoMO	Objective time on SNS		.28	*		Correlational	*n* = 85	Adolescents aged 12-16y<br>*M* = 14.04 (*SD* = 1.09)	n/a
112	Sela et al., 2020	*FoMO:*<br>Fear of Missing Out Scale (FoMOs; Przybylski et al., 2013)<br><br>*Objective time on YouTube:*<br>Online activity logs gather for 14 days measuring average time spent on YouTube	Path 2	FoMO	Objective time on YouTube		.30	**		Correlational	*n* = 85	Adolescents aged 12-16y<br>*M* = 14.04 (*SD* = 1.09)	n/a
112	Sela et al., 2020	*FoMO:*<br>Fear of Missing Out Scale (FoMOs; Przybylski et al., 2013)<br><br>*Objective time online shopping*<br>Online activity logs gather for 14 days measuring average time spent on online shopping	Path 2	FoMO	Objective time on online shopping		.34	**		Correlational	*n* = 85	Adolescents aged 12-16y<br>*M* = 14.04 (*SD* = 1.09)	n/a
112	Sela et al., 2020	*FoMO:*<br>Fear of Missing Out Scale (FoMOs; Przybylski et al., 2013)<br><br>*Depression:*<br>Beck Depression Inventory (BDI; Beck, 1967)	Path 1	Depression	FoMO		.60	**		Correlational	*n* = 85	Adolescents aged 12-16y<br>*M* = 14.04 (*SD* = 1.09)	n/a
151	Servidio, 2019	*FoMO:*<br>Fear of Missing Out Scale (FoMOs; Przybylski et al., 2013)<br><br>*Smartphone use patterns (SUP):*<br>10 items about smartphone use during the last 6-months (based on Bian & Leung, 2015; Elhai et al., 2016; Jiang & Zhao, 2016)	Path 2	FoMO	SUP: information seeking		-.05	ns		Correlational	*n* = 405	University students aged 19-43y<br>*M* = 22.11 (*SD* = 3.80)	I-PACE

#Ref	Study	Measures	Hypothesized model	IV	DV	M	r	*p*	Indirect effects (β/ b [CI], SE, *p*)	Study design	Sample size	Participant characteristics	Theory
151	Servidio, 2019	*FoMO:* Fear of Missing Out Scale (FoMOs; Przybylski et al., 2013) *Smartphone use patterns (SUP):* 10 items about smartphone use during the last 6-months (based on Bian & Leung, 2015; Elhai et al., 2016; Jiang & Zhao, 2016)	Path 2	FoMO	SUP: entertainment		.11	*		Correlational	*n* = 405	University students aged 19-43y *M* = 22.11 (*SD* = 3.80)	I-PACE
151	Servidio, 2019	*FoMO:* Fear of Missing Out Scale (FoMOs; Przybylski et al., 2013) *Smartphone use patterns (SUP):* 10 items about smartphone use during the last 6-months (based on Bian & Leung, 2015; Elhai et al., 2016; Jiang & Zhao, 2016)	Path 2	FoMO	SUP: socializing		.15	**		Correlational	*n* = 405	University students aged 19-43y *M* = 22.11 (*SD* = 3.80)	I-PACE
151	Servidio, 2019	*FoMO:* Fear of Missing Out Scale (FoMOs; Przybylski et al., 2013) *Problematic smartphone use (PSU):* Smartphone Addiction Scale (SAS; De Pasquale et al., 2017; Kwon et al., 2013) *Self-control (SC):* Brief Self-Control Scale (BSCS; Tangney et al., 2004)	Path 3	SC	PSU	FoMO			-.20 [-0.309, -0.094], *SE* = .065, *p* = .002	Correlational	*n* = 405	University students aged 19-43y *M* = 22.11 (*SD* = 3.80)	I-PACE
151	Servidio, 2019	*FoMO:* Fear of Missing Out Scale (FoMOs; Przybylski et al., 2013) *Self-control (SC):* The Brief Self-Control Scale (BSCS; Tangney et al., 2004).	Path 1	SC	FoMO		-.23	***		Correlational	*n* = 405	University students aged 19-43y *M* = 22.11 (*SD* = 3.80)	I-PACE
151	Servidio, 2019	*FoMO:* Fear of Missing Out Scale (FoMOs; Przybylski et al., 2013) *Problematic smartphone use (PSU):* Smartphone Addiction Scale (SAS; De Pasquale et al., 2017; Kwon et al., 2013)	Path 2	FoMO	PSU		.35	***		Correlational	*n* = 405	University students aged 19-43y *M* = 22.11 (*SD* = 3.80)	I-PACE
152	Sha et al., 2019	*FoMO:* Fear of Missing Out Scale (FoMOs; Przybylski et al., 2013) *Facebook use disorder:* Smartphone Addiction Scale (SAS; Kwon et al., 2013)	Path 2	FoMO	Facebook use disorder		.31	**		Correlational	*n* = 2299	Smartphone owners with Facebook and WhatsApp account aged 12-75y *M* = 30.33 (*SD* = 9.80)	n/a
152	Sha et al., 2019	*FoMO:* Fear of Missing Out Scale (FoMOs; Przybylski et al., 2013) *WhatsApp use disorder:* Smartphone Addiction Scale (SAS; Kwon et al., 2013)	Path 2	FoMO	WhatsApp use disorder		.38	**		Correlational	*n* = 2299	Smartphone owners with Facebook and WhatsApp account aged 12-75y *M* = 30.33 (*SD* = 9.80)	n/a
152	Sha et al., 2019	*FoMO:* Fear of Missing Out Scale (FoMOs; Przybylski et al., 2013) *Smartphone use disorder:* Smartphone Addiction Scale (SAS; Kwon et al., 2013)	Path 2	FoMO	Smartphone use disorder		.40	**		Correlational	*n* = 2299	Smartphone owners with Facebook and WhatsApp account aged 12-75y *M* = 30.33 (*SD* = 9.80)	n/a
152	Sha et al., 2019	*FoMO:* Fear of Missing Out Scale (FoMOs; Przybylski et al., 2013) *Facebook use disorder:* Smartphone Addiction Scale (SAS; Kwon et al., 2013) *Life satisfaction (LS):* Life Satisfaction Scale (Diener et al., 1985)	Path 3	LS	Facebook use disorder	FoMO			[0.10, -0.06]	Correlational	*n* = 2299	Smartphone owners with Facebook and WhatsApp account aged 12-75y *M* = 30.33 (*SD* = 9.80)	n/a
152	Sha et al., 2019	*FoMO:* Fear of Missing Out Scale (FoMOs; Przybylski et al., 2013) *WhatsApp use disorder:* Smartphone Addiction Scale (SAS; Kwon et al., 2013) *Life satisfaction (LS):* Life Satisfaction Scale (Diener et al., 1985)	Path 3	LS	WhatsApp use disorder	FoMO			[-0.12, -0.07]	Correlational	*n* = 2299	Smartphone owners with Facebook and WhatsApp account aged 12-75y *M* = 30.33 (*SD* = 9.80)	n/a

#Ref	Study	Measures	Hypothesized model	IV	DV	M	r	*p*	Indirect effects (β/ b [CI], SE, *p*)	Study design	Sample size	Participant characteristics	Theory
152	Sha et al., 2019	*FoMO:*<br>Fear of Missing Out Scale (FoMOs; Przybylski et al., 2013)<br><br>*Smartphone use disorder:*<br>Smartphone Addiction Scale (SAS; Kwon et al., 2013)<br><br>*Life satisfaction (LS):*<br>Life Satisfaction Scale (Diener et al., 1985)	Path 3	LS	Smartphone use disorder	FoMO			[-0.12, -0.08]	Correlational	*n* = 2299	Smartphone owners with Facebook and WhatsApp account aged 12-75y<br>*M* = 30.33 (*SD* = 9.80)	n/a
69	Sheldon et al.. 2020	*FoMO:*<br>Fear of Missing Out Scale (FoMOs; Przybylski et al., 2013)<br><br>*Facebook addiction:*<br>Bergen Facebook Addiction Scale (BFAS; Andreassen et al., 2012)	Path 2	FoMO	Facebook addiction		.34	**		Correlational	*n* = 337	Undergraduate students<br>*M* = 23.35 (*SD* = 8.08)	n/a
69	Sheldon et al.. 2020	*FoMO:*<br>Fear of Missing Out Scale (FoMOs; Przybylski et al., 2013)<br><br>*Instagram addiction:*<br>Bergen Facebook Addiction Scale (BFAS; Andreassen et al., 2012)	Path 2	FoMO	Instagram addiction		.43	**		Correlational	*n* = 337	Undergraduate students<br>*M* = 23.35 (*SD* = 8.08)	n/a
69	Sheldon et al.. 2020	*FoMO:*<br>Fear of Missing Out Scale (FoMOs; Przybylski et al., 2013)<br><br>*Snapchat addiction:*<br>Bergen Facebook Addiction Scale (BFAS; Andreassen et al., 2012)	Path 2	FoMO	Snapchat addiction		.40	**		Correlational	*n* = 337	Undergraduate students<br>*M* = 23.35 (*SD* = 8.08)	n/a
113	Shoval et al., 2020	*FoMO:*<br>Fear of Missing Out Scale (FoMOs; Przybylski et al., 2013)<br><br>*Objective nighttime smartphone use:*<br>QualityTime app by Mobidays, Inc. (app that monitors type of application used, time spent using it, and time of night)	Path 4	Objective nighttime smartphone use	FoMO		.45	**		Correlational	*n* = 40	College students who use Android mobile operation system aged 19-30y<br>*M* = 23 (*SD* = 2.4)	n/a
113	Shoval et al., 2020	*FoMO:*<br>Fear of Missing Out Scale (FoMOs; Przybylski et al., 2013)<br><br>*Subjectiviely nighttime smartphone use:*<br>*Self-designed question "How often do you check your smartphone during the night?"*	Path 4	Subjectively nighttime smartphone use	FoMO		.31	ns		Correlational	*n* = 40	College students who use Android mobile operation system aged 19-30y<br>*M* = 23 (*SD* = 2.4)	n/a
53	Stead & Bibby, 2017	*FoMO:*<br>Fear of Missing Out Scale (FoMOs; Przybylski et al., 2013)<br><br>*Life satisfaction (LS):*<br>Life Satisfaction Scale (Przybylski et al., 2013)	Path 5	FoMO	LS		-.27	**		Correlational	*n* = 495	Facebook users aged 18-30y<br>*M* = 20.62 (*SD* = 1.60)	n/a
53	Stead & Bibby, 2017	*FoMO:*<br>Fear of Missing Out Scale (FoMOs; Przybylski et al., 2013)<br><br>*Problematic internet use (PIU):*<br>Problematic Use of Social Networking Sites Scale (Spraggins, 2009)	No path specified	FoMO	PIU		.47	**		Correlational	*n* = 495	Facebook users aged 18-30y<br>*M* = 20.62 (*SD* = 1.60)	n/a
53	Stead & Bibby, 2017	*FoMO:*<br>Fear of Missing Out Scale (FoMOs; Przybylski et al., 2013)<br><br>*Problematic internet use (PIU):*<br>Problematic Use of Social Networking Sites Scale (Spraggins, 2009)<br><br>*Life satisfaction (LS):*<br>Life Satisfaction Scale (Przybylski et al., 2013)	Path 6	FoMO	LS	FoMO		ns		Correlational	*n* = 495	Facebook users aged 18-30y<br>*M* = 20.62 (*SD* = 1.60)	n/a
153	Świątek et al., 2021	*FoMO:*<br>Fear of Missing Out Scale (FoMOs; Przybylski et al., 2013)<br><br>*Social media fatigue (SMF):*<br>The Social Media Fatigue Scale (SMFS; Zhang et al., 2021)<br><br>*Anxiety:*<br>The Trait Anxiety Scale (Skala Lek-Checha, SL-C; Piksa et al, 2020)	Path 3	Anxiety	SMF	FoMO				Correlational	*n* = 264	Adolescents and adults aged 14-50y<br>*M* = 23.76 (*SD* = 5.98)	n/a

#Ref	Study	Measures	Hypothesized model	IV	DV	M	r	*p*	Indirect effects (β/ b [CI], SE, *p*)	Study design	Sample size	Participant characteristics	Theory
153	Świątek et al., 2021	*FoMO:*<br>Fear of Missing Out Scale (FoMOs; Przybylski et al., 2013)<br><br>*Social media fatigue (SMF):*<br>Social Media Fatigue Scale (SMFS; Zhang et al., 2021)	Path 2	FoMO	SMF		.50	***		Correlational	*n* = 264	Adolescents and adults aged 14-50y<br>*M* = 23.76 (*SD* = 5.98)	n/a
155	Tandon et al., 2020	*FoMO:*<br>3 items (Q1, Q2, Q4) of Fear of Missing Out Scale (FoMOs; Przybylski et al., 2013)<br><br>*Compulsive social media use (SMU):*<br>Bergen Facebook Addiction Scale (Andreassen et al., 2012)	Path 2	FoMO	Compulsive SMU sample i		.31	*		Correlational	Two cohorts including<br>(i) *n* = 1398<br>(ii) *n* = 4722	(i) full-time students aged 19-27y,<br>(ii) full-time working professionals aged 18-23y	SDT
155	Tandon et al., 2020	*FoMO:*<br>3 items (Q1, Q2, Q4) of Fear of Missing Out Scale (FoMOs; Przybylski et al., 2013)<br><br>*Compulsive social media use (SMU):*<br>2 items of Bergen Facebook Addiction Scale (Andreassen et al.,	Path 2	FoMO	Compulsive SMU sample ii		.37	*		Correlational	Two cohorts:<br>(i) *n* = 1398<br>(ii) *n* = 4722	(i) full-time students aged 19-27y,<br>(ii) full-time working professionals aged 18-23y	SDT
154	Tandon, Dhir, Talwar, et al., 2021	*FoMO:*<br>5 items (Q1, Q2, Q3, Q4, Q7) of Fear of Missing Out Scale (FoMOs; Przybylski et al., 2013)<br><br>*Social media fatigue (SMF):*<br>Social media Fatigue (FSNS; Bright et al., 2015	Path 2	FoMO	SMF		.46	***		Correlational	*n* = 321	Social media users aged 18-25y	CIUT, and, SCT
154	Tandon, Dhir, Talwar, et al., 2021	*FoMO:*<br>5 items (Q1, Q2, Q3, Q4, Q7) of Fear of Missing Out Scale (FoMOs; Przybylski et al., 2013)<br><br>*Online social comparison (OSC):*<br>Lowa-Netherlands Comparison Orientation Measure (Gibbons & Buunk, 1999)	Path 2	FoMO	OSC		.72	***		Correlational	*n* = 321	Social media users aged 18-25y	CIUT, and, SCT
37	Tomczyk & Selmanagic-Lizde, 2018	*FoMO:*<br>8 items (Q1, Q3, Q4, Q5, Q6, Q7, Q8, Q10) of Fear of Missing Out Scale (FoMOs; Przybylski et al., 2013)<br><br>*Social media (SM) intensity:*<br>Social Media Intensity Scale (Ellison, Steinfield, & Lampe, 2007)	*No path specified*	FoMO	SM intensity		.54	***		Correlational	*n* = 717	School students<br>*M* = 13.0 (*SD* = 5.59)	n/a
37	Tomczyk & Selmanagic-Lizde, 2018	*FoMO:*<br>8 items (Q1, Q3, Q4, Q5, Q6, Q7, Q8, Q10) of Fear of Missing Out Scale (FoMOs; Przybylski et al., 2013)<br><br>*Social media use (SMU):*<br>Summary of Social Media Use (Hetz et al., 2015) measuring social media in inadequate situations	*No path specified*	FoMO	SMU		.56	***		Correlational	*n* = 717	School students<br>*M* = 13.0 (*SD* = 5.59)	n/a
37	Tomczyk & Selmanagic-Lizde, 2018	*FoMO:*<br>8 items (Q1, Q3, Q4, Q5, Q6, Q7, Q8, Q10) of Fear of Missing Out Scale (FoMOs; Przybylski et al., 2013)<br><br>*Social media addiction (SMA):*<br>Bergen Facebook Addiction Scale (Andreassen et al., 2012)	*No path specified*	FoMO	SMA		.56	***		Correlational	*n* = 717	School students<br>*M* = 13.0 (*SD* = 5.59)	n/a
37	Tomczyk & Selmanagic-Lizde, 2018	*FoMO:*<br>8 items (Q1, Q3, Q4, Q5, Q6, Q7, Q8, Q10) of Fear of Missing Out Scale (FoMOs; Przybylski et al., 2013)<br><br>*Social media usage (SMU) urges:*<br>Social Media Usage Urges (Abel et al., 2016)	*No path specified*	FoMO	SMU urges		.66	*		Correlational	*n* = 717	School students<br>*M* = 13.0 (*SD* = 5.59)	n/a
37	Tomczyk & Selmanagic-Lizde, 2018	*FoMO:*<br>8 items (Q1, Q3, Q4, Q5, Q6, Q7, Q8, Q10) of Fear of Missing Out Scale (FoMOs; Przybylski et al., 2013)<br><br>*Social support:*<br>Belonging Social Support Subscale of the Interpersonal Support Evaluation List (Cohen & Hoberman, 1983)	*No path specified*	FoMO	Social support		.04	ns		Correlational	*n* = 187	Adult Line *us* ers aged 21-80y<br>Median age = 39	n/a

#Ref	Study	Measures	Hypothesized model	IV	DV	M	r	*p*	Indirect effects (β/ b [CI], SE, *p*)	Study design	Sample size	Participant characteristics	Theory
61	Tsai et al., 2019	*FoMO:*<br>Fear of Missing Out Scale (FoMOs; Przybylski et al., 2013)<br><br>*Social media usage (SMU):*<br>Self-designed questionnaire assessing how much time (h/min) respondents spent using LINE on typical weekday and weekend day	*No path specified*	FoMO	SMU		.10	ns		Correlational	*n* = 187	Adult Line *us* ers aged 21-80y<br>Median age = 39	n/a
61	Tsai et al., 2019	*FoMO:*<br>Fear of Missing Out Scale (FoMOs; Przybylski et al., 2013)<br><br>*Social media (LINE use) engagement:*<br>Social Media Engagement Questionnaire (SMEQ; Przybylski et al. 2013)	*No path specified*	FoMO	Line use engagement		.28	**		Correlational	*n* = 187	Adult Line *us* ers aged 21-80y<br>Median age = 39	n/a
61	Tsai et al., 2019	*FoMO:*<br>Fear of Missing Out Scale (FoMOs; Przybylski et al., 2013)<br><br>*Depression:*<br>Center for Epidemiological Studies--Depression Scale (CES-D; Lewinsohn et al., 1997)	*No path specified*	FoMO	Depression		.32	**		Correlational	*n* = 187	Adult Line *us* ers aged 21-80y<br>Median age = 39	n/a
61	Tsai et al., 2019	*FoMO:*<br>Fear of Missing Out Scale (FoMOs; Przybylski et al., 2013)<br><br>*Loneliness:*<br>6 items from the UCLA Loneliness Scale (Wu & Yao, 2008)	*No path specified*	FoMO	Loneliness		.39	**		Correlational	*n* = 187	Adult Line *us* ers aged 21-80y<br>Median age = 39	n/a
156	Tuğtekin et al., 2020	*FoMO:*<br>Fear of Missing Out Scale (FoMOs; Przybylski et al., 2013)<br><br>*Problematic smartphone use (PSU):*<br>Nomophobia Questionnaire (NMP-Q; Yildirim & Correia, 2015)	Path 2	FoMO	PSU		.52	**		Correlational	*n* = 469	Undergraduate-level university students	n/a
156	Tuğtekin et al., 2020	*FoMO:*<br>Fear of Missing Out Scale (FoMOs; Przybylski et al., 2013)<br><br>*Social networking site (SNS) fatigue:*<br>Social Networking Site Fatigue Scale (Lee et al., 2016)	Path 2	FoMO	SNS fatigue		.23	**		Correlational	*n* = 469	Undergraduate-level university students	n/a
157	Vally et al., 2021	*FoMO:*<br>Fear of Missing Out Scale (FoMOs; Przybylski et al., 2013)<br><br>*Problematic smartphone use (PSU):*<br>Smartphone Addiction Scale-Short Version (SAS-SV; Kwon et al.,	Path 2	FoMO	PSU		.33	**		Correlational	*n* = 261	College students aged 18-36y<br>*M* = 21.51 (*SD* = 2.99)	CIUT
157	Vally et al., 2021	*FoMO:*<br>Fear of Missing Out Scale (FoMOs; Przybylski et al., 2013)<br><br>*Problematic smartphone use (PSU):*<br>Smartphone Addiction Scale-Short Version (SAS-SV; Kwon et al., 2013)<br><br>*Depression:*<br>Depression Subscale of Depression Anxiety Stress Scale-21 (DASS-	Path 3	Depression	PSU	FoMO			.14, *SE* = .04, *p* <.001	Correlational	*n* = 261	College students aged 18-36y<br>*M* = 21.51 (*SD* = 2.99)	I-PACE
157	Vally et al., 2021	*FoMO:*<br>Fear of Missing Out Scale (FoMOs; Przybylski et al., 2013)<br><br>*Problematic smartphone use (PSU):*<br>Smartphone Addiction Scale-Short Version (SAS-SV; Kwon et al., 2013)<br><br>*Anxiety:*<br>Anxiety Subscale of Depression Anxiety Stress Scale-21 (DASS-21; Lovibond & Lovibond, 1995)	Path 3	Anxiety	PSU	FoMO			.11, *SE* = .03, *p* < .001	Correlational	*n* = 261	College students aged 18-36y<br>*M* = 21.51 (*SD* = 2.99)	I-PACE
99	Varchetta et al., 2020	*FoMO:*<br>Fear of Missing Out Scale (FoMOs; Przybylski et al., 2013)<br><br>*Social media engagement (SME):*<br>Social Media Engagement Questionnaire (SMEQ: Przybylski et al, 2013)	Path 2	FoMO	SME		.43	**		Correlational	*n* = 306	University students aged 18-30y<br>*M* = 21.8 (*SD* = 3.19)	SDT

#Ref	Study	Measures	Hypothesized model	IV	DV	M	r	*p*	Indirect effects (β/ b [CI], SE, *p*)	Study design	Sample size	Participant characteristics	Theory
99	Varchetta et al., 2020	*FoMO:* Fear of Missing Out Scale (FoMOs; Przybylski et al., 2013)<br><br>*Social media addiction (SMA):* Bergen Social Media Addiction Scale (BSMAS; Andreassen et al., 2016)	Path 2	FoMO	SMA		.73	**		Correlational	*n* = 306	University students aged 18-30y *M* = 21.8 (*SD* = 3.19)	SDT
99	Varchetta et al., 2020	*FoMO:* Fear of Missing Out Scale (FoMOs; Przybylski et al., 2013)<br><br>*Psychological need satisfaction (PSN):* Basic Psychological Needs Scale (BPNS; La Guardia et al., 2000)	Path 1	PNS - Autonomy	FoMO		-.50	**		Correlational	*n* = 306	University students aged 18-30y *M* = 21.8 (*SD* = 3.19)	SDT
99	Varchetta et al., 2020	*FoMO:* Fear of Missing Out Scale (FoMOs; Przybylski et al., 2013)<br><br>*Psychological need satisfaction (PSN):* Basic Psychological Needs Scale (BPNS; La Guardia et al., 2000)	Path 1	PNS - Competence	FoMO		-.46	**		Correlational	*n* = 306	University students aged 18-30y *M* = 21.8 (*SD* = 3.19)	SDT
99	Varchetta et al., 2020	*FoMO:* Fear of Missing Out Scale (FoMOs; Przybylski et al., 2013)<br><br>*Psychological need satisfaction (PSN):* Basic Psychological Needs Scale (BPNS; La Guardia et al., 2000)	Path 1	PNS - Support	FoMO		-.43	**		Correlational	*n* = 306	University students aged 18-30y *M* = 21.8 (*SD* = 3.19)	SDT
99	Varchetta et al., 2020	*FoMO:* Fear of Missing Out Scale (FoMOs; Przybylski et al., 2013)<br><br>*Self-esteem:* Rosenberg Self-Esteem Scale (RSE; Rosenberg, 1965)	Path 5	FoMO	Self-esteem		-.38	**		Correlational	*n* = 306	University students aged 18-30y *M* = 21.8 (*SD* = 3.19)	n/a
159	Weaver & Swank, 2021	*FoMO:* Fear of Missing Out Scale (FoMOs; Przybylski et al., 2013)<br><br>*Problematic social media use (PSMU):* Social Media Use Questionnaire (Xanidis & Brignell, 2016)	Path 2	FoMO	PSMU		.48	**		Correlational	*n* = 278	Undergraduate students aged 18-53y *M* = 20.5 (SD = 5.4)	CIUT, and, SCT
159	Weaver & Swank, 2021	*FoMO:* Fear of Missing Out Scale (FoMOs; Przybylski et al., 2013)<br><br>*Self-esteem:* Rosenberg Self-Esteem Scale (RSE; Rosenberg, 1965)	Path 5	FoMO	Self-esteem		-.21	**		Correlational	*n* = 278	Undergraduate students aged 18-53y *M* = 20.5 (SD = 5.4)	n/a
159	Weaver & Swank, 2021	*FoMO:* Fear of Missing Out Scale (FoMOs; Przybylski et al., 2013)<br><br>*Life satisfaction (LS):* Satisfaction With Life Scale (Diener et al., 1985)	Path 5	FoMO	LS		-.29	**		Correlational	*n* = 278	Undergraduate students aged 18-53y *M* = 20.5 (SD = 5.4)	n/a
30	Wegmann et al., 2017	*State-FoMO:* 2 items (Q8 + Q10) of Fear of Missing Out Scale (FoMOs; Przybylski et al., 2013) + 5 self-designed additional items<br><br>*Interpersonal sensitivity:* Subscales Interpersonal Sensitivity of the Brief Symptom Inventory (Boulet & Boss, 1991; Derogatis, 1993)	Path 1	Interpersonal sensitivity	State-FoMO		.17	**		Correlational	*n* = 270	Individuals aged 17-39y *M* = 23.43 (*SD* = 4.02)	I-PACE
30	Wegmann et al., 2017	*State-FoMO:* 2 items (Q8 + Q10) of Fear of Missing Out Scale (FoMOs; Przybylski et al., 2013) + 5 self-designed additional items<br><br>*Internet communication disorder (ICD):* Modified version of the Short Internet Addiction Test for Internet communication disorder (s-IAT-ICD; Wegmann et al., 2015)<br><br>*Psychopathological symptoms:* Subscales Depression and Interpersonal Sensitivity of the Brief Symptom Inventory (Boulet & Boss, 1991; Derogatis, 1993)	Path 3	Psychopathological symptoms	ICD	State-FoMO			.02, *SE* = .008, *p* = .03	Correlational	*n* = 270	Individuals aged 17-39y *M* = 23.43 (*SD* = 4.02)	I-PACE

#Ref	Study	Measures	Hypothesized model	IV	DV	M	r	*p*	Indirect effects (β/ b [CI], SE, *p*)	Study design	Sample size	Participant characteristics	Theory
30	Wegmann et al., 2017	*Trait-FoMO:*<br>5 items (Q1, Q2, Q3, Q4, Q9) of Fear of Missing Out Scale (FoMOs; Przybylski et al., 2013)<br><br>*Positive reinforcement:*<br>The Internet-Use Expectancies Scale (IUES; Brand et al., 2014)	Path 2	Trait-FoMO	Positive reinforcement		.08	ns		Correlational	*n* = 270	Individuals aged 17-39y<br>*M* = 23.43 (*SD* = 4.02)	I-PACE
30	Wegmann et al., 2017	*State-FoMO:*<br>2 items (Q8 + Q10) of Fear of Missing Out Scale (FoMOs; Przybylski et al., 2013) + 5 self-designed additional items<br><br>*Depression:*<br>Subscales Depression of the Brief Symptom Inventory (Boulet & Boss, 1991; Derogatis, 1993)	Path 1	Depression	State-FoMO		.10	ns		Correlational	*n* = 270	Individuals aged 17-39y<br>*M* = 23.43 (*SD* = 4.02)	I-PACE
30	Wegmann et al., 2017	*Trait-FoMO:*<br>5 items (Q1, Q2, Q3, Q4, Q9) of Fear of Missing Out Scale (FoMOs; Przybylski et al., 2013)<br><br>*Negative reinforcement:*<br>The Internet-Use Expectancies Scale (IUES; Brand et al., 2014)	Path 2	Trait-FoMO	Negative reinforcement		.16	**		Correlational	*n* = 270	Individuals aged 17-39y<br>*M* = 23.43 (*SD* = 4.02)	I-PACE
30	Wegmann et al., 2017	*Trait-FoMO:*<br>5 items (Q1, Q2, Q3, Q4, Q9) of Fear of Missing Out Scale (FoMOs; Przybylski et al., 2013)<br><br>*Depression:*<br>Subscales Depression of the Brief Symptom Inventory (Boulet & Boss, 1991; Derogatis, 1993)	Path 1	Depression	Trait-FoMO		.17	**		Correlational	*n* = 270	Individuals aged 17-39y<br>*M* = 23.43 (*SD* = 4.02)	I-PACE
30	Wegmann et al., 2017	*Trait-FoMO:*<br>5 items (Q1, Q2, Q3, Q4, Q9) of Fear of Missing Out Scale (FoMOs; Przybylski et al., 2013)<br><br>*Internet communication disorder (ICD):*<br>Modified version of the Short Internet Addiction Test for Internet communication disorder (s-IAT-ICD; Wegmann et al., 2015)	Path 2	Trait-FoMO	ICD		.21	**		Correlational	*n* = 270	Individuals aged 17-39y<br>*M* = 23.43 (*SD* = 4.02)	I-PACE
30	Wegmann et al., 2017	*Trait-FoMO:*<br>5 items (Q1, Q2, Q3, Q4, Q9) of Fear of Missing Out Scale (FoMOs; Przybylski et al., 2013)<br><br>*Interpersonal sensitivity:*<br>Subscales Interpersonal Sensitivity of the Brief Symptom Inventory (Boulet & Boss, 1991; Derogatis, 1993)	Path 1	Interpersonal sensitivity	Trait-FoMO		.29	**		Correlational	*n* = 270	Individuals aged 17-39y<br>*M* = 23.43 (*SD* = 4.02)	I-PACE
30	Wegmann et al., 2017	*State-FoMO:*<br>2 items (Q8 + Q10) of Fear of Missing Out Scale (FoMOs; Przybylski et al., 2013) + 5 self-designed additional items<br><br>*Negative reinforcement:*<br>The Internet-Use Expectancies Scale (IUES; Brand et al., 2014)	Path 2	State-FoMO	Negative reinforcement		.30	**		Correlational	*n* = 270	Individuals aged 17-39y<br>*M* = 23.43 (*SD* = 4.02)	I-PACE
30	Wegmann et al., 2017	*State-FoMO:*<br>2 items (Q8 + Q10) of Fear of Missing Out Scale (FoMOs; Przybylski et al., 2013) + 5 self-designed additional items<br><br>*Positive reinforcement:* The Internet-Use Expectancies Scale (IUES; Brand et al., 2014)	Path 2	State-FoMO	Positive reinforcement		.37	**		Correlational	*n* = 270	Individuals aged 17-39y<br>*M* = 23.43 (*SD* = 4.02)	I-PACE
30	Wegmann et al., 2017	*State-FoMO:*<br>2 items (Q8 + Q10) of Fear of Missing Out Scale (FoMOs; Przybylski et al., 2013) + 5 self-designed additional items<br><br>*Internet communication disorder (ICD):*<br>Modified version of the Short Internet Addiction Test for Internet communication disorder (s-IAT-ICD; Wegmann et al., 2015)	Path 2	State-FoMO	ICD		.46	**		Correlational	*n* = 270	Individuals aged 17-39y<br>*M* = 23.43 (*SD* = 4.02)	I-PACE

#Ref	Study	Measures	Hypothesized model	IV	DV	M	r	*p*	Indirect effects (β/ b [CI], SE, *p*)	Study design	Sample size	Participant characteristics	Theory
84	Wegmann et al., 2021	*FoMO:*<br>Trait-State Fear of Missing Out Scale (T-SFoMOSC; Wegmann et al. 2017)<br><br>*Need to belong:*<br>Need to Belong Scale (Baumeister & Leary, 1995)	Path 1	Need to belong	Trait-FoMO		.38	**		Correlational	*n* = 719	Individuals aged 18-79y<br>*M* = 50.11 (*SD* = 12.29)	I-PACE
84	Wegmann et al., 2021	*FoMO:*<br>Trait-State Fear of Missing Out Scale (T-SFoMOSC; Wegmann et al. 2017)<br><br>*Need to belong:*<br>Need to Belong Scale (Baumeister & Leary, 1995)	Path 1	Need to belong	State FoMO		.34	**		Correlational	*n* = 719	Individuals aged 18-79y<br>*M* = 50.11 (*SD* = 12.29)	I-PACE
84	Wegmann et al., 2021	*FoMO:*<br>Trait-State Fear of Missing Out Scale (T-SFoMOSC; Wegmann et al. 2017)<br><br>*Problematic use of social networks:*<br>Modified version of the short Internet Addiction Test (s-IAT-com) for online-communication applications (Wegmann et al., 2015)	Path 2	Trait-FoMO	Problematic use of social networks		.43	**		Correlational	*n* = 719	Individuals aged 18-79y<br>*M* = 50.11 (*SD* = 12.29)	I-PACE
84	Wegmann et al., 2021	*FoMO:*<br>Trait-State Fear of Missing Out Scale (T-SFoMOSC; Wegmann et al. 2017)<br><br>*Problematic use of social networks:*<br>Modified version of the short Internet Addiction Test (s-IAT-com) for online-communication applications (Wegmann et al., 2015)	Path 2	State-FoMO	Problematic use of social networks		.51	**		Correlational	*n* = 719	Individuals aged 18-79y<br>*M* = 50.11 (*SD* = 12.29)	I-PACE
101	Wolniewicz et al., 2018	*FoMO:*<br>Fear of Missing Out Scale (FoMOs; Przybylski et al., 2013)<br><br>*Fear of positive evaluation:*<br>Fear of Positive Evaluation Scale (FPES; Weeks et al., 2008)	Path 5	FoMO	Fear of positive evaluation		.12	*		Correlational	*n* = 296	College students<br>*M* = 20.0 (*SD* = 3.02)	UGT, and, CIUT
101	Wolniewicz et al., 2018	*FoMO:*<br>Fear of Missing Out Scale (FoMOs; Przybylski et al., 2013)<br><br>*Process and social smartphone use:*<br>Smartphone Usage (Elhai et al., 2016)	Path 2	FoMO	Process use		.12	*		Correlational	*n* = 296	College students<br>*M* = 20.0 (*SD* = 3.02)	UGT, and, CIUT
101	Wolniewicz et al., 2018	*FoMO:*<br>Fear of Missing Out Scale (FoMOs; Przybylski et al., 2013)<br><br>*Process and social smartphone use:*<br>Smartphone Usage (Elhai et al., 2016)	Path 2	FoMO	Social use		.19	**		Correlational	*n* = 296	College students<br>*M* = 20.0 (*SD* = 3.02)	UGT, and, CIUT
101	Wolniewicz et al., 2018	*FoMO:*<br>Fear of Missing Out Scale (FoMOs; Przybylski et al., 2013)<br><br>*Negative affect:*<br>Positive Negative Affect Scale (PANAS; Watson et al., 1988)	Path 5	FoMO	Negative affect		.22	***		Correlational	*n* = 296	College students<br>*M* = 20.0 (*SD* = 3.02)	UGT, and, CIUT
101	Wolniewicz et al., 2018	*FoMO:*<br>Fear of Missing Out Scale (FoMOs; Przybylski et al., 2013)<br><br>*Fear of negative evaluation:*<br>Brief Fear of Negative Evaluation Scale (BFNES-S; Rodebaugh et al., 2004)	Path 1	FoMO	Fear of negative evaluation		.29	***		Correlational	*n* = 296	College students<br>*M* = 20.0 (*SD* = 3.02)	UGT, and, CIUT
101	Wolniewicz et al., 2018	*FoMO:*<br>Fear of Missing Out Scale (FoMOs; Przybylski et al., 2013)<br><br>*Problematic smartphone use (PSU):*<br>Smartphone Addiction Scale (SAS-SV ; Kwon et al., 2013)	Path 2	FoMO	PSU		.42	***		Correlational	*n* = 296	College students<br>*M* = 20.0 (*SD* = 3.02)	UGT, and, CIUT
103	Wolniewicz et al., 2019	*FoMO:*<br>Fear of Missing Out Scale (FoMOs; Przybylski et al., 2013)<br><br>*Smartphone use frequency (SUF):*<br>Smartphone Use Frequency Scale (Elhai et al., 2016)	Path 2	FoMO	SUF		.18	**		Correlational	*n* = 297	College students<br>*M* = 19.70 (*SD* = 3.96)	UGT, and, I-PACE

#Ref	Study	Measures	Hypothesized model	IV	DV	M	r	*p*	Indirect effects (β/ b [CI], SE, *p*)	Study design	Sample size	Participant characteristics	Theory
103	Wolniewicz et al., 2019	*FoMO:*<br>Fear of Missing Out Scale (FoMOs; Przybylski et al., 2013)<br><br>*Problematic smartphone use (PSU):*<br>Smartphone Addiction Scale (Kwon et al., 2013)	Path 2	FoMO	PSU		.53	***		Correlational	*n* = 297	College students<br>*M* = 19.70 (*SD* = 3.96)	UGT, and, I-PACE
160	Yang et al., 2021	*FoMO:*<br>Fear of Missing Out Scale (FoMOs; Przybylski et al., 2013)<br><br>*Smartphone use frequency (SUF):*<br>Smartphone Use Frequency Scale (Elhai et al., 2016)<br><br>*Stress:*<br>Stress subscale of Depression Anxiety Stress Scale-21 (DASS-21; Lovibond & Lovibond, 1995)	Path 2	FoMO	SUF		.22	***		Correlational	*n* = 2263	University students<br>*M* = 19.35 (*SD* = 1.36)	UGT, and, I-PACE
160	Yang et al., 2021	*FoMO:*<br>Fear of Missing Out Scale (FoMOs; Przybylski et al., 2013)<br><br>*Problematic smartphone use (PSU):*<br>Smartphone Addiction Scale-Short Version (SAS-SV, Distefano & Morgan, 2014)	Path 2	FoMO	PSU		.37	***		Correlational	*n* = 2263	University students<br>*M* = 19.35 (*SD* = 1.36)	UGT, and, I-PACE
160	Yang et al., 2021	*FoMO:*<br>Fear of Missing Out Scale (FoMOs; Przybylski et al., 2013)<br><br>*Problematic smartphone use (PSU):*<br>Smartphone Addiction Scale-Short Version (SAS-SV, Distefano & Morgan, 2014)<br><br>*Stress:*<br>Stress subscale of Depression Anxiety Stress Scale-21 (DASS-21; Lovibond & Lovibond, 1995)	Path 3	Stress	PSU	FoMO			.04 [0.005 - 0.068], *SE* = .016, *p* = 0.022	Correlational	*n* = 2263	University students<br>*M* = 19.35 (*SD* = 1.36)	UGT, and, I-PACE
161	Yin et al., 2015	*FoMO:*<br>7 items (Q2, Q4, Q5, Q6, Q7, Q8, Q10) of Fear of Missing Out Scale (FoMOs; Przybylski et al., 2013)<br><br>*Continuance intention* :<br>Continuance intention of using Social Networking Sites (Bhattacherjee et al., 2008)	Path 2	FoMO	Continuance intention		.32	***		Correlational	*n* = 629	87.4% between 20-40y	n/a
64	Yin et al., 2019	*FoMO:*<br>Fear of Missing Out Scale (FoMOs; Przybylski et al., 2013)<br><br>*Social networking site (SNS) addiction:*<br>Adapted version of the Facebook Intrusion Questionnaire (FIQ; Elphinston and Noller 2011)	Path 4	SNS addiction	FoMO		.41	***		Correlational	*n* = 704	Senior high school students aged 15-18y<br>*M* = 16.80 (*SD* = 0.92)	n/a
38	Zhou, 2018	*FoMO:*<br>6 items (only Q3 + Q5 were specified) of Fear of Missing Out Scale (FoMOs; Przybylski et al., 2013)<br><br>*WeChat use:*<br>Self-designed questionnaire assessing frequency of using WeChat for WeChat interpersonal use	Path 2	FoMO	WeChat interpersonal		.16	***		Correlational	*n* = 687	University students<br>*M* = 21.14 (*SD* = 2.30)	n/a
38	Zhou, 2018	*FoMO:*<br>6 items (only Q3 + Q5 were specified) of Fear of Missing Out Scale (FoMOs; Przybylski et al., 2013)<br><br>*WeChat use moments:*<br>Self-designed questionnaire assessing frequency of using WeChat for WeChat moment use	Path 2	FoMO	WeChat use moments		.20	***		Correlational	*n* = 687	University students<br>*M* = 21.14 (*SD* = 2.30)	n/a
38	Zhou, 2018	*FoMO:*<br>6 items (only Q3 + Q5 were specified) of Fear of Missing Out Scale (FoMOs; Przybylski et al., 2013)<br><br>*WeChat use group:*<br>Self-designed questionnaire assessing frequency of using WeChat for WeChat group use	Path 2	FoMO	WeChat use group		.20	***		Correlational	*n* = 687	University students<br>*M* = 21.14 (*SD* = 2.30)	n/a

#Ref	Study	Measures	Hypothesized model	IV	DV	M	r	*p*	Indirect effects (β/ b [CI], SE, *p*)	Study design	Sample size	Participant characteristics	Theory
38	Zhou, 2018	*FoMO:*<br>6 items (only Q3 + Q5 were specified) of Fear of Missing Out Scale (FoMOs; Przybylski et al., 2013)<br><br>*Permanently online:*<br>Self-designed item measuring frequency of being online in social situations (e.g., in the classroom/lecture, at home, on public transportation, etc.) (based on Vorderer et al., 2016)	Path 2	FoMO	Permanently online		.26	***		Correlational	*n* = 687	University students<br>M = 21.14 (SD = 2.30)	n/a

Note

measures In case the Fear of Missing Out Scale (FoMOs; Przybyslki et al., 2013) is utilized, all ten items of the scale were measured. Otherwise, the specific items that were included are explicitly stated

measures FoMOs: Q1) I fear others have more rewarding experiences than me, Q2) I fear my friends have more rewarding experiences than me, Q3) I get worried when I find out my friends are having fun without me, Q4) I get anxious when I don't know what my friends are up to, Q5) It is important that I understand my friends "in jokes", Q6) Sometimes, I wonder if I spend too much time keeping up with what is going on, Q7) It bothers me when I miss an opportunity to meet up with friends, Q8) When I have a good time it is important for me to share the details online (e.g., updating status), Q9) When I miss out on a planned get-together it bothers me, Q10) When I go on vacation, I continue to keep tabs on what my friends are doing.

Hypothesized model Path 1: Psychological well-being > FoMO; Path 2: FoMO > Digital technology use; Path 3: Psychological well-being > FoMO > Digital technology use; Path 4: Digital technology use > FoMO; Path 5: FoMO > Psychological well-being; Path 6: Digital technology use > FoMO > Psychological well-being

IV, DV, M IV = independent variable; DV = dependent variable; M = mediator

r, p Correlation coefficient (r) and *p* -value are reported. In data sets that reported other measures (e.g., t-value, F-value), those measures were converted to correlation coefficients by following the common guidelines for meta analysis (Lenhard & Lenhard, 2022; Lipsey & Wilson, 2000; Ruscio, 2008)

Theory Theory used for the hypothesized path. N/a = no theory was utilized; CLT = Cognitive Load Theory (Sweller, 1988); SDT = Self-determination theory (Deci & Ryan, 1985); I-PACE = Interaction of Person-Affect-Cognition-Expectation model (Brand et al., 2016); UGT = Uses and Gratifications Theory (Blumler & Katz, 1974); SCT = Social Comparison Theory (Festinger, 1954); DSMM = Differential Susceptibility to Media Effects Model (Valkenburg & Peter, 2013); CIUT = Compensatory Internet Use Theory (Kardefelt-Winthers, 2014); SSO = The stressor–strain–outcome model (Koeske & Koeske, 1993); TCM = Threaded Cognition Model (Salvucci & Taatgen, 2008)
